# Formulation Development of Natural Polymeric Nanoparticles, In Vitro Antiaging Evaluation, and Metabolite Profiling of *Toona sinensis* Leaf Extracts

**DOI:** 10.3390/ph18030288

**Published:** 2025-02-20

**Authors:** Uce Lestari, Muhaimin Muhaimin, Anis Yohana Chaerunisaa, Wawan Sujarwo

**Affiliations:** 1 Doctoral Program of Pharmacy, Faculty of Pharmacy, Universitas Padjadjaran, Jalan Raya Bandung–Sumedang Km 21, Jatinangor 45363, Indonesia; 2 Department of Pharmacy, Faculty of Medicine and Health Sciences, Universitas Jambi, Jalan Jambi–Muara Bulian Km 15, Mendalo Indah 36361, Indonesia; 3 Department of Pharmaceutical Biology, Faculty of Pharmacy, Universitas Padjadjaran, Jalan Raya Bandung–Sumedang Km 21, Jatinangor 45363, Indonesia; 4 Center of Herbal Studies, Universitas Padjadjaran, Jalan Raya Bandung–Sumedang Km 21, Jatinangor 45363, Indonesia; 5 Department of Pharmaceutics and Pharmaceutical Technology, Faculty of Pharmacy, Universitas Padjadjaran, Jalan Raya Bandung–Sumedang Km 21, Jatinangor 45363, Indonesia; 6 Research Center for Ecology and Ethnobiology, National Research and Innovation Agency (BRIN), Cibinong 16911, Indonesia

**Keywords:** natural polymers, nanoparticles, *Toona sinensis*, elastase enzyme, metabolite profiling

## Abstract

**Background/Objectives:** Natural polymer nanoparticles have potential as delivery systems, can enhance pharmacological activity, and can improve stability in the cosmetic field. In this research, we implemented a development approach for chitosan–alginate and chitosan–pectin nanoparticles. This study aimed to investigate effect of formulation, process variables, in vitro antiaging evaluation, and metabolite profiling of *Toona sinensis* leaf extracts. **Methods**: Polymeric nanoparticles have been prepared using the ionic gelation method (Temperature = 40 °C, time = 1 h and speed = 1000 rpm), in vitro antiaging evaluation using the Neutrophil Elastase Inhibitor Screening Kit method, and analysis of metabolite profiling with UHPLC–HRMS. **Results**: Research results found that the SLE and EAFSL nanoparticles that have good and stable characteristics before and after storage in a climatic chamber after 3 months are FIIA-NPSLE (0.75% chitosan and 1.25% Alginate), FIP-NPSLE (1% chitosan and 0.5% Pectin), FIIA-NPEAFSL (0.75% chitosan and 1.25% Alginate), and FIIIP-NPEAFSL (0.125% chitosan and 0.375% Alginate). Chitosan–alginate polymers, such as FIIA-NPEAFSL, have higher inhibition of the elastase enzyme than FIIA-NPSLE, with a % inhibition (IC_50_) of FIIA-NPEAFSL being 87.30%, while the IC_50_ of FIIA-NPSLE is 39.40%. Meanwhile, using chitosan–pectin polymers, such as FIP-NPSLE, results in lower inhibition of the elastase enzyme compared to the chitosan–alginate polymer, with an IC_50_ of 27.28% while IC_50_ FIIIP-NPEAFSL is 39.53%. SLE and EAFSL nanoparticles with chitosan–alginate and chitosan–pectin polymers resulted in a significant PDI during storage from 1.3 to 1.9, and zeta potential values were very low, ranging from −11 mV to −27 mV. Metabolite profiling using UHPLC–HRMS on *T. sinensis* leaf extracts revealed that the main compounds contained were glycitein, quercetin, quercetin-3β-D-glucoside, kaempferol, and ellagic acid, which has potential as an antiaging agent. **Conclusions**: It can be concluded that using chitosan, alginate, and pectin in the process of encapsulating extracts into nanoparticles with the same process variables affect evaluation of antiaging activity in elastase enzymes. Further research will develop these nanoparticles into nanohydrogels with antiaging activity.

## 1. Introduction

Surian leaves (*Toona sinensis*) are plants that are widely found in Indonesia and have the potential to function as a source of natural antioxidants and antiaging. *Toona sinensis* are widely used as a traditional medicine for skin problems, for dysentery, to dissolve phlegm, as a tonic, and to lower blood sugar [1]. Limonoids, phytols, flavonoids, essential oils, triterpenoids, phenols, and catechins are the secondary metabolites of the *Toona sinensis* plant [2,3]. These have the ability to prevent various body diseases caused by oxidative stress associated with aging and inflammation [4]. This very strong antioxidant activity works synergistically against the ability to stabilize the role of ROS in the photoaging process, so that the ethanol extract and the active fraction of Surian leaves (*Toona sinensis*) can be developed in the form of pharmaceutical preparations in relation to antiaging in the form of nanoparticles.

Nanoparticles have an important role in the delivery of bioactive compounds within Surian leaf extracts, especially in the pharmaceutical field, including increasing bioavailability by increasing the solubility and stability of the bioactive compounds so that they can increase bioavailability in the body [5]. In addition, as a targeted delivery, nanoparticles provide protection of bioactive compounds from degradation due to environmental factors (pH, enzymes, light), controlled release so that the pharmacological effects are longer and more efficient, and a better penetrative ability so that they can penetrate cell membranes to reach therapeutic targets that are difficult to reach. Based on these advantages, nanoparticles are a promising technology in the development of pharmaceutical preparations [6].

The process of preparing nanoparticles typically involves polymers that come from nature. The use of natural polymers such as chitosan, alginate, and pectin in nanoparticle production has become the primary choice. This is due to their unique properties that are well suited for their respective applications [7]. Chitosan is derived from chitin, which is commonly found in the exoskeletons of arthropods such as crabs, crayfish, shrimp, and lobsters. The concentration of chitosan typically ranges from 0.1% *w/v* to 2% *w*/*v*, depending on the production method and intended application. Alginate is a polymer extracted from brown algae [7,8]. The commonly used concentration of alginate ranges from 0.5% to 3%. Meanwhile, pectin is extracted from fruits and vegetables, with the usual concentration ranging from 0.1% to 1%. The concentration of these natural polymers plays a crucial role in determining the particle size, morphology, and stability of the resulting nanoparticles [8]. Choosing the appropriate concentration for each polymer allows for the adjustment of the physicochemical properties of the nanoparticles, supporting their successful application in pharmaceuticals, drug delivery, and various other industries [7,8].

The use of natural polysaccharides or hydrophilic polymers mentioned above as drug carriers or active substances has been the main focus in the development of innovative drug delivery systems [9]. Chitosan possesses hydrophilic properties that allow the formation of a polymer matrix capable of efficiently containing drugs. Apart from that, alginate, with its hydrophilic nature, can form hydrogels when interacting with calcium ions [9,10]. This enables sustained and controlled release of the active substance over the desired period. Furthermore, pectin can be used to form an environmentally friendly hydrophilic matrix. The advantages of these three hydrophilic or natural polymers, such as biodegradability and biocompatibility, make them ideal as drug carriers in medical and pharmaceutical applications [10]. Through careful and precise formulation, these natural hydrophilic polymers can enhance the stability and effectiveness of drug delivery, making them a promising primary choice in the development of innovative and efficient drug delivery systems [10,11].

Natural polymers or hydrophilic substances, such as chitosan, alginate, and pectin, have both advantages and disadvantages in their roles as drug carriers or active substances as an efficient and stable drug carrier [11]. Their advantages include hydrophilic properties that facilitate excellent interaction with water, allowing controlled and slow drug release. For instance, chitosan can form a stable polymer matrix, enhancing drug solubility and bioavailability [12,13,14]. Alginate can form hydrogels, enabling gradual drug release, while pectin, with its gelling property, provides effective release control [12,13,14,15]. However, their weaknesses encompass limited mechanical stability, restricting their application in certain contexts. Additionally, they are highly sensitive to environmental factors, such as specific pH levels or ion content, which can influence the characteristics of these drug carriers. Therefore, the careful selection of these natural polymers or hydrophilic substances, along with precise formulation design, is crucial to maximize benefits and overcome limitations in the development of an effective drug delivery system, and there is an increased emphasis on environmentally friendly and biodegradable materials in this pursuit [12,13,14]. Overall, these natural polymers play a crucial role in the development of biomedical technology, providing innovative solutions for controlled drug delivery, tissue regeneration, and other therapeutic applications in the context of health and medical care [16].

One of the most extensively researched and adopted methods for nanoparticle production is the polymer nanoparticle synthesis through the ionic gelation method. The ionic gelation method has gained intense attention, particularly in the context of biomedical applications. Ionic gelation involves the use of a polymer solution and an ion acting as a gelling agent, such as calcium or zinc [17,18,19,20]. This process leverages the ionic interaction between the gelling agent and the polymer, resulting in a gel structure that can be utilized for nanoparticle formation. The development of this method has been a focal point of research due to its ability to produce nanoparticles with precisely controlled sizes and properties [20,21]. Further research in the ionic gelation method focuses on optimizing parameters such as solution concentration, the ratio of gelling agent ions to the polymer, and gelation conditions to yield nanoparticles with optimal performance and stability. The success of this method in producing nanoparticles with high control has propelled various applications across different fields, including drug delivery and biomedical diagnostics [18].

In this study, the ionic gelation method was utilized because it offers significant advantages in nanoparticle production compared to other methods when combined with natural polymers like chitosan, alginate, and pectin, using NaTPP (sodium tripolyphosphate) as a cross-linker [22,23,24]. Natural polymers such as chitosan, alginate, and pectin possess biodegradable and biocompatible properties, making the resulting nanoparticles more environmentally friendly and safe for biomedical applications [19,25,26,27,28]. Ionic gelation allows for efficient and controlled nanoparticle formation, resulting in uniform particle size and perfect distribution. The use of the NaTPP cross-linker enhances the structural stability of nanoparticles, making them more resistant to environmental changes and providing mucoadhesive properties that strengthen interactions with target cells [20]. This method ensures better control over the physicochemical properties of nanoparticles, enabling broader applications in drug delivery, gene therapy, and other fields [19,26,29,30].

This research aims to investigate the influence of formulation variables, particularly the types and concentrations of polymers, on the formation of chitosan–alginate and chitosan–pectin nanoparticles [20,31,32,33,34]. In terms of formulation aspects, this study delves into how variations in polymer types (chitosan, alginate, and pectin) and changes in the concentration of each polymer can affect the physicochemical properties of the resulting nanoparticles. This includes particle size, polydispersity index (PDI), zeta potential, encapsulation efficiency (% EE), and stability during storage [15,32,33,34,35]. Additionally, the research aims to understand the impact of formulation variables on the morphology of nanoparticles and their potential application as antiaging agents by assessing their inhibitory activity against elastase enzymes. By focusing on the influence of polymer types and concentrations in the formulation, it is expected that this study will provide deeper insights to optimize the design of chitosan–alginate and chitosan–pectin nanoparticles for antiaging purposes.

## 2. Results and Discussion

### 2.1. Extraction and Fractionation of Surian Leaves

Extraction and fractionation methods have many advantages, including the ability to specifically separate bioactive compounds, increase the concentration of desired compounds, and remove unnecessary impurities. This method has several limitations in that it has high costs, requires sophisticated equipment, and takes longer to achieve the best results. The extraction and fractionation process can cause some bioactive compounds to become ineffective due to operational conditions such as solvents, temperature, and pressure, which can affect the efficiency of this method [26,36,37].

The ethanol extract from Surian leaves was obtained in the amount of 1.99 kg from an initial raw material weight of 5.98 kg. In this study, the extract yield was found to be 33.278%, exceeding the expected 7.2%. This is due to the characteristics of the extraction process, where the initial extraction process dissolves almost all compounds that can be dissolved in the solvent used, including polar, semi-polar, and non-polar compounds, thus, producing a much higher extract yield. Meanwhile, the yield of the ethyl acetate fraction from Surian leaves was 0.526%, falling short of the expected 7.2% as per the Herbal Pharmacopoeia. This is because the ethyl acetate fraction is formed through more selective fractionation, where only certain compounds (usually semi-polar) can dissolve in ethyl acetate. As a result, the number of compounds obtained from fractionation is less than the initial extraction, resulting in a lower fraction yield. According to the Herbal Pharmacopoeia, the yield limit produced is not less than 7.2% [38]. A lower ethyl acetate fraction yield indicates that the solvent used is capable of dissolving many bioactive compounds, while an extract yield exceeding 7.2% indicates that the solvent is capable of dissolving large amounts of bioactive compounds. Yield is a crucial parameter in evaluating the efficiency of an extraction method. The higher the yield is, the more efficient the extraction method will be in isolating the desired compounds. Additionally, the extract yield plays a significant role in determining the economic value of a natural substance, as it can influence the availability and price of the material. Therefore, extract yield becomes a key factor in optimizing the extraction process and maximizing the benefits of the natural material used. The low yield of the ethyl acetate fraction is attributed to the incomplete fractionation of the entire extract [30,35].

The use of different solvents can affect the secondary metabolite profile of Surian leaf extract because each solvent has a different polarity, so it is able to extract certain secondary metabolite compounds with varying efficiency. Polar solvents such as methanol and ethanol tend to extract water-soluble phenolic and flavonoid compounds, and semi-polar solvents such as ethyl acetate extract semi-polar secondary metabolites, while non-polar solvents such as n-hexane are more effective in extracting terpenoid and lipid compounds. These differences in solvents not only affect the type and amount of bioactive compounds obtained but also the biological activity of the extract, such as antioxidant, anti-inflammatory, and antimicrobial activities. Therefore, choosing the right solvent is very important to obtain an extract with an optimal metabolite composition according to the research objectives or desired applications [26].

### 2.2. Analysis of Metabolite Profiling with UHPLC–HRMS

**Figure 1:**
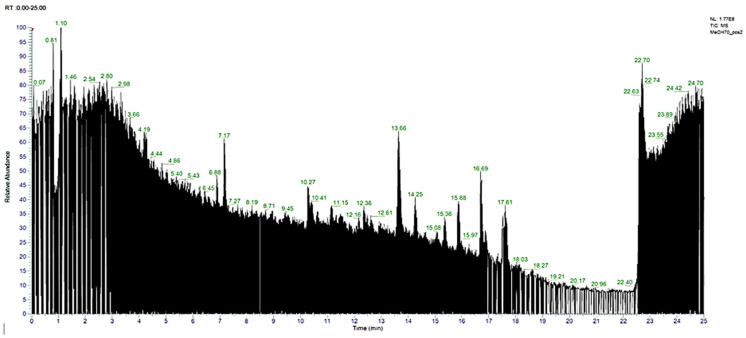
Total ion chromatogram blank.

**Figure 2:**
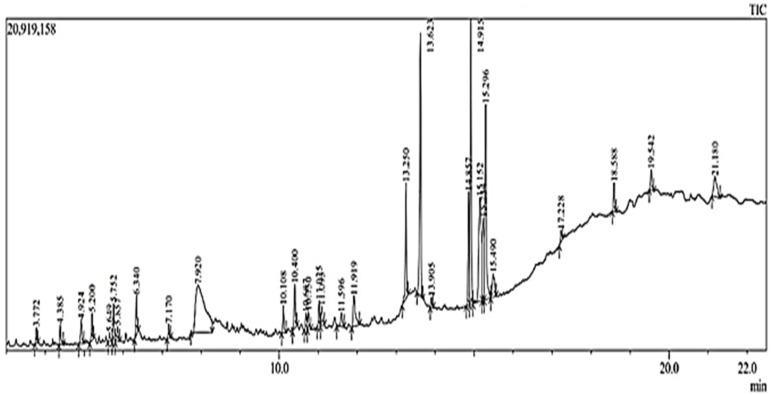
Total ion chromatogram of ethanol extract of *Toona sinensis* leaves.

The results obtained from the UHPLC–HRMS instrument are in the form of a chromatogram. Each chromatogram peak indicates the presence of one compound. The chromatogram is then processed using the Compound Discovery 3 application so that the *m*/*z* spectra can be identified; thus, the molecular formula of the interpreted compound can be predicted. The predicted molecular formula is then searched for the name of the compound with the help of the mzcloud database and the website https://www.chemspider.com/ (accessed on 1 July 2023). After obtaining the name of the compound and its structure through the mzcloud database and website, the measured *m*/*z* is compared with the calculated *m*/*z* by drawing the structure of the compound in question in the ChempDraw application. Figure 1 is the total ion chromatogram of the blank and the total ion chromatogram of the ethyl acetate fraction of Surian leaves on Figure 2. Based on the results of the interpretation of the chromatogram obtained for each peak, the predicted data for the Surian leaf Ethyl acetate fraction compound seen from mzCloud Best Match was more than 80%; 82 compounds were obtained, and 4 compounds based in the literature had the potential to act as an antiaging agent, as shown in Tables S1 and S2. Five compounds that have the potential to act as antiaging agents are Quercetin (5.78%), Quercetin-3β-d-glucoside (2.78%), Kaempferol (2.5%), and ellagic acid (0.43%).

**Figure 3:**
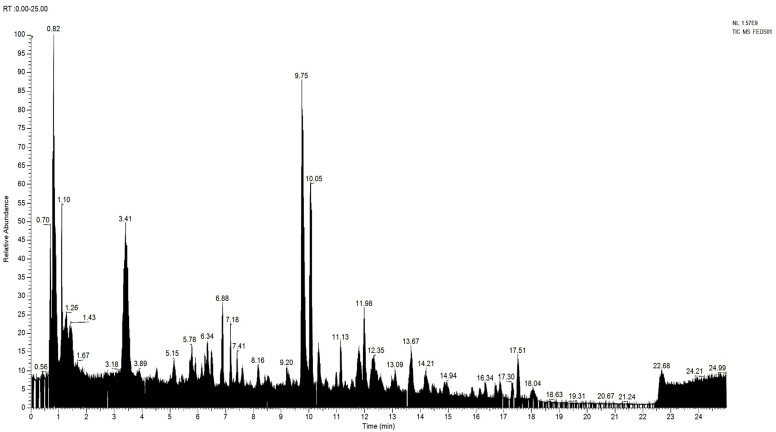
Total ion chromatogram of ethyl acetate fraction of *Toona sinensis* leaves.

Metabolite profiling aims to determine the compound content in ethanol extract and ethyl acetate fraction of Surian leaves (*Toona sinensis*). Metabolite profiling had been analyzed using UHPLC–HRMS instruments. This technique not only provides fast chromatography, better separation, and short chromatography run times but also high sensitivity and selectivity, accurate measurements, and reliable fragmentation, which ultimately help to elucidate the structure of various compounds [38,39]. The results of metabolite profiling using UHPLC–HRMS on the ethanol extract and ethyl acetate fraction of *Toona sinensis* shows that the ethanol extract and ethyl acetate fraction contain flavonoids as the main compound. Apart from that, there are other compounds such as Quercetin, Quercetin-3β-D-glucoside, Kaempferol, galic acid, and ellagic acid, which is a flavonoid compound and has the potential to act as an antiaging agent [20,30,32,33,34]. Flavonoid compounds have been reported to have antioxidant activity that works synergistically in stabilizing the role of reactive oxygen species (ROS) in the photoaging process [3]. Figure 2 shows the total ion chromatogram of the ethanol extract of *Toona sinensis* leaves, and Figure 3 shows the total ion chromatogram of ethyl acetate fraction of *Toona sinensis*.

Figure 2 shows the UHPLC–HRMS spectrum of ethanol extract of Surian leaves that detected several compounds such as the following: Quercetin; Quercetin-3β-d-glucoside; Kaempferol; Ellagic acid; 2,2-Dimethylvaleric acid; 2,4-Dihydroxy-2,5-dimethyl-3(2*H*)-furan-3-o; Benzyloxybenzene; Bis(4-ethylbenzylidene)sorbitol; Thymine; Ethyl 3-hydroxy-3,6-dihydro-2*H*-pyridine-1-c; 4*H*-Pyran-4-one; 2,3-Dihydro-3,5-dihydroxy-6; Benzoic acid; 4-Vinylphenol; 2-Methoxy-4-vinylphenol; 1,2,3-Benzenetriol; γ-Glutamyl-(S)-allyl-l-cysteine; *N*-isospathulenol; α-Cadinol; 1,1,4,7-Tetramethyldecahydro-1*H*-cyclopropa; l-Phenylalanine; *N*-acetyl-; methyl ester; 2-Propenoic acid; 3-(4-methoxyphenyl)-; Tetradecanoic acid; 1,4-Azulenediol; 1,2,3,3a,4,5,6,8a-octahydro-; Hexadecanoic acid; methyl ester; *n*-Hexadecanoic acid; Hexadecanoic acid; ethyl ester; 12,15-Octadecadienoic acid; methyl ester; 9,12,15-Octadecatrienoic acid; methyl ester; Phytol; 10*E*,12*Z*-Octadecadienoic acid; 9,12,15-Octadecatrienoic acid, (*Z*,*Z*,*Z*); Octadecanoic acid, 9-Octadecenamide; (*Z*)-, Bis(2-ethylhexyl) phthalate; 2,2-Dimethyl-3-(3,7,16,20-tetramethyl-heneic; α-Methyltyrosine; *N*,*O*,*O*′-tris(tert-butyld); l-Phenylalanine; 2-(3,4-dihydroxyphenyl)-5,7-dihydroxy-3-{[(2*S*,3*R*,4*S*,5*S*)-3,4,5-trihydroxyoxan-2-yl]oxy}-4*H*-chromen-4-one; 8-Hydroxyquinoline; Methyl Picolinate; Afzelin; Juglalin; Bis(methylbenzyli dene)sorbitol; galic acid; Cetrimonium; (2*S*,3*R*,4*R*,5*S*,6*S*)-2-{[2-(3,4-dihydroxyphenyl)-5,7-dihydroxy-4-oxo-4*H*-chromen-3-yl]oxy}-3,5-dihydroxy-6-methyloxan-4-yl-3,4,5-trihydroxybenzoate; D-(+)-Proline; Quercetin-3β-d-glucoside; Bis(4-ethylbenzylidene)sorbitol; Linoleoyl Ethanolamide; 9*Z*,11*E*,13*E*-Octadecatrienoic Acid methyl ester; L-Glutamic acid; (2*S*,3*R*,4*S*,5*R*,6*R*)-5-hydroxy-6-(hydroxymethyl)-3,4-bis(3,4,5-trihydroxybenzoyloxy)oxan-2-yl-3,4,5-trihydroxybenzoate; Dodecyltrimethylammonium; etc.

Figure 3 shows the UHPLC–HRMS spectrum of the ethyl acetate fraction of Surian leaves that detected several compounds such as the following: L-Phenylalanine; 2-(3,4-dihydroxyphenyl)-5,7-dihydroxy-3-{[(2*S*,3*R*,4*S*,5*S*)-3,4,5-trihydroxyoxan-2-yl]oxy} -4H-chromen-4-one; 8-Hydroxyquinoline; Methyl Picolinate; Afzelin; Juglalin; Bis(methylbenzyli dene)sorbitol; galic acid; Cetrimonium; (2*S*,3*R*,4*R*,5*S*,6*S*)-2-{[2-(3,4-dihydroxyphenyl)-5,7-dihydroxy-4-oxo-4H-chromen-3-yl]oxy}-3,5-dihydroxy-6-methyloxan-4-yl 3,4,5-trihydroxybenzoate; D-(+)-Proline; Quercetin-3β-D-glucoside; Bis(4-ethylbenzylidene)sorbitol; Linoleoyl Ethanolamide; 9*Z*,11*E*,13*E*-Octadecatrienoic Acid methyl ester; Ellagic acid; L-Glutamic acid; (2*S*,3*R*,4*S*,5*R*,6*R*)-5-hydroxy-6-(hydroxymethyl)-3,4-bis(3,4,5-trihydroxybenzoyloxy)oxan-2-yl 3,4,5-trihydroxybenzoate; Dodecyltrimethylammonium; Kaempferol; Quercetin; Coumarin; 5-Hydroxymethyl-2-furaldehyde; 5-hydroxy-2-(4-hydroxyphenyl)-8,8-dimethyl-4*H*,8*H*-pyrano [3,2-g]chromen-4-one; etc.

Predictions of molecular formulas and structures for all compounds contained in the ethanol extract and ethyl acetate fraction of *Toona sinensis* leaves are available in the Supplementary Materials.

Surian (*Toona sinensis*) is one of the medicinal ingredients that has long been used in traditional medicine. In its development, it is hoped that other parts of the Surian plant, such as Surian leaves, can be used as raw materials for traditional medicine. Sample preparation began with preparing Surian leaf simplicia (*Toona sinensis*). The simplicia yield obtained a value of 53.36%. Then, extraction was performed using the maceration method to obtain an ethanol extract yield of 33.28%, and the yield of the ethyl acetate fraction from Surian leaves was 0.526% [3]. The extracted sample is then hydrolyzed to break the glycone and aglycone bonds, which are generally found in the form of glycosides in secondary metabolite compounds. Hydrolysis with a 2 N HCl catalyst accompanied by stirring for 1 h at room temperature is reversible, so it is necessary to neutralize it using NaHCO_3_ to stop further hydrolysis reactions, which might damage the target compound. The addition of NaHCO_3_ was stopped when pH 7 (neutral) was reached. The hydrolysis results were further separated through a partition process using ethyl acetate solvent to produce ethyl acetate fractions with a yield of 0.88%.

UPLC-HRMS analysis was performed on EAFSL even though the yield was low at 0.526%, because this technique is very sensitive and is able to detect and identify bioactive compounds even in very small concentrations. This analysis aims to reveal the profile of secondary metabolites contained in the fraction that have antiaging activity. EAFSL contains semi-polar compounds with high bioactivity potential that cannot be ignored. Therefore, analysis using UPLC-HRMS was carried out to identify relevant target compounds and explain the chemical composition that contributes to the bioactivity potential of Surian leaf extract.

### 2.3. Formulation of SLE and EAFSL Nanoparticles

The development of nanoparticles derived from natural products, such as ethanol extract from Surian leaves, in drug delivery systems is currently an innovative and promising trend in the fields of pharmacy and medicine [1]. This utilization of natural resources not only reduces dependence on synthetic chemicals but also harnesses the therapeutic potential of various active compounds present in the plant extract [2]. Nanoparticles created from plant extracts, like the ethanol extract from Surian leaves, offer several advantages, including high biocompatibility, potent antioxidant potential, synergistic antiaging activity, and the ability to interact with biological cells. Moreover, the biodegradable nature of some plant extracts allows for a reduction in harmful environmental impact [3]. The application of these nanoparticles in drug delivery systems opens up opportunities to enhance bioavailability, stability, and target delivery, thereby improving their efficacy. With the ongoing progress in this research field, it is expected that more efficient and safe formulations will be discovered, making the use of nanoparticles from natural products a promising option for the development of advanced and environmentally friendly drug therapies [1].

Based on the results of the SLE and EAFSL research, SLE has an IC_50_ of 12.351 ppm and EAFSL has an IC_50_ of 15.5865 ppm. Vitamin C, as the positive control, was measured at 7.805 ppm. The IC_50_ results for SLE and EAFSL are close to the positive control of Vitamin C, categorizing them as having very strong antioxidant properties because the IC_50_ is <50 ppm. This antioxidant activity works synergistically in stabilizing the role of reactive oxygen species (ROS) in the photoaging process. Thus, the ethanol extract and the active fraction of Surian leaves (*Toona sinensis*) can be developed in the nanoparticle form [4].

**Figure 4:**
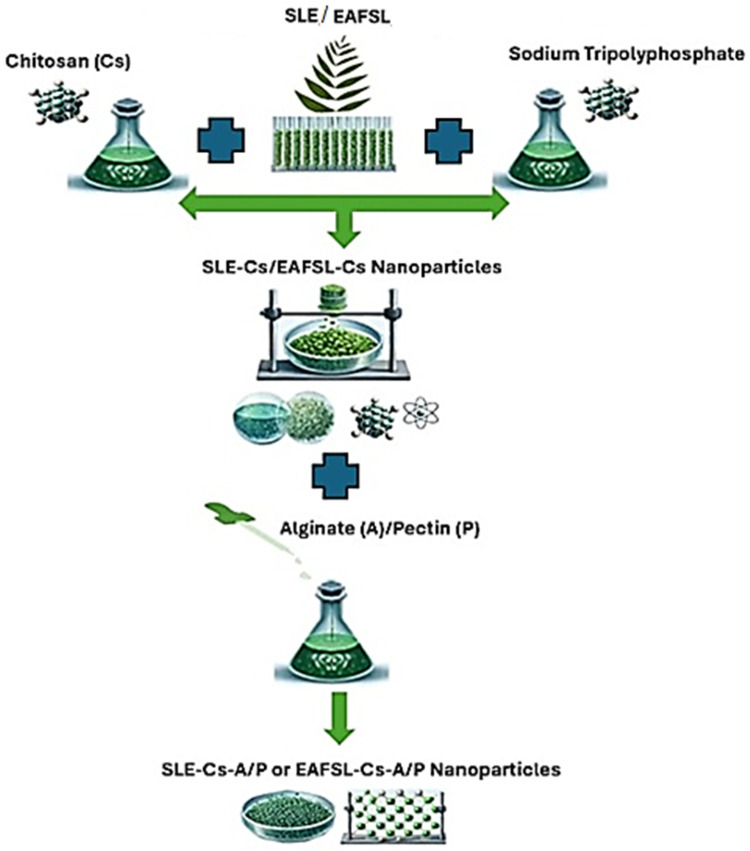
Ilustration of chitosan-based SLE and EAFSL nanoparticle formulation coated with alginate and pectin.

In this study, polymeric SLE and EAFSL nanoparticles, based on chitosan with sodium tripolyphosphate as the cross-linking agent, subsequently coated with alginate and pectin, are formulated to enhance antiaging activity. The formulation scheme for this research is illustrated in Figure 4.

The production of polymeric SLE and EAFSL nanoparticles was conducted using the ionic gelation method. Physical cross-linking through electrostatic interactions, as an alternative to chemical cross-linking, has been implemented to avoid potential toxicity from reactants and other undesired consequences. The mechanism of chitosan nanoparticle formation with this method is based on the positive charge of the amino groups (–NH_3_^+^) in chitosan interacting with the negative charge of the phosphate groups (PO_4_^3−^) from the cross-linking agent (i.e., sodium tripolyphosphate). Due to the complexation between these different charges, chitosan undergoes ionic gelation and successfully forms spherical particles. Thus, the nanoparticles are spontaneously formed due to mechanical stirring at room temperature [40,41,42].

Special conditions in the nanoparticle formulation in this study were carried out at a temperature of 40 °C with a stirring speed of 1000 rpm for 1 h (60 min). This condition is needed to ensure optimal particle size, stability, and high encapsulation efficiency. Other factors such as pH, mixing method, and type of polymer must be maintained properly to prevent aggregation and maintain a uniform and homogeneous particle size distribution. The special conditions above greatly affect the release of active substances, bioavailability, and interaction with target cells [41]. Therefore, the special conditions above are very important in adjusting the formulation parameters to suit the desired application such as antiaging cosmetics.

The mechanism of coating chitosan-based nanoparticles with alginate and pectin relies on the interaction between the amino groups on chitosan, functioning as positive charge donors (–NH_3_^+^), on the nanoparticle surface, and the carboxyl groups on alginate and pectin, serving as negative charge donors (COO^−^). This interaction facilitates the formation of cationic bonds between chitosan and alginate, as well as chitosan and pectin, providing stability and mechanical strength to the resulting nanoparticle structure [41]. A critical step in the surface coating of SLE and EAFSL nanoparticles using alginate and pectin (polyelectrolytes) is the inversion of the zeta potential of the nanoparticles. This inversion is crucial as agglomeration is likely to occur around the isoelectric point. The isoelectric point may influence the surface properties of the nanoparticles and can play a role in the nanoparticle agglomeration process. Agglomeration happens due to interactions between positively and negatively charged components on different nanoparticles or due to the absence of electrostatic stabilization during the intermediate stage of encapsulation. Optimization related to the pH values used is at pH 6.5 and pH 5 to maximize the positive charge of chitosan (amine group protonation) and the negative charge of alginate and pectin (carboxylate group deprotonation) [41,42].

The components of the polymeric nanoparticle system were selected based on the optimization that had been conducted. In summary, the ratios of SLE to chitosan (1:7, 1:5, 1:9), sodium tripolyphosphate to chitosan (1.5:1, 1:1, 2:1), chitosan to alginate (1:1, 1:2, 2:1), chitosan to pectin (1:2, 1:1, 5:1), EAFSL to chitosan (1:0.18, 1:4, 1:3), sodium tripolyphosphate to chitosan (1.5:1, 1:1, 1:1.5), chitosan to alginate (1:1.5, 1:2, 2:1), and chitosan to pectin (1.2:1, 1.3:1) can be seen in Table 1 and Table 2. The best formula will be chosen based on considerations of physicochemical properties and the characterization of the SLE and EAFSL nanoparticles produced.

### 2.4. Physicochemical Characteristics of SLE and EAFSL Nanoparticles

Physicochemical characteristics of SLE and EAFSL nanoparticles were assesed to understand and describe their properties, including organoleptic characteristics, pH, sedimentation degree, encapsulation efficiency, and surface morphology. The purpose of this characterization was to confirm the successful formation of nanoparticles and the encapsulation of SLE and EAFSL in the nanoparticle system.

In this study, the physicochemical characteristics of various SLE and EAFSL nanoparticle formulations using natural polymers, such as chitosan, alginate, and pectin with different concentrations, are presented in Table 3.

One-way ANOVA statistical analysis for pH, F, % EE, and % DL from the above results is stated as significant if *p* < 0.05, indicating a significant change between groups. The formula of the observation results where each formula of the extract nanoparticles and active fraction nanoparticles with chitosan–alginate and chitosan–pectin polymers has a significant difference, where different formulas with different polymers have significantly different pH values. Based on further testing, the pH value is still in the range of 2.49 to 3.28, but it is statistically stated to have a significant difference. The use of different polymers does not affect the pH range too much because it still meets the requirements. While for F, % EE, and % DL there is no significant change between groups, and the results do not have a significant difference.

**Figure 5:**
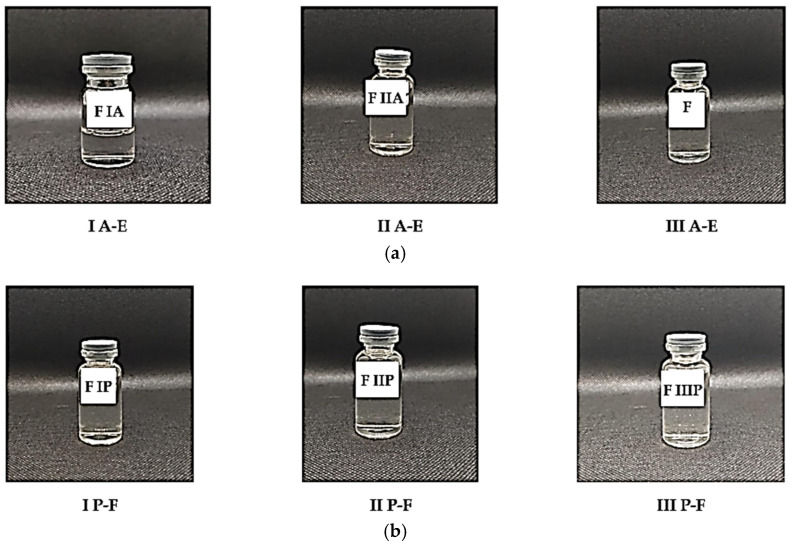
Formulation of SLE and EAFSL nanoparticles with chitosan–pectin polymers and chitosan and alginate polymers. Notes: (**a**) IA-E = SLE nanoparticles with 1% chitosan and 1% alginate; IIA-E = SLE nanoparticles with 0.75% chitosan and 1.25% alginate; IIIA-E = SLE nanoparticles with 1.25% chitosan and 0.75% alginate. (**b**) IP-F = EAFSL nanoparticles with 1% chitosan and 0.875% pectin; IIP-F = EAFSL nanoparticles with 0.75% chitosan and 0.625% pectin; IIIP-F = EAFSL nanoparticles with 1.25% chitosan and 0.375% pectin.

In this study, Figure 5 shows the image for the formulation of SLE and EAFSL nanoparticles with chitosan–alginate polymers and chitosan–pectin polymers. The physicochemical properties of SLE and EAFSL nanoparticles with chitosan–alginate and chitosan–pectin polymers exhibit a slightly yellowish color, acidic odor, acidic taste, clear solution form, and liquid consistency. The purpose of observing these organoleptic characteristics is to visually assess the sensory attributes of nanoparticles, such as color, odor, taste, form, and consistency. The acidic odor and taste are attributed to the acidic pH of SLE. The pH of SLE nanoparticles ranges from 3.03 to 3.41, while EAFSL falls within the pH range of 2.49–3.04. The clear solution form and liquid consistency indicate perfect solubility of the extracts in the carrier fluid. The slightly yellowish color is influenced by the dark brownish color of SLE and EAFSL. Since SLE and EAFSL dissolve completely in the carrier fluid, no precipitates or agglomerations of nanoparticles are found, resulting in a sedimentation degree value of 1.

The purpose of observing pH is to measure the acidity or alkalinity level of a solution, providing essential information related to the chemical balance and potential reactions within a system. Meanwhile, the objective of observing the sedimentation degree is to assess how quickly or slowly solid particles in a solution may settle, offering insights into the stability and consistency of a colloidal or suspension system [43].

**Figure 6:**
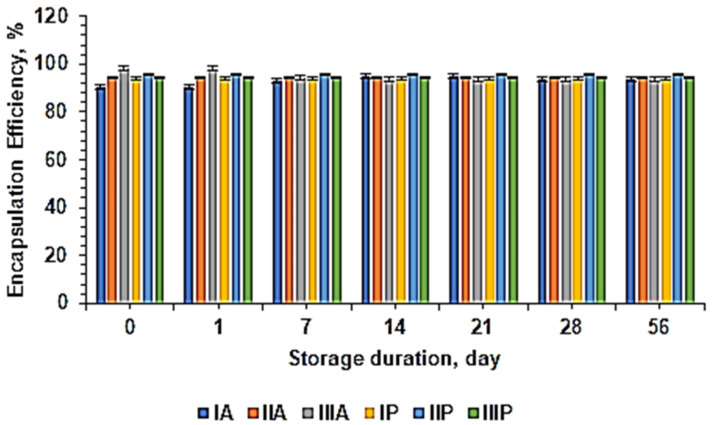
Stability of % EE of SLE nanoparticles with chitosan–alginate polymer and chitosan–pectin polymer.

Efficiency of encapsulation and drug loading are crucial parameters in nanoparticle formulation, as they influence the effectiveness of drug delivery and the administered dosage. These parameters are closely related to the ability of nanoparticles to efficiently transport and release drugs to the desired target site [44,45,46,47,48]. The purpose of determining the encapsulation efficiency and drug loading in this study is to understand the amount of SLE and EAFSL successfully encapsulated in the nanoparticle system. The method used for measuring encapsulation efficiency and drug loading is spectrophotometry, where measurements are conducted by assessing the sample absorbance and calculating the amount of SLE and EAFSL entrapped based on a previously established calibration curve. The encapsulation results can be seen in Figure 6. From the research results, the nanoparticle formulas SLE IA-E, IIA-E, and IIIA-E with chitosan and alginate polymers have % EE ranging from 97.86% to 98.46%, while the nanoparticle formulas EAFSL IA-F, IIA-F, and IIIA-F with chitosan and alginate polymers have % EE ranging from 98.30% to 98.74%, with each formula exhibiting high encapsulation efficiency values exceeding 80%. A high % EE is desirable as it indicates that a significant portion of SLE and EAFSL has been successfully encapsulated in the nanoparticles, enhancing the efficiency and safety of drug delivery to the desired target [44].

For the nanoparticle formulas SLE IP-E, IIP-E, and IIIP-E with chitosan and alginate polymers, % EE ranges from 97.80% to 98.13%, while the nanoparticle formulas EAFSL IP-F, IIP-F, and IIIP-F with chitosan and alginate polymers have % EE ranging from 98.30% to 98.90%, each formula maintaining high encapsulation efficiency values above 80%. It demonstrates that the encapsulation of EAFSL is higher than that of SLE, whether using chitosan–alginate or chitosan–pectin polymers.

Apart from that, the results of drug loading between SLE and EAFSL show no significant differences. This indicates that the surface coating of nanoparticles chitosan–pectin does not affect drug loading values. Drug loading values are not directly influenced by the polymer concentration in the formula but are influenced by the SLE and EAFSL content’s impact on the total weight of the components in the nanoparticle formula due to the increased mass of the polymer [40]. In this regard, EAFSL has larger drug loading than SLE, with % drug loading (DL) for EAFSL at 0.012% (120 ppm), while % drug loading (DL) for SLE is at 0.009% (90 ppm).

**Figure 7:**
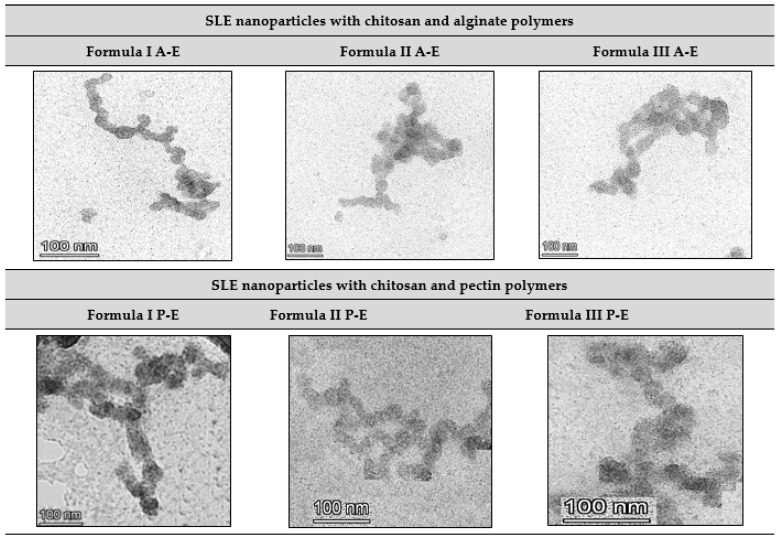
Surian leaves extract (SLE) nanoparticle forms. Notes: IA-E = SLE nanoparticles with 1% chitosan and 1% alginate; IIA-E = SLE nanoparticles with 0.75% chitosan and 1.25% alginate; IIIA-E = SLE nanoparticles with 1.25% chitosan and 0.75% alginate; IP-E = SLE nanoparticles with 1% chitosan and 0.5% pectin; IIP-E = SLE nanoparticles with 0.75% chitosan and 0.75% pectin; IIIP-E = SLE nanoparticles with 1.25% chitosan and 0.25% pectin.

The efficiency of encapsulation and drug loading in a formula affects the effectiveness of antiaging in drug delivery and the administered dosage. A higher % EE value implies a greater ratio of SLE and EAFSL within one particle, indicating a higher dose administered with a smaller number of nanoparticles. High drug loading values are also crucial for enhancing the formula’s effectiveness in drug delivery [41]. In addition to % EE, the physicochemical properties of nanoparticles can be observed through SLE and EAFSL nanoparticle forms, analyzed using transmission electron microscopy (TEM). Figure 7 shows Surian leaves extract (SLE) nanoparticle forms.

The shapes of SLE nanoparticles using chitosan–alginate and chitosan–pectin polymers were characterized by transmission electron microscopy (TEM). Transmission electron microscopy (TEM) is an advanced imaging technique utilizing an electron beam, as opposed to light, to examine the ultrastructure of materials at the nanoscale. In TEM, the sample is positioned in the path of an electron beam, and the transmitted electrons generate high-resolution images. Due to the shorter wavelength of electrons compared to visible light, TEM achieves significantly higher resolution than light microscopy.

In SLE nanoparticles using a polymer concentration where chitosan equals the amount of pectin and where the chitosan concentration is five times that of pectin, the shapes reveal the formation of nanoparticles. Pectin functions as a coating agent in forming a protective layer on the surface of nanoparticles, thus, forming a stable nanoparticle structure and protecting the active components of SLE and EAFSL in it. As a result, the active compound components of SLE and EAFSL are trapped in the nanoparticle matrix which makes their release slower and more controlled, but this strong attachment can stop SLE and EAFSL from interacting directly with the elastase enzyme which is an important part of the elastin decomposition process. So that it has an impact on the effectiveness of SLE and EAFSL in inhibiting the elastase enzyme decreasing, which results in the antiaging activity of this formulation being less effective because the formation of sediment is faster [33].

The utilization of chitosan, alginate, and pectin in nanoparticle fabrication significantly impacts the resulting surface morphology. Chitosan, a positively charged polymer derived from chitin, can interact with the negative charge of nanoparticles, producing an organized surface structure [45]. Alginate, derived from brown algae, imparts stability and strength to the nanoparticles, forming an even and structured layer on the surface. Meanwhile, pectin, a polysaccharide found in plant cell walls, provides mucoadhesive properties to the nanoparticles. The combination of these three elements creates a complex and controllable nanoparticle surface morphology system, opening up potential applications in drug delivery, food ingredients, and various other fields [46].

### 2.5. Characterization and Activity Elastase Enzyme of SLE and EAFSL Nanoparticle

Particle size, polydispersity index (PDI), and zeta potential are crucial for characterizing and understanding the properties of nanoparticles as a delivery system in antiaging applications [44]. The objective of this testing is to observe the influence of the ratio of chitosan–alginate and chitosan–pectin polymers on the characterization of particle size, polydispersity index (PDI), and zeta potential of nanoparticles. Particle size and polydispersity index measurements are conducted using a particle size analyzer (PSA) with the dynamic light scattering (DLS) method. DLS is a commonly used method for measuring particle size and polydispersity index in nanoparticle systems. This method assesses changes in light intensity produced or scattered by particles as they move randomly in a solution [47].

In this study, zeta potential was measured using the electrophoretic light scattering (ELC) method. This method combines the principles of electrophoresis with light scattering measurements to estimate the zeta potential of nanoparticles [48].

The table above reveals that SLE nanoparticles with the natural polymers (i.e., chitosan and alginate) exhibit the best characterization. They show the highest inhibitory activity against the elastase enzyme and possess the most favorable surface morphology compared to other formulations. Specifically, formula IIA-E, with a polymer concentration of 0.75% chitosan and 1.25% alginate, stands out with a particle size of 189.7 nm, a polydispersity index of 1.468, and a zeta potential of −20.0 mV. The inhibitory value of 1.4 mg/mL of SLE nanoparticles against the elastase enzyme is the highest, reaching 30.18%. The choice of characterizing SLE nanoparticles with chitosan and alginate polymer is based on the highest inhibition the against elastase enzyme, observed in formula IIA-E at 39.40 ± 1000, followed by formula IIIA-E at 18.20 ± 0.854.

From the bar graph above, it can be concluded that formula IIA-E of SLE nanoparticles (with twice the concentration of alginate compared to chitosan) has a greater inhibitory effect against the elastase enzyme compared to SLE alone.

Formula IA-E (SLE nanoparticles with 1% chitosan and 1% alginate) exhibits 0% inhibition. This can be attributed to two possibilities. Firstly, the polymer strongly binds with SLE, preventing its release, and secondly, the SLE nanoparticles are already dissolved in the water solvent, resulting in no antiaging activity.

The particle size in this study falls within the range of 100–200 nm, meeting the criteria for expected nanoparticle sizes, which typically range from 50 to 200 nm [49]. A drug compound demonstrates favorable characteristics when it is in the nanometer size range, enhancing dissolution rate, drug penetration, and bioavailability. This, in turn, prolongs the drug’s duration in systemic circulation and reduces the drug’s excretion rate, resulting in a more extended and effective impact [50].

Regarding the zeta potential values, as indicated by the obtained results, it is evident that the surface coating of nanoparticles with alginate and pectin leads to a surface charge inversion. This occurs due to the charged properties of alginate and pectin, as well as the electrostatic interactions between alginate and chitosan, and pectin and chitosan. Alginate is an anionic polysaccharide with negative charges on carboxylate groups (COO^−^), while chitosan is a cationic polysaccharide with positive charges on amine groups (NH_3_^+^). When alginate and pectin are applied to the surface of chitosan nanoparticles, the carboxylate groups on alginate and pectin interact with the amine groups on chitosan through ionic bonding or electrostatic interactions. This leads to the transfer of negative charges from alginate and pectin to the particle surface, resulting in negative zeta potential values. Additionally, since the molecules of alginate and pectin are primarily located in the outermost layer of the nanoparticles (enteric), the interaction between the negative charges of alginate and pectin and the positive charges of chitosan can induce strong electrostatic repulsion, yielding negative zeta potential values. A negative zeta potential in nanoparticles can lead to strong electrostatic repulsion. This prevents particle aggregation in a solution or biological media. The high dispersal stability of nanoparticles allows nanoparticles to stay separate from each other, maintaining the desired particle size and properties. This is crucial in drug delivery, as aggregated particles can reduce the efficiency of drug delivery to skin tissues. Nanoparticles with a negatively charged zeta potential tend to avoid adhesion to cellular surfaces or other biological components, enhancing stability, circulation in the body, and selective and efficient encapsulation, especially when coated with targeting ligands [51,52].

**Figure 8:**
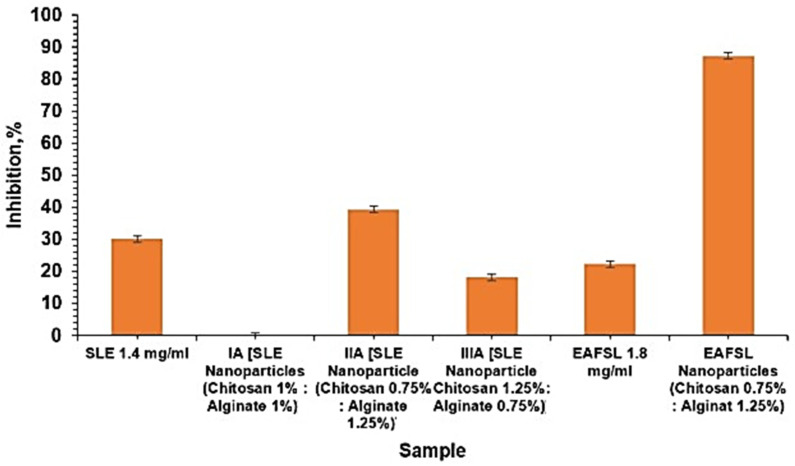
Comparison of elastase enzyme inhibition percentage between SLE and SLE nanoparticles and EAFSL and EAFSL nanoparticles with chitosan and alginate polymers.

From the table above, it is evident that EAFSL nanoparticles with natural polymers (i.e., chitosan and alginate) exhibit the best characterization. Among the various formulas, F IIA-F, with a polymer concentration of 0.75% chitosan and 1.25% alginate, shows the highest inhibitory activity against the elastase enzyme. The particle size is 205.0 nm, the polydispersity index is 1.975, and the zeta potential is −11.3 mV. The highest inhibitory value of EAFSL against the elastase enzyme is recorded at 22.42 ± 1.600. The selection of characterization for EAFSL nanoparticles with chitosan and alginate polymers is based on achieving the highest inhibition against elastase, notably in F IIA-F, which reaches 87.30 ± 1.000. Figure 8 shows comparison of elastase enzyme inhibition percentage between SLE and SLE nanoparticles and EAFSL and EAFSL nanoparticles with chitosan and alginate polymers.

The graph above indicates that the formula IIA-F of EAFSL nanoparticles with chitosan and alginate polymers has an inhibitory effect on elastase enzyme 3.6 times higher than the ethyl acetate fraction with a concentration of 1.8 mg/mL.

Regarding the influence of natural polymers on the inhibitory value against the elastase enzyme, the smaller the chitosan concentration is and the larger the alginate concentration is, the higher the percentage of inhibitory value against the elastase enzyme will be. This is because chitosan, alginates, and pectin play a crucial role in modulating the inhibitory activity against the elastase enzyme. Chitosan, being a positive polymer, can interact with the elastase enzyme electrostatically, forming complexes that inhibit the enzyme’s activity. Alginate, known for its ability to form a hydrogel, shields against the elastase enzyme and reduces its access to the substrate. Therefore, the use of chitosan and pectin influences the inhibitory activity against the elastase enzyme. The combined effects of chitosan and alginate create a synergistic inhibition system against elastase, demonstrating potential applications in the development of antiaging therapy for human skin care [53,54].

The zeta potential of EAFSL nanoparticles with chitosan and alginate polymers ranges from −11.3 mV to −20.4 mV. Thus, it may form larger aggregates due to their nanoparticle form. It is expected that in the nano-hydrogel preparation, the zeta potential will stabilize within the nano-hydrogel structure, as the hydrogel’s inherent property is to encapsulate formed nanoparticles. This encapsulation will integrate the nanoparticles into the nano-hydrogel structure.

**Figure 9:**
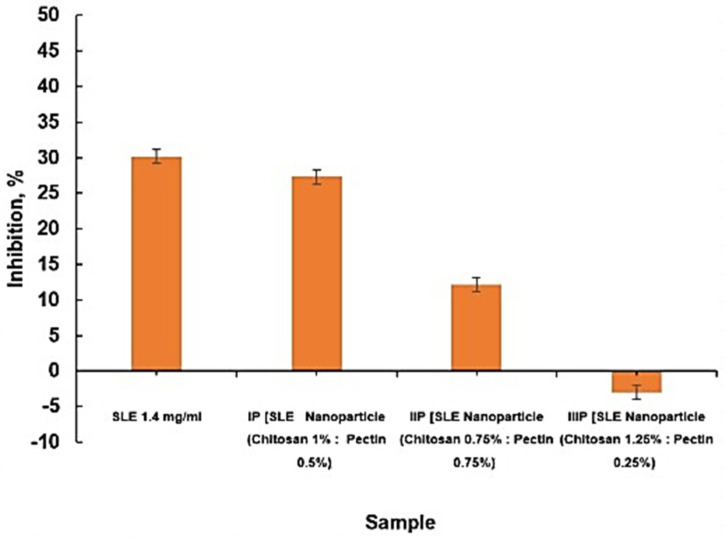
Comparison of elastase enzyme inhibition percentage between SLE and SLE nanoparticles with chitosan and pectin polymers.

From the table above, it can be observed that SLE nanoparticles with natural polymers (i.e., chitosan and pectin) exhibit the most favorable characteristics. Among the various formulations, F IP-E stands out with a chitosan concentration of 1% and a pectin concentration of 0.5%. This formulation demonstrates a particle size of 200.3 nm, a polydispersity index of 1.984, and a zeta potential of −23.6 mV. Additionally, it displays the highest inhibition value against elastase, reaching 30.18% at an SLE concentration of 1.4 mg/mL. The selection of this formulation for the characterization of SLE nanoparticles with chitosan and pectin polymers is based on its superior inhibitory activity against the elastase enzyme, registering at 27.28%, compared to other formulations. The comparison of elastase inhibition percentage between SLE and SLE nanoparticles with chitosan and pectin polymers can be seen in Figure 9.

From the graph above, it is evident that all formulations of SLE nanoparticles with chitosan and pectin polymers exhibit minimal inhibitory activity against the elastase enzyme. This observation can be attributed to the morphology of the nanoparticles, as revealed by TEM results, where a pectin membrane layer acts as an enteric coating around SLE nanoparticles. This coating makes it challenging for the active substance to exit, leading to inhibited activity.

Pectin, with its mucoadhesive properties, can retain elastase enzymes on the surface, hindering their interaction with substrates. The combined effects of chitosan, alginate, and pectin create a synergistic inhibition system against elastase, showcasing potential applications in developing anti-inflammatory therapies and treatments for conditions related to elastic tissue damage in humans [55].

Based on the results of the obtained polydispersity index, almost all formulations fall within the range of 1.3 to 1.9. This indicates that the majority of particles are highly polydisperse, as the polydispersity index values exceed >0.7, signifying a very wide distribution of particle sizes (non-uniform) and a likelihood of sedimentation, making them unstable. Nanoparticles with a uniform size distribution tend to be more stable in a solution medium. This reduces the possibility of particle aggregation or clumping, which can affect the properties and performance of nanoparticles. In drug delivery applications, a uniform particle size distribution can impact the efficiency of delivering the desired drug to the target. Particles with a uniform size have a better chance of penetrating tissues or overcoming biological barriers, thereby enhancing drug delivery efficiency [56].

The table above reveals that EAFSL nanoparticles containing natural polymers (i.e., chitosan and pectin) exhibit superior characteristics compared to other formulations, particularly F IP-F. This specific formulation, with a concentration of 1% chitosan and 0.875% pectin, stands out with a particle size of 175.5 nm, a polydispersity index of 1.713, and a zeta potential of −21.7 mV. The size of particles plays a pivotal role in drug delivery systems through the skin. Nano-sized particles are crucial for enhancing drug activity and penetration into skin tissues. In this context, the minute size of nanoparticles in nanometers facilitates more effective penetration into deeper layers of the skin. Nanoparticles with sizes in the nanometer range can traverse intercellular gaps and overcome structural barriers in skin tissues, such as the stratum corneum layer. Furthermore, the diminutive size of nanoparticles can improve drug solubility and facilitate absorption through the skin surface. Consequently, the incorporation of nanoparticles in topical drug formulations has the potential to enhance drug delivery efficiency, optimize drug penetration into skin tissues, improve the efficacy of topical therapy, and mitigate systemic impacts [57].

The utilization of chitosan, alginate, and pectin in nanoparticle formulations has a significant impact on particle size, polydispersity index, and zeta potential. Chitosan, acting as a positively charged polymer, contributes to the reduction in particle size through robust electrostatic interactions with the negative charge on nanoparticles. Alginate imparts stability and diminishes particle polydispersity, yielding a more homogeneous size distribution. Meanwhile, pectin can influence zeta potential by introducing surface charges that modulate interactions among particles. The synergy of these three components yields nanoparticles with the desired size, a narrow size distribution, and adjustable surface charge properties, providing enhanced control [58,59].

### 2.6. Stability Test at Room Temperature 

This stability test aims to ensure that nanoparticles remain stable during 2 months of storage, both in terms of particle size, distribution, and surface charge, and it ensures that nanoparticles do not experience aggregation or degradation that can affect the effectiveness and safety of their use during storage [34]. To ensure that the desired physicochemical characteristics remain in nanoparticles during 2 months of storage, this stability study is very necessary in the design of the methodology used so that changes in the physicochemical characteristics of nanoparticles can be identified, and a better formula design can be found during storage [34].

The short-term stability of SLE and EAFSL nanoparticles with chitosan–alginate and chitosan–pectin polymers after a two-month room temperature storage period can be observed in Table 4.

One-way ANOVA statistical analysis for particle size, PDI, zeta potential, % EE, pH, and F from the above results is stated as significant if *p* < 0.05, wherein there is a significant change between groups. The formula of the observation results where each formula of extract nanoparticles and active fraction nanoparticles with chitosan–alginate and chitosan–pectin polymers has a significant difference for before and after storage of SLE and EAFSL nanoparticles, where different formulas with different polymers have significantly different pH values. Based on further testing, the pH value is still in the desired range, but it is stated to have a statistically significant difference. The use of different polymers does not affect the pH range too much because it still meets the requirements. While for particle size, PDI, zeta potential, % EE, pH, and F there is no significant change between groups, and the results do not have a significant difference.

**Figure 10:**
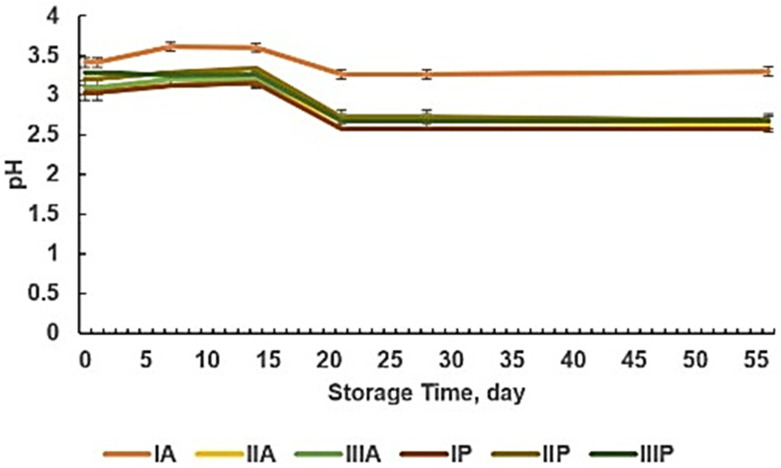
pH stability of SLE nanoparticles with chitosan–pectin polymer and chitosan–alginate polymer.

Short-term stability testing during two months of room temperature storage involves a series of procedures to ensure that a specific product or substance remains consistent and undergoes no significant changes during this storage period. Firstly, it is ensured that the storage environment meets the specified room temperature requirements. Following that, representative samples of the product or substance are taken, and their physical, chemical, and microbiological properties are observed at predetermined intervals. The analysis of these observations includes critical parameters that can influence the quality and safety of the product. If there are no significant changes in these parameters over two months, it is considered that the product or substance remains stable during room temperature storage. This short-term stability testing process is crucial to ensure that the product or substance continues to meet the expected quality standards throughout its shelf life [38]. The pH stability graph of SLE nanoparticles with chitosan–alginate and chitosan–pectin polymers during two-month storage at room temperature is presented in Figure 10.

Nanoparticles with positively charged polymers, such as chitosan, tend to be more stable at low pH (acidic), while nanoparticles with negatively charged polymers, like alginate and pectin, are more stable at high pH (alkaline). The pH stability of SLE nanoparticles with the chitosan polymer tends to increase during storage at low pH. Chitosan possesses weak acidic properties, indicating a positive charge at low pH. Therefore, nanoparticles made with chitosan carry a positive charge at low pH, preventing aggregation or clumping of positively charged nanoparticles and enhancing stability [59,60,61].

**Figure 11:**
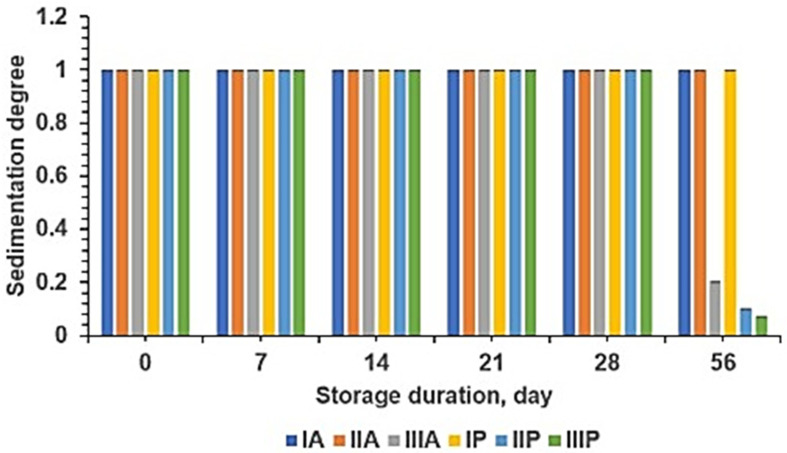
Sedimentation degree stability of SLE nanoparticles with chitosan–alginate polymer and chitosan–pectin polymer.

The sedimentation degree stability graph of SLE nanoparticles with chitosan–alginate and chitosan–pectin polymers during two-month storage at room temperature is presented in Figure 11.

Figure 11 shows the impact of chitosan and alginate polymers on the characterization and stability of SLE nanoparticles. As the concentration of chitosan decreases and the concentration of alginate increases, the characterization improves, and the nanoparticles become more stable, showing no signs of precipitation. Conversely, with an increase in chitosan concentration and a decrease in alginate concentration, the stability decreases, and precipitation occurs during storage. Among the SLE nanoparticle formulations with chitosan and alginate polymers, formulas IA and IIA prove to be stable during a two-month storage period at room temperature.

Figure 11 also presents the influence of chitosan and pectin polymers on the characterization and stability of SLE nanoparticles. When the concentration of chitosan is twice that of pectin, both the characterization and stability improve. However, when the concentrations of chitosan and pectin are equal, and the concentration of chitosan is five times that of pectin, the formation of a pectin coating (enteric) occurs on its surface, leading to faster precipitation during storage. Among the SLE nanoparticle formulations with chitosan and pectin polymers, formula IP proves to be stable during a two-month storage period at room temperature.

Furthermore, the stability of % EE in the SLE nanoparticle formula IIA-E with chitosan and alginate polymers maintains a stable % EE from initial production until the two-month storage at room temperature. In other words, the use of chitosan and alginate polymers does not affect % EE during storage. Figure 6 shows the stability of % EE of SLE nanoparticles with the chitosan–alginate polymer and chitosan–pectin polymer.

The stability of % EE in SLE nanoparticle formula IP-E with chitosan and pectin polymers remains consistent from the initial production to the two-month storage at room temperature. The use of chitosan and pectin polymers does not impact % EE during storage. Similarly, the utilization of chitosan, alginate, and pectin polymers does not affect % EE during storage, not only in formula IP-E but also in almost all formulations, maintaining stable % EE throughout the storage period.

## 3. Materials and Methods

### 3.1. Materials and Equipment

Materials used in this research were ethanol 70% (Brataco, Bandung, Indonesia), chitosan (Brataco), alginate (Brataco), pectin (Kisbiokim Medilab, Ahmedabad, India), NaTPP or sodium tripolyphosphate (Brataco), acetic acid 98% (Brataco), aluminum chloride (Brataco), sodium acetate (Brataco), methanol p.a. (Merck, Singapore), AQUA PRO Injection (Kimia Farma, Jakarta, Indonesia), and distilled water or Aquadest (Kisbiokim Medilab).

The equipment used in this research included a rotary evaporator (Heidolph, Schwabach, Germany), hot plate magnetic stirrer (Thermo Fisher Scientific, Singapore), particle size analyzer (Horiba Scientific SZ-100, Kyoto, Japan), UV-vis spectrophotometer (Horiba Scientific SZ-100, Kyoto, Japan), micro pipet 10–100 µL (Acura 825, Tokyo, Japan), pH meter (Hanna Instruments, Shenzhen, China), micropump (Hanna Instruments, Shenzhen, China), centrifuge (Labnet, Shenzhen, China), transmission electron microscopy (Tecnai G2 20 S-Twin Function, Shenzhen, China), ELISA microplates, and plasticware (Thermo Fisher Scientific, Waltham, MA, USA).

**Figure 12:**
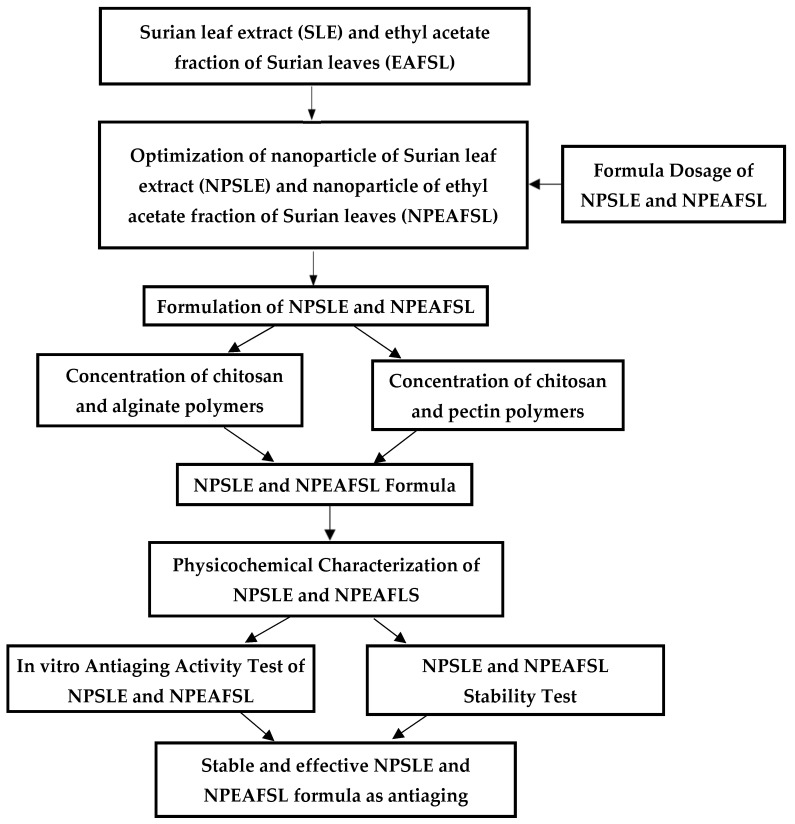
Flow chart of research process and methods.

The flow chart of research process and methods can be seen in Figure 12.

### 3.2. Collection of Plant Materials

Fresh leaves of Surian (*Toona sinensis*) were collected in the month of October 2022 from Rantau Suli Village, East Jangkat, Merangin Regency, Jambi Province, Indonesia, and they were identified by a taxonomist (Dr. Silva Abraham) from the Directorate of Scientific Collection Management of the National Research and Innovation Agency in Central Jakarta, where a voucher specimen (No. B-4601/II.6.2/DI.05.07/12/2022) was deposited. The fresh leaves were washed thoroughly to remove dirt and soil, then dried and stored at room temperature. These leaves were grinded and then kept in a closed container and stored at room temperature until use for the next process. Information about the plant, and the location and date of collection were stated in the Directorate of Scientific Collection Management of the National Research and Innovation Agency in Central Jakarta.

### 3.3. Extraction Process

Simplicia extraction of Surian leaves was conducted using the maceration method with ethanol 70% as the solvent. The process for extracting Surian leaves is outlined as follows: 5.98 kg of Surian leaf simplicia was weighed and placed into the macerator. Ethanol 70% was added until all Surian leaf simplicia were fully submerged, with a ratio of 1 part Surian leaf simplicia powder (5.98 kg) to 10 parts ethanol 70% solvent (60 L). The mixture was left to stand for 24 h. The obtained liquid extract was collected, resulting in 45 L of macerate. Subsequently, an equal amount of ethanol 70% was added back into the macerator. The extraction process was repeated for three cycles of 24 h each. The liquid extract obtained was then concentrated using a rotary evaporator at 50 °C. The extract was further concentrated using a water bath at 50 °C until a concentrated Surian leaf extract was obtained, and the extraction yield value was calculated [26].

### 3.4. Fractionation Process

The fractionation of Surian leaf extracts was conducted using the liquid–liquid extraction (LLE) method with three solvents of different polarities: n-hexane, ethyl acetate, and water. The fractionation process of Surian leaves is as follows: 50 g of Surian leaf extract was weighed and then ground with 400 mL of n-hexane in a mortar. The resulting filtrate was poured into a separating funnel until the n-hexane solvent was depleted. An equal amount of water was added, and the mixture was shaken for 15 min with occasional venting of air from the funnel every 5 min. The mixture in the separating funnel was allowed to stand until the two solvents separated thoroughly for 24 h. The n-hexane fraction was separated from the water fraction. The separation process was repeated until an almost colorless n-hexane fraction was obtained. An equal amount of ethyl acetate was added to the same separating funnel, shaken, and separated following the previous procedure. The n-hexane, ethyl acetate, and water fractions were concentrated using a rotary evaporator at 50 °C. The n-hexane, ethyl acetate, and water fractions were further concentrated using a water bath at 50 °C to obtain the concentrated n-hexane, ethyl acetate, and water fractions of Surian leaves [26].

### 3.5. Analysis of Metabolite Profiling with UHPLC–HRMS

#### 3.5.1. Hydrolysis and Partitioning Ethanol Extract *Toona sinensis* Leaf

After adding 2N HCl (1:2) to 5 g of *Toona sinensis* leaf ethanol extract, it was hydrolyzed and homogenized for one hour at room temperature using a magnetic stirrer hot plate. Next, sodium bicarbonate (NaHCO_3_) was added into the mixture until the pH became neutral. By adding 25 mm of ethyl acetate solvent to the bucket that had been separated and already holding the hydrolyzed concentrated extract, the hydrolyzed extract was divided. After that, it was combined for 15 min and left to settle until two layers—the water phase on the bottom and the organic phase, or ethyl acetate, on top—form. Until it lost its color, the researcher repeated the partitioning process with the same solvent.

#### 3.5.2. Sample Preparation for Analysis UHPLC–HRMS

Determination of the metabolite types of ethanol and ethyl acetate extracts of *Toona sinensis* leaf used the UHPLC–HRMS instrument with three replications. Carefully weighed was 10.00 mg of ethanol extract or the ethyl acetate fraction of *Toona sinensis* leaves, which was then dissolved in methanol into a 10 mL volumetric flask and added with a microsyringe as much as 3 µL. The mobile phases used were MS-grade water containing 0.1% formic acid (A) and MS-grade methanol containing 0.1% formic acid (B), employing a gradient technique with a flow rate of 0.3 mL/min. First, the mobile phase B was set at 5% and increased gradually to 90% in 16 min. Then, it was held at 90% for 4 min and continued to the initial condition (5% B) until 25 min. Data were obtained in the form of a chromatogram processed using the Compound Discovery 3 application, so the data are in the form of peak area and *m*/*z* spectra of each detected peak and the mzCloud database and the website https://www.chemspider.com/ (accessed on 1 July 2023).

### 3.6. Formulation of Surian Leaf Extract (SLE) Nanoparticles and Ethyl Acetate Fraction of Surian Leaves (EAFSL) Nanoparticles

The preparations of Surian leaf extract (SLE) nanoparticles and ethyl acetate fraction of Surian leaves (EAFSL) nanoparticles were carried out using the ionic gelation method with three formulations utilizing chitosan and alginate polymers and, additionally, three formulations using chitosan and pectin polymers. The SLE concentration was 1.4 mg/mL (1400 ppm), while EAFSL had a concentration of 1.8 mg/mL (1800 ppm), both utilizing NaTPP as a cross-linker. Concentrations of SLE 1.4 mg/mL and EAFSL 1.8 mg/mL were chosen because the % activity values against the elastase enzyme were 69.82% and 77.58% and the % inhibition (IC_50_) values against the elastase enzyme were 30.18% and 22.42%; these were based on in vitro evaluation of the Surian leaf extract against the elastase enzyme.

The formulations for SLE and EAFSL nanoparticles are presented in Table 1 and Table 2.

Table 1 shows the comparison ratio of the use of chitosan and alginate polymers and chitosan and pectin in the manufacturing of Surian Leaf Ethanol Extract Nanoparticles (NPSLE) for chitosan–alginate in the FIA-E formula (1:1), FIIA-E (3:5), and FIIIA-E (5:3), while for chitosan–pectin in the FIP-E formula (2:1), FIIP-E (1:1), and FIIIP-E (5:1). Special conditions in the nanoparticle formulation in this study were carried out at a temperature of 40 °C with a stirring speed of 1000 rpm for 1 h (60 min). These special conditions are needed to ensure that the particle size obtained is optimal, stable, and has high encapsulation efficiency [27].

Table 2 shows the comparison ratio of the use of chitosan and alginate polymers and chitosan and pectin in preparation of Surian Leaf Ethyl Acetate Fraction Nanoparticles (NPEAFSL) for chitosan–alginate in the formula FIA-F (2:3), FIIA-F (3:5), FIIIA-F (1:2), while for chitosan–pectin in the formula FIP-F (4:3.5), FIIP-F (3:2.5), FIIIP-F (2:1.5).

### 3.7. Nanoencapsulation of Plant Extracts

The preparation of SLE and EAFSL nanoparticles began by dissolving SLE in ethanol solvent with a concentration of 28 mg/20 mL (SLE was weighed at 28 mg and added to 20 mL of ethanol, and the mixture was homogenized) for each respective formula. For the preparation of EAFSL nanoparticles, EAFSL was dissolved in ethanol solvent with a concentration of 36 mg/20 mL (EAFSL was weighed at 36 mg and added to 20 mL of ethanol, and the mixture was homogenized) for each respective formula. Chitosan was weighed according to the concentration specified in the above formulas and dissolved in acetic acid with a total volume of 200 mL. Chitosan was added gradually to the acetic acid 2% solution and stirred with a magnetic stirrer until chitosan was completely dissolved. The pH was checked until it reached pH 4. In addition, alginate was weighed according to the concentration specified in the formulas F IA-E, F IIA-E, F IIIA-E, F IA-F, F IIA-F, and F IIIA-F. It was then dissolved in 50 mL of distilled water (Aquadest) and stirred until homogeneous. The pH was checked until it reached 6.5. Furthermore, pectin was weighed according to the concentration in the formulas F IP-E, F IIP-E, F IIIP-E, F IP-F, F IIP-F, and F IIIP-F. It was dissolved in 50 mL of distilled water (Aquadest), stirred until homogeneous, and the pH was checked until it reached 5. Additionally, NaTPP was weighed at 28 mg, added to 40 mL of distilled water (Aquadest) for each formula mentioned above, stirred homogeneously, and the pH was checked until it reached 3.6 [28].

The dissolved SLE and EAFSL were mixed with chitosan (mass 1). A micropump device consisting of 2 magnetic stirrers was prepared. On the left side, there was a solution of SLE, EAFSL, and chitosan (mass 1), and on the right side, there was a solution of NaTPP (mass 2). The principle of the micropump was that mass 1 would pass through the micropump drop by drop into mass 2 until the mass 1 solution was completely transferred to mass 2. Once mass 1 was depleted, the micropump was turned off. Mass 3 (SLE, EAFSL, and chitosan added to NaTPP) was transferred to another magnetic stirrer, and then alginate and pectin were added according to the above formulas, drop by drop, at a speed of 1000 RPM for 1 h at 40 °C [6].

### 3.8. Physicochemical Characterization of SLE and EAFSL Nanoparticles

Physicochemical characterization of nanoparticles was conducted to ensure the quality of the produced nanoparticles. The quality was observed through particle size, polydispersity index (PDI), zeta potential value, and the percentage of encapsulation efficiency (% EE) [24]. Particle size and PDI examinations were carried out using the particle size analyzer (PSA) with the dynamic light scattering (DLS) method. Furthermore, the zeta potential was determined using the Zetasizer Zen3600 with a 10-fold sample dilution in an aqueous medium at room temperature [28].

The examination of nanoparticle morphology was conducted using negative staining transmission electron microscopy (TEM). In summary, a drop of the sample, diluted with water to approximately 0.05 mg/mL, was placed on a Formvar copper mesh 200. It was allowed to adsorb, and the excess was removed using filter paper. A drop of uranyl acetate 2% solution (*w*/*v*) was added and allowed to contact the sample for 5 min. Excess water was removed, and the sample was air-dried before vesicles were observed using TEM operating at 200 KV [30].

For the % EE (encapsulation efficiency percentage) of SLE nanoparticles, 28 mg/20 mL of samples were centrifuged at 12,000 rpm for 60 min at 40 °C. The concentration of the free drug (in the supernatant, 1.5 mL) was determined by measuring the quercetin content in the supernatant using a UV-vis spectrophotometer at a previously specified maximum wavelength (i.e., 426 nm) [11].

$$\%\;EE=\frac{Total\;flavonoid\;content\;in\;SLE\;-\;Total\;flavonoid\;content\;in\;supernatant}{Total\;flavonoid\;content\;in\;SLE}\;\times \;100\%$$

The physical characteristics of SLE and EAFSL nanoparticles were examined, including organoleptic characteristics, pH, sedimentation degree, and chemical characteristics including % EE and % Drug Loading (DL). The observation of organoleptic properties aims to evaluate the sensory characteristics of nanoparticles, such as color, odor, taste, shape, and consistency observed visually. This is intended to ensure the quality of nanoparticles and assist in the development and improvement of nanoparticle formulations [31].

### 3.9. Short-Term Stability Test at Room Temperature for 2 Months

The objective of the short-term stability test at room temperature is to assess how well nanoparticles can preserve their quality during storage. Parameters monitored during room temperature storage include % EE (encapsulation efficiency), pH, sedimentation degree, and characterization throughout the storage period [32]. Accelerated stability testing was carried out over a period of 3 cycles (6 months), so that in this study, stability testing was carried out at room temperature for a period of 1 cycle for 2 months. The method of accelerated stability testing is that nanoparticles were placed in a room protected from exposure to sunlight with a room temperature of ±25 °C, observing each treatment such as pH, sedimentation rate, % EE, and % active substance on days 0, 1, 7, 14, 21, and 28 and at 2 months, while the characterization of SLE and EAFSL nanoparticles was observed before and after storage at room temperature for 2 months, such as testing particle size, polydispersity index, zeta potential, and % EE [34].

### 3.10. Activity Elastase Enzyme of SLE and EAFSL

The activity of the elastase enzyme was assessed using the neutrophil elastase inhibitor screening kit method. The process began by preparing the solution as follows: LE with a concentration of 1.4 mg/mL was dissolved in DMSO solvent. It was then diluted up to four times the desired final test concentration with assay buffer. Subsequently, 25 microliters of each diluted test compound was added to separate wells of a 96-well plate [20]. The various SLE nanoparticle formulas that had been prepared were also included [33].

Inhibitor control stock 1:25 was diluted with assay buffer, and 25 microliters of the diluted inhibitor control was added to separate wells of the plate. An additional 25 microliters of assay buffer was added to separate the wells on the plate. It should be noted that enzyme controls needed to be set each time the test was conducted. Finally, 75 microliters of assay buffer was added to separate the wells on the plate. For each well (except the background control well), 50 microliters of neutrophil elastase solution were prepared according to Table 5.

A total of 50 microliters of diluted neutrophil elastase solution was added to each well labeled as test compound, inhibitor control, and enzyme control. The solution was not added to background control wells. The plate was mixed thoroughly and incubated for 5 min at 37 °C. The plate was protected from light during incubation. The volume in all wells—including test compounds, inhibitor control, enzyme control, and background control at this step—was 75 microliters [33]. A sufficient amount of reagent for the intended number of tests was prepared. A total of 25 microliters of the reaction mixture were prepared for each well according to Table 6.

A total of 25 microliters of the reaction mixture was added to each reaction container—including test compound, inhibitor control, enzyme control, and background control. The plate was mixed, and measurements were taken immediately [33].

Fluorescence (relative fluorescence unit [RFU]) was measured at λ_Ex_ = 400 nm/λ_Em_ = 505 nm in the microplate. It was read in kinetic mode for 30 min at 37 °C. The plate was protected from light during incubation. It was recommended to read fluorescence at every 10-minute interval: 0, 10, 20, and 30 min. After obtaining the encapsulation values, calculations were performed for % elastase enzyme activity against the test compound and % elastase enzyme inhibition against the test compound using the following formulas:

$$\%\;Activity\;=\;\frac{\Delta \;RFU\;Test\;Compound}{\Delta \;RFU\;Enzyme\;Control}\;\times \;100\%$$

$$\%\;Inhibition\;=\;1\;-\;\left(\frac{\Delta \;RFU\;Test\;Compound}{\Delta \;RFU\;Enzyme\;Control}\right)\;\times \;100\%$$

Then, a graph depicting the profile of neutrophil elastase (NE) activity was generated with different concentrations of the test compound. Additionally, a graph illustrating the relationship between % activity values and % inhibition values of the test compound was also created.

### 3.11. Data Analysis

The experimental results included three replications, and the data were expressed as mean ± standard deviation (SD). The data were analyzed by an ANOVA (*p* < 0.05) using SPSS (version SPSS Statistics 29.0.x), and *p* < 0.05 was considered to be statistically significant.

## 4. Conclusions

The use of natural polymers (chitosan, alginate, and pectin) in nanoparticle formulations affects the antiaging activity of the elastase enzyme. Natural polymers function as sheathing agents in forming a protective layer on the surface of nanoparticles, thus, forming a stable structure and protecting active components such as SLE and EAFSL in them. The active compound components of SLE and EAFSL are trapped in the nanoparticle matrix, which will make their release slower and more controlled. The use of chitosan–alginate polymers, such as FIIA-NPEAFSL, result in higher inhibition (IC_50_) against the elastase enzyme than FIIA-NPSLE, where the % inhibition (IC_50_) of FIIA-NPEAFSL is 87.30%, while the IC_50_ of FIIA-NPSLE is 39.40%. The use of chitosan–pectin polymers, such as FIP-NPSLE, results in lower inhibition of the elastase enzyme compared to chitosan–alginate polymers, namely IC_50_ of 27.28%, while the IC_50_ for FIIIP-NPEAFSL was 39.53%. The characterization of SLE and EAFSL nanoparticles with chitosan–alginate and chitosan–pectin polymers resulted in significant polydispersity index (PDI) during storage from 1.3 to 1.9. As a consequence, the generated zeta potential values were very low, ranging from −11 mV to −27 mV, increasing the likelihood of aggregate formation during storage; this is due to the ionic gelation technique method used in the manufacture of nanoparticles. The results of metabolite profiling using UHPLC–HRMS on *Toona sinensis* leaf extracts revealed that the main compounds were glycitein, quercetin, quercetin-3β-d-glucoside, kaempferol, and ellagic acid, which have the potential to act as antiaging agents. It can be concluded that the use of natural polymers (chitosan, alginate, and pectin) in the process of encapsulating the extract into nanoparticles with the same process variables greatly affect the evaluation of antiaging activity in elastase enzymes. Future research will be carried out on the development of SLE and EAFSL nanohydrogels with antiaging activity.

## Figures and Tables

**Table 1:** SLE nanoparticle formulations.

Ingredients	F IA-E	F IIA-E	F IIIA-E	F IP-E	F IIP-E	F IIIP-E	Functions
SLE (%)	0.14	0.14	0.14	0.14	0.14	0.14	Active substance
Chitosan (%)	1	0.75	1.25	1	0.75	1.25	Natural polymer (polycation)
Alginate (%)	1	1.25	0.75	-	-	-	Natural polymer (enteric)
Pectin (%)	-	-	-	0.5	0.75	0.25	Natural polymer (enteric)
NaTPP (%)	0.7	0.7	0.7	0.7	0.7	0.7	Cross-linker (polyanion)
Acetic acid + distilled water (mL) ad	310	310	310	310	310	310	Solvent

**Table 2:** EAFSL nanoparticle formulations.

Ingredients	F IA-F	F IIA-F	F IIIA-F	F IP-F	F IIP-F	F IIIP-F	Functions
EAFSL (%)	0.18	0.18	0.18	0.18	0.18	0.18	Active substance
Chitosan (%)	1	0.75	0.5	1	0.75	0.5	Natural polymer (polycation)
Alginate (%)	1.5	1.25	1	-	-	-	Natural polymer (enteric)
Pectin (%)	-	-	-	0.875	0.625	0.375	Natural polymer (enteric)
NaTPP (%)	0.7	0.7	0.7	0.7	0.7	0.7	Cross-linker (polyanion)
Acetic acid + distilled water (mL) ad	310	310	310	310	310	310	Solvent

**Table 3:** Physicochemical characteristics of SLE and EAFSL nanoparticles.

Formulas	Organoleptic Properties					pH	F	% EE	% DL
	Color	Odor	Taste	Form	Consistency				
**SLE Nanoparticles with Chitosan and Alginate**									
IA-E	slightly yellow	Acidic	acidic	clear solution	Liquid	3.41 ± 0.077	1 ± 0	97.86 ± 0.05	0.009 ± 0
IIA-E	slightly yellow	Acidic	acidic	clear solution	Liquid	3.03 ± 0.045	1 ± 0	98.13 ± 0.01	0.009 ± 0
IIIA-E	slightly yellow	Acidic	acidic	clear solution	Liquid	3.10 ± 0.020	1 ± 0	98.46 ± 0.05	0.009 ± 0
**SLE Nanoparticles with Chitosan and Pectin**									
IP-E	slightly yellow	Acidic	acidic	clear solution	Liquid	3.03 ± 0.055	1 ± 0	98.07 ± 0.01	0.009 ± 0
IIP-E	slightly yellow	Acidic	acidic	clear solution	Liquid	3.21 ± 0.015	1 ± 0	97.80 ± 0.01	0.009 ± 0
IIIP-E	slightly yellow	Acidic	acidic	clear solution	Liquid	3.28 ± 0.032	1 ± 0	98.13 ± 0.01	0.009 ± 0
**EAFSL Nanoparticles with Chitosan and Alginate**									
IA-F	slightly yellow	Acidic	acidic	clear solution	Liquid	2.64 ± 0.0152	1 ± 00	98.30 ± 0.05	0.012 ± 0
IIA-F	slightly yellow	Acidic	acidic	clear solution	Liquid	3.04 ± 0.020	1 ± 0	98.50 ± 0.01	0.012 ± 0
IIIA-F	slightly yellow	Acidic	acidic	clear solution	Liquid	2.58 ± 0.0152	1 ± 0	98.74 ± 0.05	0.012 ± 0
**EAFSL Nanoparticles with Chitosan and Pectin**									
IP-F	slightly yellow	Acidic	acidic	clear solution	Liquid	2.56 ± 0.0251	1 ± 0	98.30 ± 0.01	0.012 ± 0
IIP-F	slightly yellow	Acidic	acidic	clear solution	Liquid	2.55 ± 0.0472	1 ± 0	98.50 ± 0.01	0.012 ± 0
IIIP-F	slightly yellow	Acidic	acidic	clear solution	Liquid	2.49 ± 0.0208	1 ± 0	98.90 ± 0.01	0.012 ± 0

Notes: IA-E = SLE nanoparticles with 1% chitosan and 1% alginate; IIA-E = SLE nanoparticles with 0.75% chitosan and 1.25% alginate; IIIA-E = SLE nanoparticles with 1.25% chitosan and 0.75% alginate; IP-E = SLE nanoparticles with 1% chitosan and 0.5% pectin; IIP-E = SLE nanoparticles with 0.75% chitosan and 0.75% pectin; IIIP-E = SLE nanoparticles with 1.25% chitosan and 0.25% pectin; IA-F = EAFSL nanoparticles with 1% chitosan and 1.5% alginate; IIA-F = EAFSL nanoparticles with 0.75% chitosan and 1.25% alginate; IIIA-F = EAFSL nanoparticles with 0.5% chitosan and 1% alginate; IP-F = EAFSL nanoparticles with 1% chitosan and 0.875% pectin; IIP-F = EAFSL nanoparticles with 0.75% chitosan and 0.625% pectin; IIIP-F = EAFSL nanoparticles with 1.25% chitosan and 0.375% pectin.

**Table 4:** Short-term stability before and after storage of SLE and EAFSL nanoparticles.

Formulas	Before Storage						After Storage					
	Particle Size (nm)	PDI	Zeta Potential (mV)	% EE	pH	F	Particle Size (nm)	PDI	Zeta Potential (mV)	% EE	pH	F
**SLE Nanoparticles**												
IA-E	173.0 ± 1.00	1.401 ± 0.01	−22.1 ± 0.06	97.86 ± 0.01	3.41 ± 0.07	1 ± 0.0	1113.3 ± 1.00	1.102 ± 0.01	−13.9 ± 0.01	93.82 ± 0.05	3.30 ± 0.04	1 ± 0.0
IIA-E	189.7 ± 1.63	1.468 ± 0.00	−20.0 ± 0.10	98.13 ± 0.05	3.03 ± 0.05	1 ± 0.0	977.4 ± 0.01	0.726 ± 0.05	−21.6 ± 0.03	94.06 ± 0.01	2.63 ± 0.01	1 ± 0.0
IIIA-E	187.6 ± 1.41	1.382 ± 0.01	−22.3 ± 0.10	98.46 ± 0.05	3.10 ± 0.02	1 ± 0.0	1214.4 ± 1.00	1.470 ± 1.00	−15.8 ± 0.00	93.52 ± 0.05	2.67 ± 0.01	0.2 ± 0.0
IP-E	200.3 ± 0.57	1.984 ± 1.00	−23.6 ± 0.69	98.07 ± 0.01	3.03 ± 0.06	1 ± 0.0	858.9 ± 1.00	0.660 ± 0.01	−23.7 ± 0.03	93.52 ± 0.01	2.58 ± 0.05	1 ± 0.0
IIP-E	200.7 ± 1.00	1.672 ± 0.01	−22.4 ± 1.00	97.80 ± 0.01	3.21 ± 0.02	1 ± 0.0	1096.2 ± 0.05	1.158 ± 0.05	−18.2 ± 0.01	95.68 ± 0.01	2.69 ± 0.01	0.1 ± 0
IIIP-E	193.4 ± 0.67	1.478 ± 0.00	−27.9 ± 0.01	98.13 ± 0.01	3.28 ± 0.03	1 ± 0.0	958.1 ± 1.00	0.787 ± 1.00	−20.1 ± 1.00	93.82 ± 0.01	2.67 ± 0.02	0.07 ± 0.0
**EAFSL Nanoparticles**												
IA-F	183.0 ± 0.50	1.781 ± 0.01	−20.4 ± 0.59	98.30 ± 0.05	2.64 ± 0.02	1 ± 0.0	617.0 ± 0.50	0.862 ± 1.00	−20.0 ± 1.00	96.62 ± 0.05	2.65 ± 0.01	0.01 ± 0.0
IIA-F	205.0 ± 1.00	1.975 ± 0.01	−11.3 ± 1.00	98.50 ± 0.05	3.04 ± 0.02	1 ± 0.0	460.5 ± 1.00	0.508 ± 1.00	−20.8 ± 0.01	96.50 ± 0.01	2.57 ± 0.03	1 ± 0.0
IIIA-F	172.9 ± 0.95	1.828 ± 0.00	−15.0 ± 1.00	98.74 ± 0.05	2.58 ± 0.02	1 ± 0.0	813.2 ± 1.00	0.872 ± 0.05	−21.6 ± 1.00	97.26 ± 0.05	2.59 ± 0.01	1 ± 0.0
IP-F	175.5 ± 1.00	1.713 ± 1.00	−21.7 ± 1.00	98.30 ± 0.01	2.56 ± 0.03	1 ± 0.0	627.4 ± 0.05	1.704 ± 1.00	−10.6 ± 0.03	96.10 ± 0.01	2.61 ± 0.01	1 ± 0.0
IIP-F	175.3 ± 0.01	1.887 ± 1.00	−23.2 ± 0.01	98.50 ± 0.01	2.55 ± 0.05	1 ± 0.0	671.4 ± 1.00	1.769 ± 0.05	−16.6 ± 1.00	96.82 ± 0.01	2.61 ± 0.02	0.2 ± 0.0
IIIP-F	176.1 ± 0.00	1.938 ± 0.01	−20.9 ± 0.00	98.90 ± 0.01	2.49 ± 0.02	1 ± 0.0	468.7 ± 1.00	0.491 ± 0.03	−18.1 ± 0.02	97.54 ± 0.01	2.52 ± 0.01	0.01 ± 0.0

Notes: IA-E = SLE nanoparticles with 1% chitosan and 1% alginate; IIA-E = SLE nanoparticles with 0.75% chitosan and 1.25% alginate; IIIA-E = SLE nanoparticles with 1.25% chitosan and 0.75% alginate; IP-E = SLE nanoparticles with 1% chitosan and 0.5% pectin; IIP-E = SLE nanoparticles with 0.75% chitosan and 0.75% pectin; IIIP-E = SLE nanoparticles with 1.25% chitosan and 0.25% pectin; IA-F = EAFSL nanoparticles with 1% chitosan and 1.5% alginate; IIA-F = EAFSL nanoparticles with 0.75% chitosan and 1.25% alginate; IIIA-F = EAFSL nanoparticles with 0.5% chitosan and 1% alginate; IP-F = EAFSL nanoparticles with 1% chitosan and 0.875% pectin; IIP-F = EAFSL nanoparticles with 0.75% chitosan and 0.625% pectin; IIIP-F = EAFSL nanoparticles with 1.25% chitosan and 0.375% pectin.

**Table 5:** Reagent volumes for neutrophil elastase solution preparation.

Reagent	Volume
Assay buffer	48 microliters
Neutrophil elastase stock solution	2 microliters

**Table 6:** Components and volumes of the reaction mixture for enzyme activity assessment.

Reagent	Working Reagent
Assay buffer	23 microliters
Substrate	2 microliters

## Data Availability

Data sharing does not apply to this study.

## Supplementary Materials

The following supporting information can be downloaded at: https://www.mdpi.com/article/10.3390/ph18030288/s1, Table S1: Predicted of Ethyl acetate Fraction Compounds in 1 Surian (*Toona sinensis*) Leaves; Table S2: Predicted of Ethanol Extract Compounds in Surian Leaves.
