# Supplementary material for: Formulation Development of Natural Polymeric Nanoparticles, In Vitro Antiaging Evaluation, and Metabolite Profiling of Toona sinensis Leaf Extracts
Source: Pharmaceuticals (Basel). 2025 Feb 20;18(3):288. doi: 10.3390/ph18030288 (PMC11945715; doi:10.3390/ph18030288)
Supplement: Supplementary file 1 [file pharmaceuticals-18-00288-s001.zip › pharmaceuticals-3417799-Table S1.pdf]

Table S1: Predicted of Ethyl acetate Fraction Compounds in 1 Surian (*Toona sinensis*) Leaves

No	Compound Name and Structure	Molecular Formula	Molecular Weight	Retention Time (min)	Area Max	Mz Clo Best M (%)	Concentratio (%)	Antiaging Activity	Chromatogram
1.	L-Phenylalanine 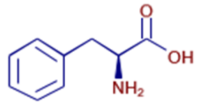	$C_9H_{11}NO_2$	166,0862	1,443	804161675,3	100	6,716087646	-	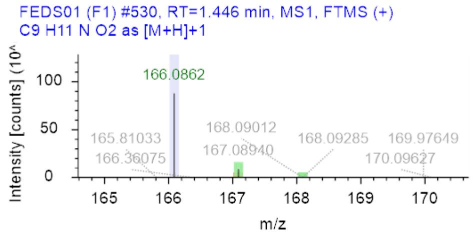 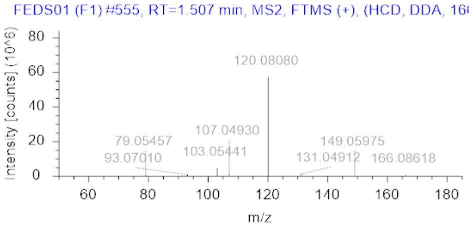
2.	2-(3,4-dihydroxyphenyl)-5,7-dihydroxy-3-{[(2S,3R,4S,5S)-3,4,5-trihydroxyoxan-2-yl]oxy}-4H-chromen-4-one 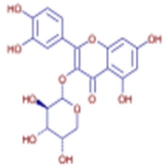	$C_{20}H_{18}O_{11}$	435,09137	6,15	104649285,9	99,9	0,873995613	-	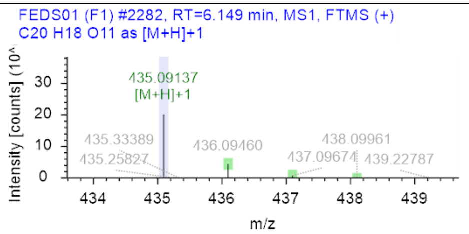 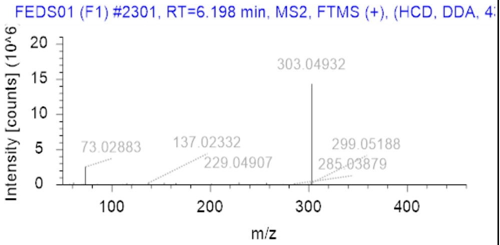

3.	8-Hydroxyquinoline<br>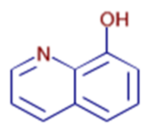 	C 9 H 7 N O	146,05998	3,313	11306407,24	99,9	0,094427308	-	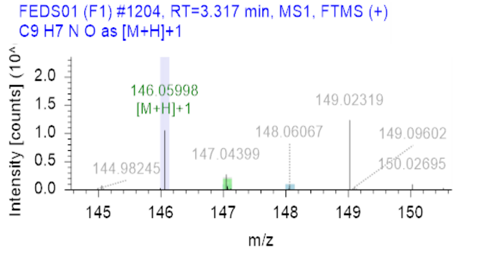<br><br>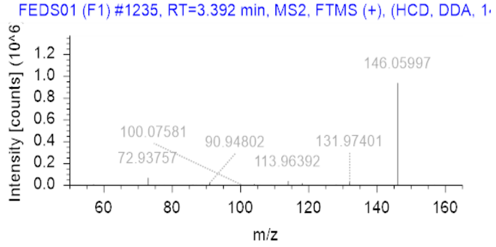
4	Methyl Picolinate<br>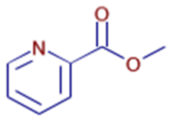 	C 7H 7N O2	138,05486	3,89	148471735,9	99,8	1,239985966	-	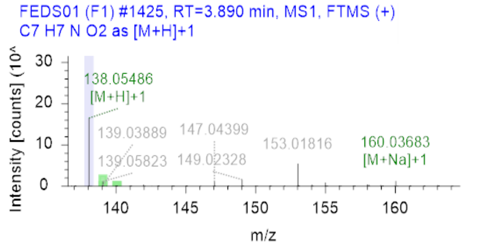<br><br>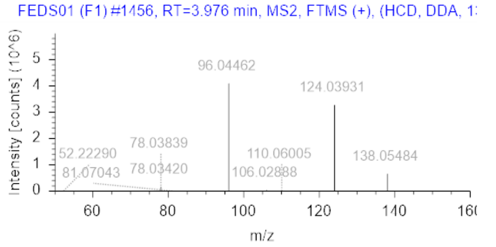

5	Afzelin 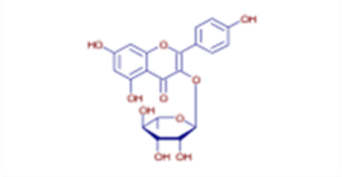	$C_{21}H_{20}O_{10}$	433,11200	6,887	135326679,9	99,8	1,130202882	-	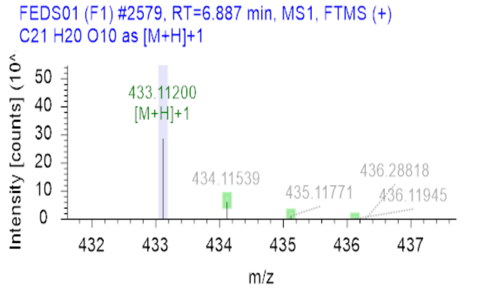 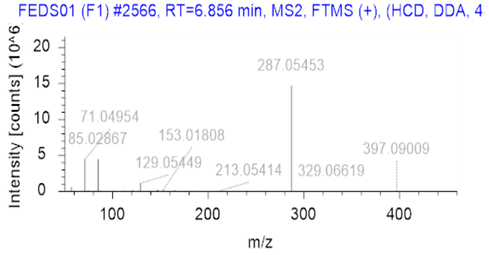
6	Juglalin 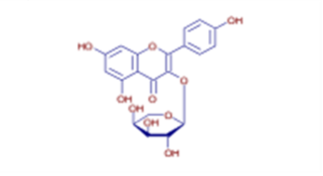	$C_{20}H_{18}O_{10}$	419,09671	6,538	80588820,81	99,8	0,673050707	-	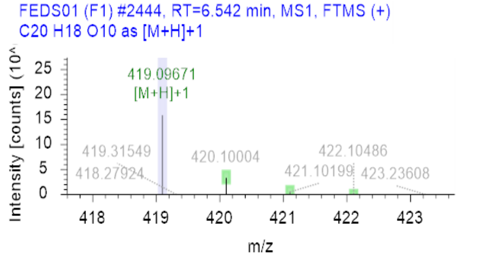 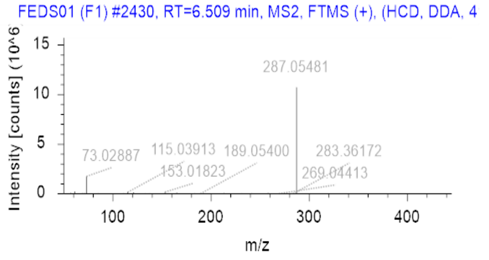

7	Bis(methylbenzylidene)sorbitol 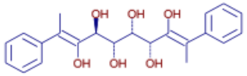	$C_{22}H_{26}O_6$	387,17966	10,618	75100510,31	99,7	0,627214185	-	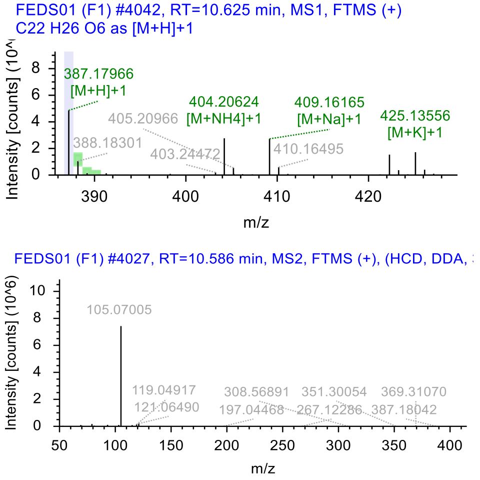
8	Cetrimonium 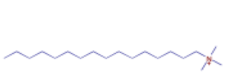	$C_{19}H_{41}N$	284,33118	12,808	34757083,46	99,7	0,290279462	-	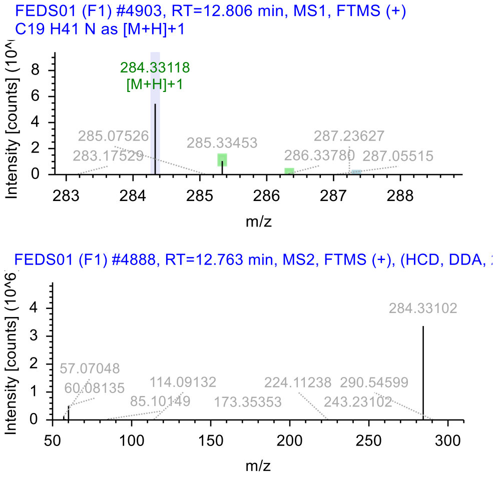
9	(2S,3R,4R,5S,6S)-2-{[2-(3,4-dihydroxyphenyl)-5,7-dihydroxy-4-oxo-4H-chromen-3-yl]oxy}-3,5-dihydroxy-6-methyl	$C_{28}H_{24}O_{15}$	601,11755	7,382	17004753,58	99,7	0,142017978	-	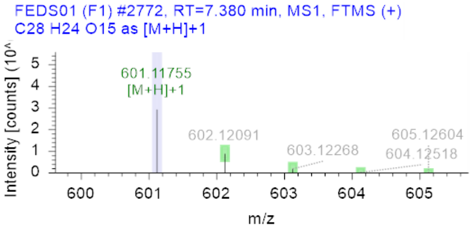

	oxan-4-yl 3,4,5-tri hydroxybenzoate 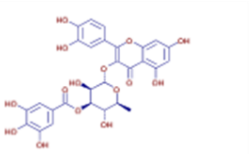								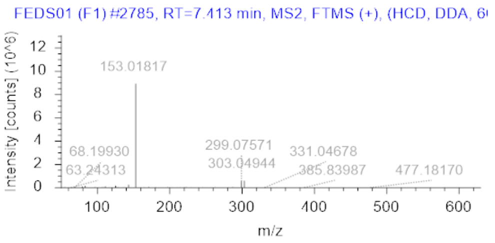
10	D-(+)-Proline 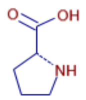	$C_5H_9NO_2$	116,07074	0,812	673738009,6	99,6	5,626833088	-	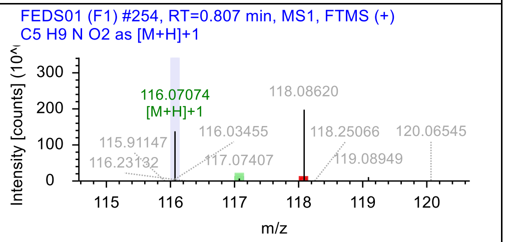 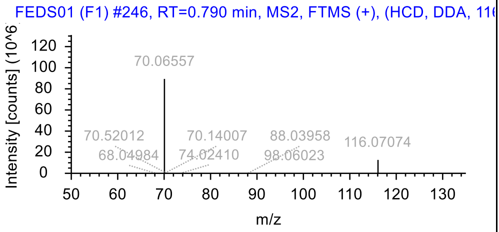

11	Quercetin-3β-D-glucoside 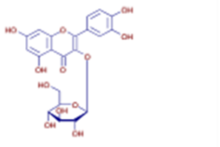	$C_{21}H_{20}O_{12}$	465,10211	5,793	332625187	99,6	2,777973606	Antiaging [60]	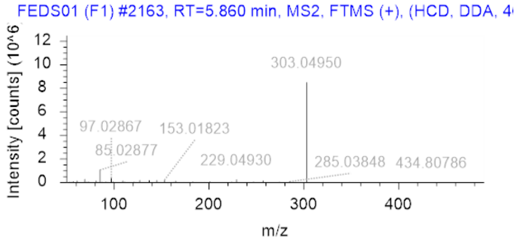 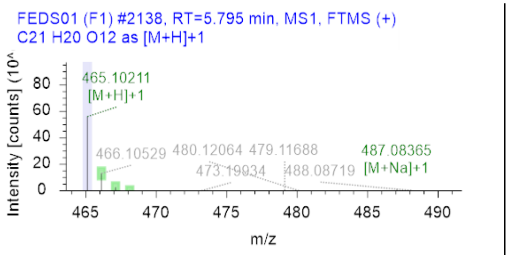
12	Bis(4-ethylbenzylidene)sorbitol 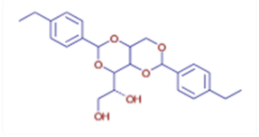	$C_{24}H_{30}O_{6}$	415,21103	11,57	50116433,5	99,6	0,508263609	-	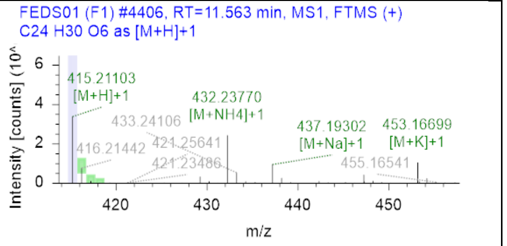 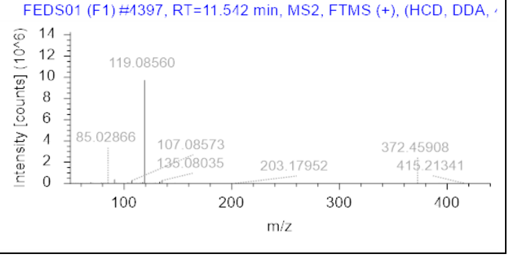

13	Linoleoyl Ethanolamide 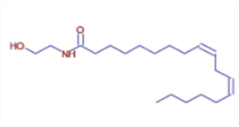	$C_{20}H_{37}NO_{2}$	324,28903	13,595	36095348,39	99,6	0,301456201	-	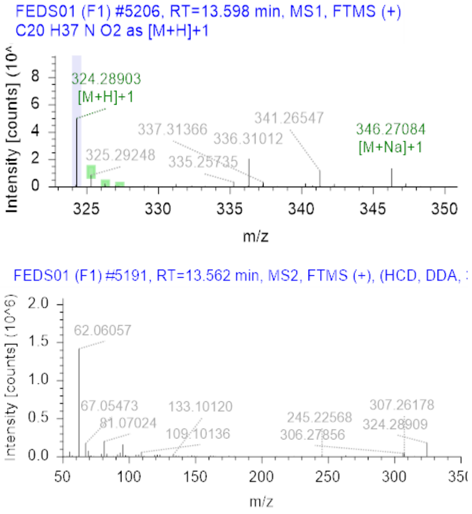
14	9(Z),11(E),13(E)-Octadecatrienoic Acid methyl ester 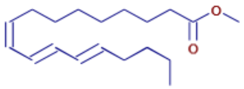	$C_{19}H_{32}O_{2}$	293,24686	16,335	78677439,58	99,5	0,657087494	-	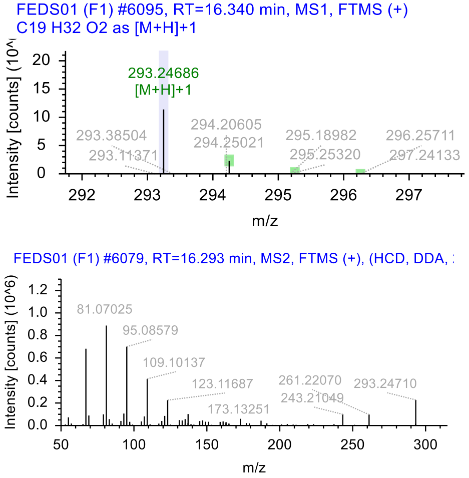

15	Ellagic acid 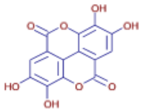	$C_{14}H_6O_8$	303,01331	5,42	51666305,12	99,5	0,43149959	Antiaging[61]	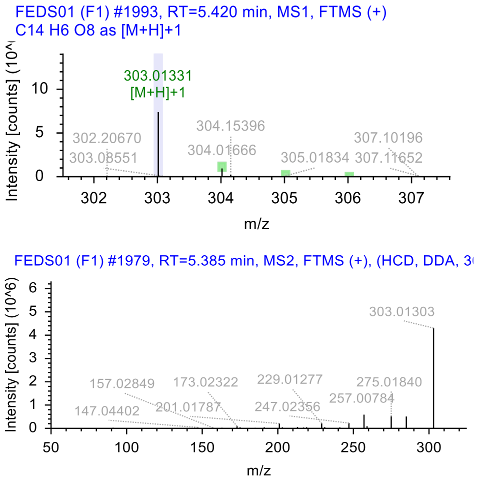
16	L-Glutamic acid 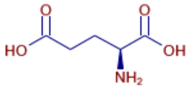	$C_5H_9NO_4$	148,06033	0,858	132891279,3	99,4	1,109863236	-	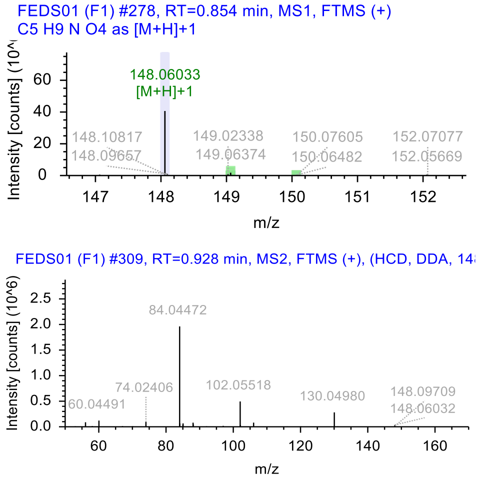

17	(2S,3R,4S,5R,6R)-5-hydroxy-6-(hydroxymethyl)-3,4-bis(3,4,5-trihydroxybenzoyloxy)oxan-2-yl 3,4,5-trihydroxybenzoate 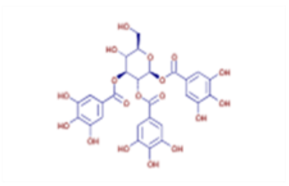	$C_{27}H_{24}O_{18}$	619,09198	4,853	68084383,29	99,4	0,568617853	-	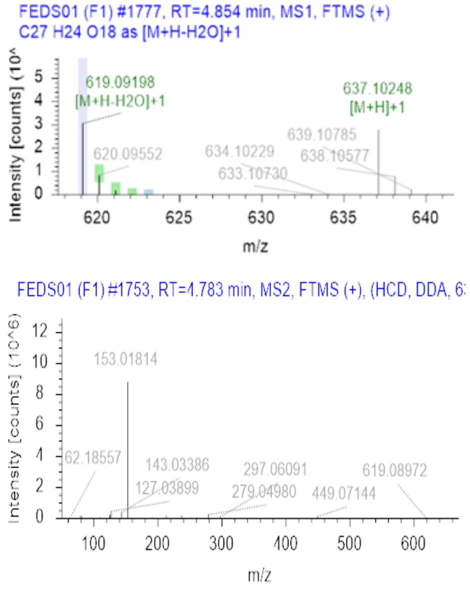
18	Dodecyltrimethylammonium 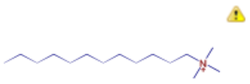	$C_{15}H_{33}N$	228,26852	10,49	9404674,757	99,4	0,078544678	-	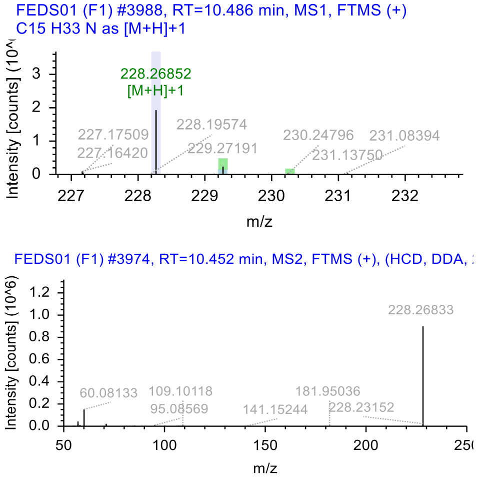

19	DEET 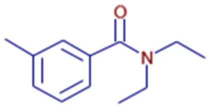	$C_{12}H_{17}NO$	192,13809	9,304	9388939,765	99,4	0,078413265	-	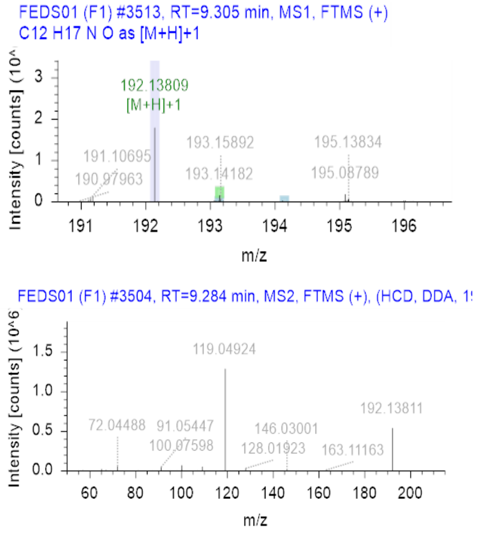
20	Kaempferol 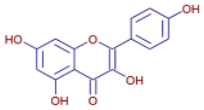	$C_{15}H_{10}O_6$	287,05453	6,267	85498031,69	99,3	2,502902113	Antiaging[62]	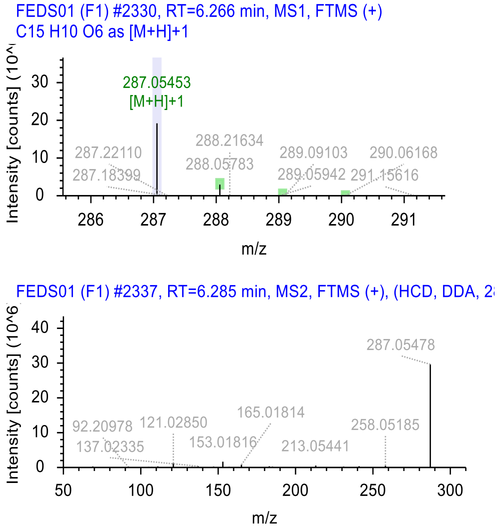

21	PIcolinic acid 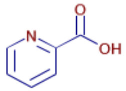	$C_6 H_5 N O_2$	124,03941	0,896	46348539,17	99,3	0,57241337	-	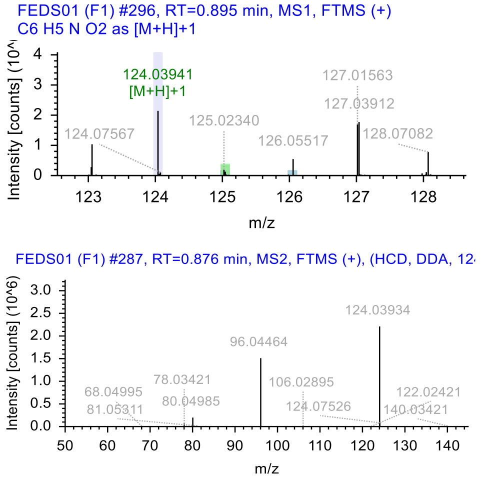
22	Bis(2-ethylhexyl)adipate 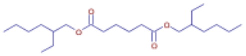	$C_{22}H_{42}O_4$	371,31464	17,307	337588532,9	99,2	2,819425801	-	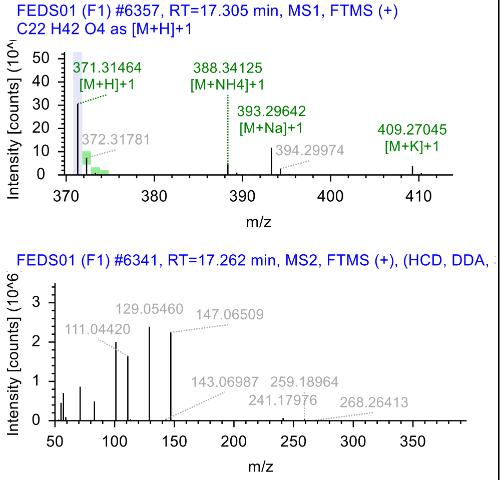

23	7-hydroxy-6-methoxy-2H-chromen-2-one 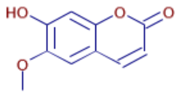	$C_{10}H_8O_4$	193,04922	5,782	174061528,3	99,2	1,56369957	-	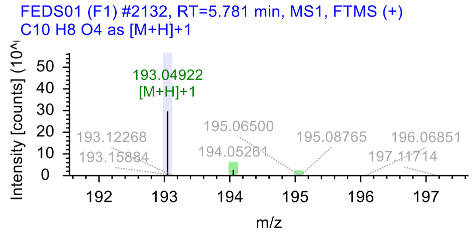 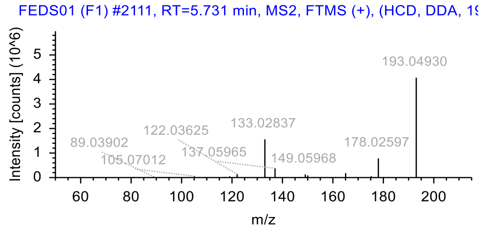
24	L-Isoleucine 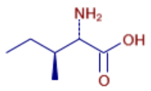	$C_6H_{13}NO_2$	132,10179	0,849	105713464,7	99,2	0,882883276	-	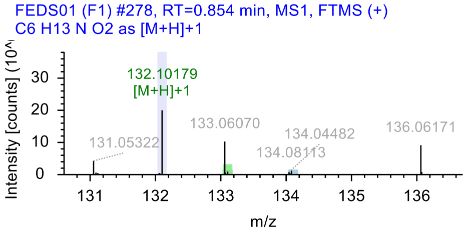 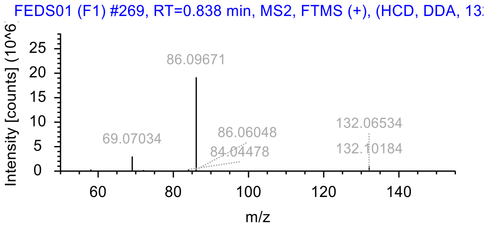

25	Oleamide<br>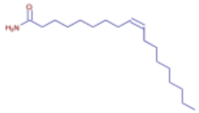 	$C_{18}H_{35}NO$	282,27899	14,868	193626842,7	99,1	1,617106219	-	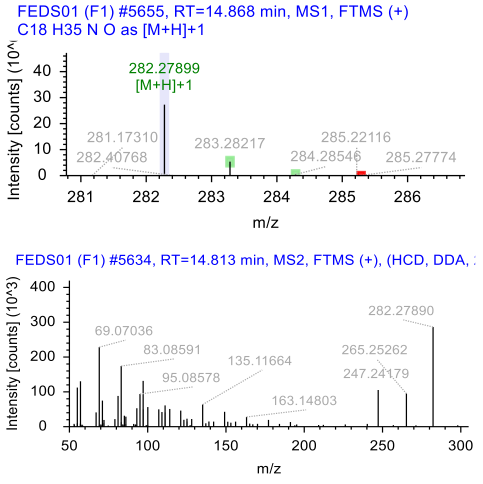
26	1-Linoleoyl glycerol<br>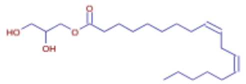 	$C_{21}H_{38}O_4$	355,28375	12,441	61896808,11	99,1	1,257432727	-	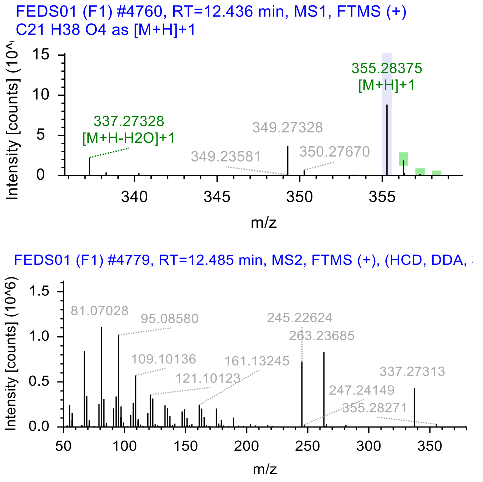

27	Nicotinamide 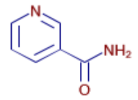	$C_6 H_6 N_2O$	123,05528	1,1	33357601	99,1	0,278591456	-	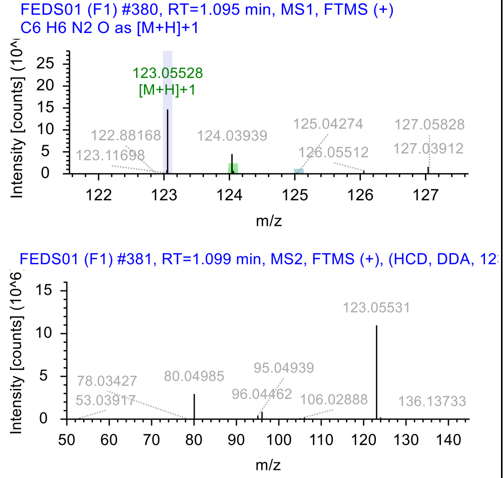
28	Phloretin 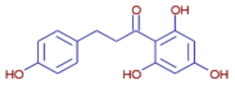	$C_{15} H_{14} O_5$	275,09116	6,829	6088640,983	99	0,05085028	-	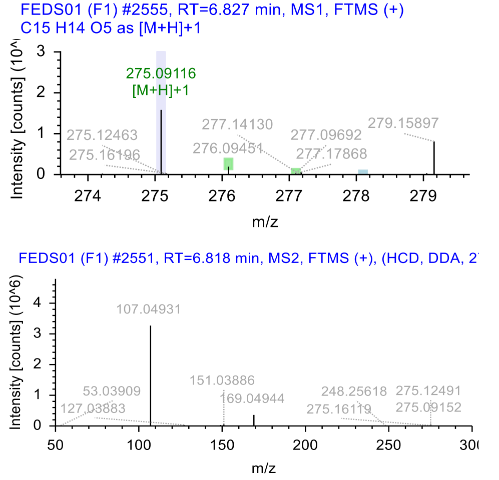

29	D-Glucosamine<br>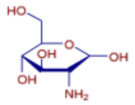	$C_6 H_{13} NO_5$	218,04253	0,746	90186719,71	98,8	0,753209128	-	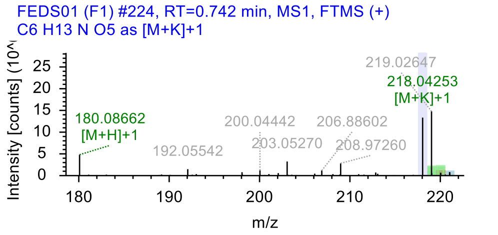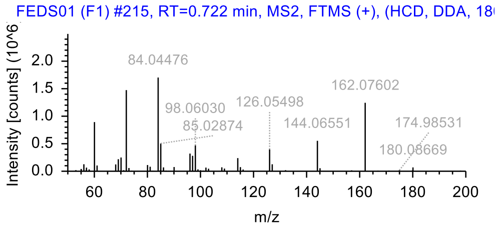
30	Trifolin<br>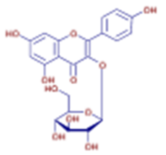	$C_{21} H_{20} O_{11}$	449,10730	6,341	363899828,3	98,7	4,139261355	-	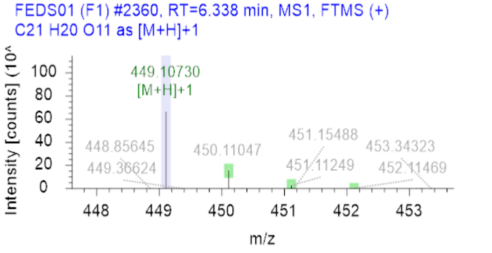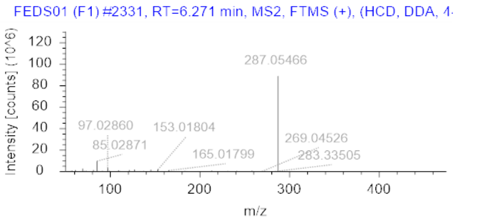

<table>
<tr><td>31</td><td>(+)-ar-Turmerone
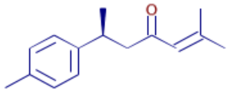</td><td>C_{15} H_{20} O</td><td>217,15837</td><td>12,985</td><td>44466202,66</td><td>98,7</td><td>0,371366758</td><td>-</td><td>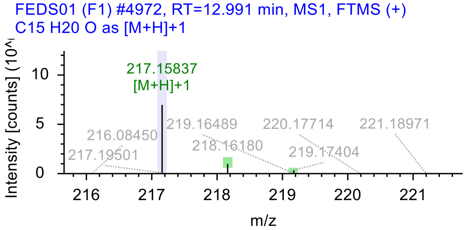

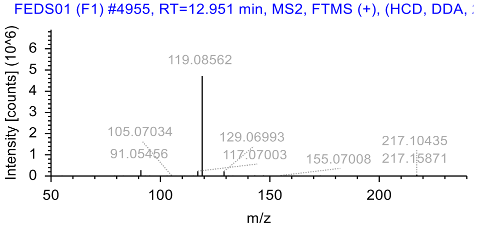
</td></tr>
<tr><td>32</td><td>Rutin
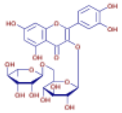</td><td>$C_{27}H_{30}$ O_{16}</td><td>611,15979</td><td>5,59</td><td>12037206,44</td><td>98,7</td><td>0,100530697</td><td>-</td><td>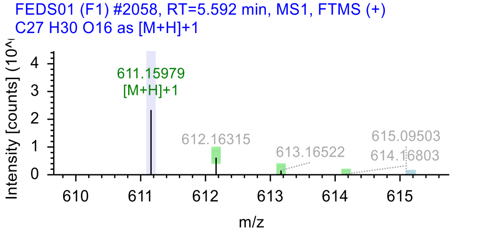

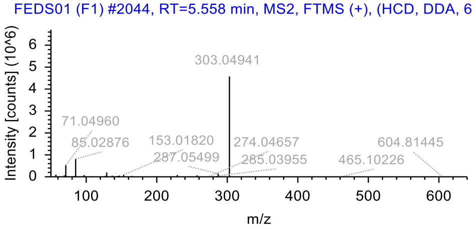
</td></tr>
</table>

33	Bis(3,5,5-trimethylhexyl) phthalate 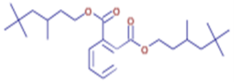	$C_{26}H_{42}O_4$	419,31497	18,052	404247468,1	98,6	3,376138791	-	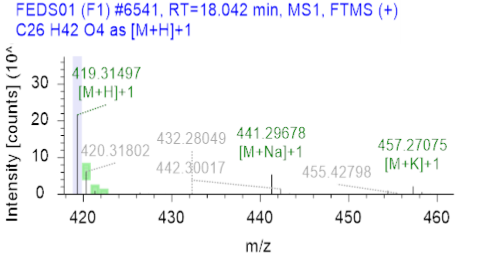 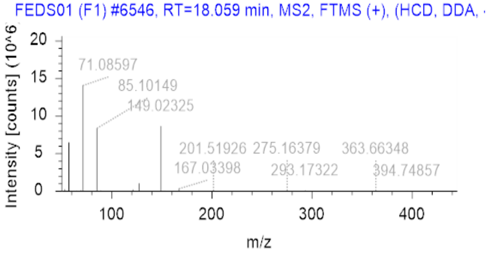
34	Quercetin 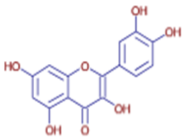	$C_{15}H_{10}O_7$	303,0494	6,339	296117767,9	98,5	5,78369812	Antiaging[60]	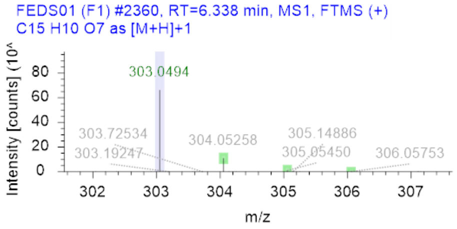

									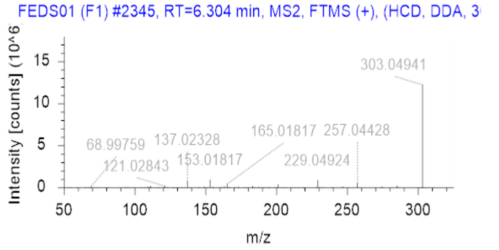
35	Adenine 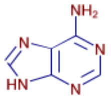	$C_5 H_5 N_5$	136,06160	0,832	116570292,3	98,4	0,973555846	-	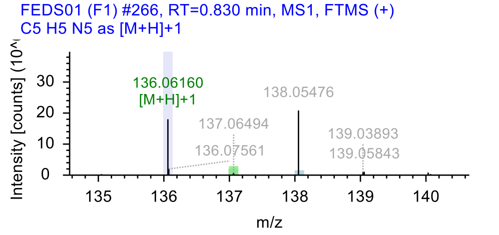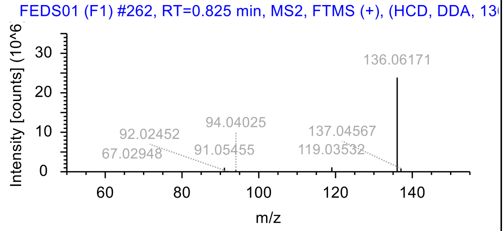
36	D-(-)-Quinic acid 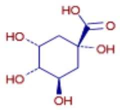	$C_7 H_{12} O_6$	193,07080	0,852	16868155,75	98,4	0,140877159	-	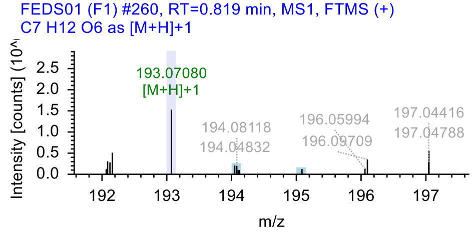

									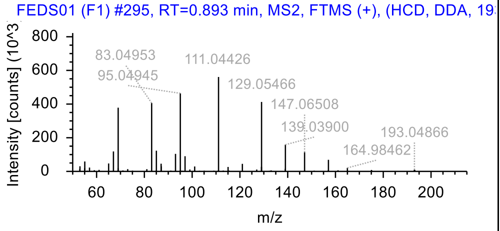
37	Monoolein 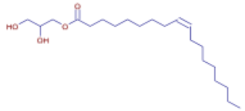	$C_{21}H_{40}O_4$	357,29929	14,839	8216845,931	98,4	0,068624332	-	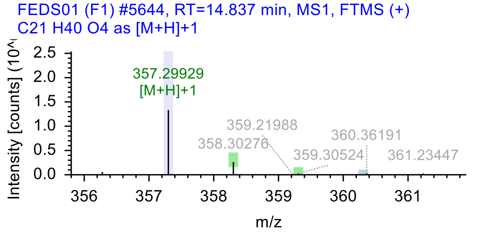 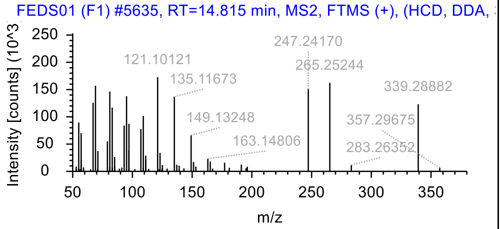
38	Kynurenic acid 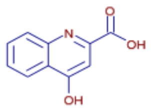	$C_{10}H_7NO_3$	190,04979	3,211	79983177,79	98,1	0,66799258	-	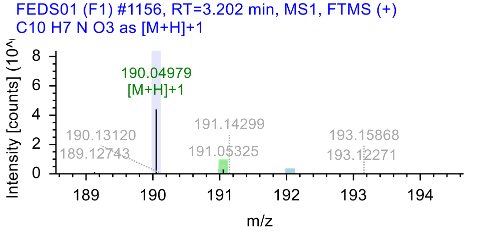

									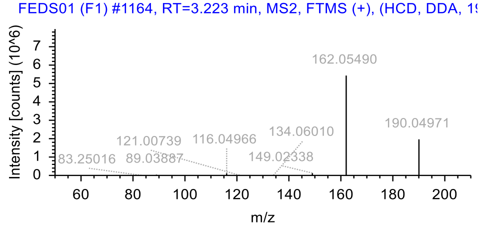
39	Methyl isonicotinate 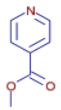	$C_7 H_7 N O_2$	138,05476	0,827	141200165,5	98	1,179256257	-	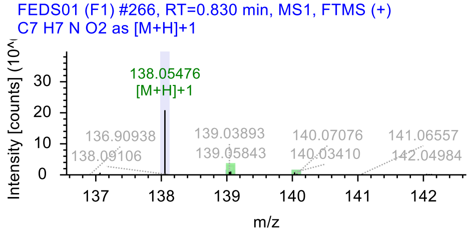 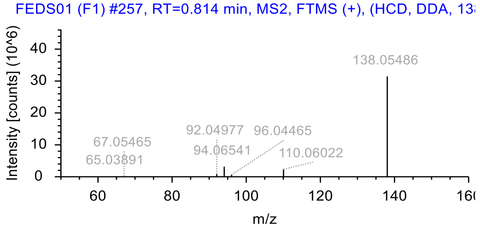
40	2,3,4,9-Tetrahydro-1H-β-carboline-3-carboxylic acid 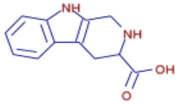	$C_{12}H_{12}NO_2$	217,09695	3,502	37882015,57	97,9	0,316377844	-	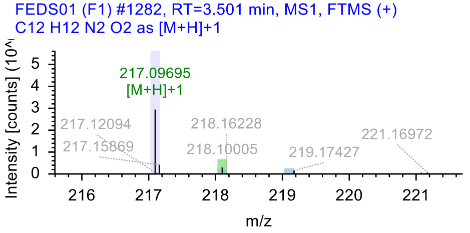

									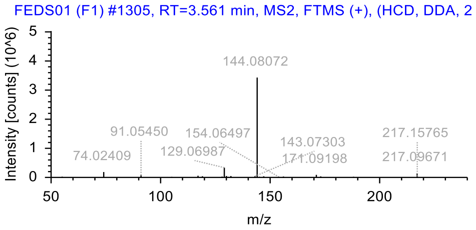
41	NP-013538 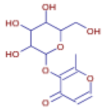	$C_{12} H_{16} O_8$	289,09149	1,5	24716261,04	97,9	0,206421893	-	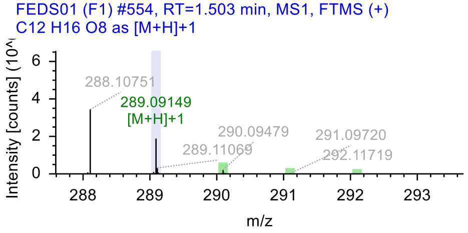 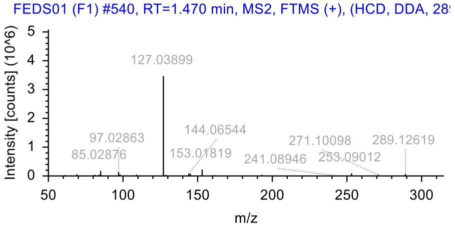

42	Palmitoleic acid<br>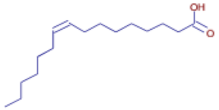 	$C_{16}H_{30}O_2$	255,23172	12,222	8370349,539	97,9	0,069906342	-	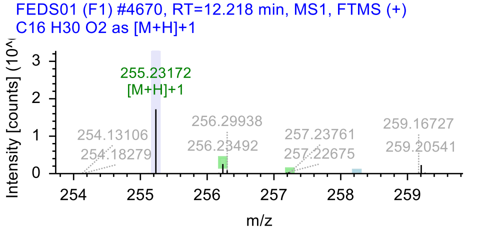<br><br>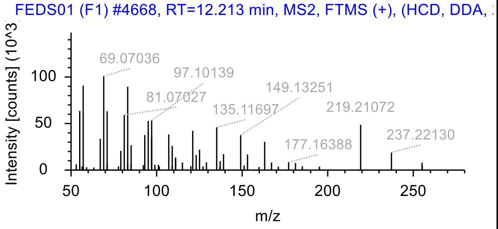
43	L(-)-2-Amino-3-phenyl-1-propanol<br>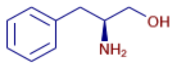 	$C_9H_{13}NO$	152,10695	10,436	6309837,554	97,8	0,052697639	-	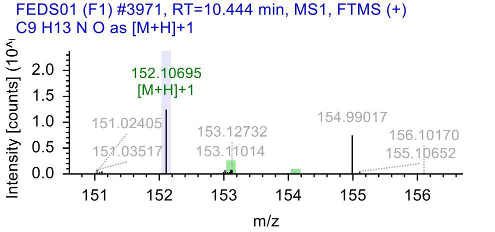<br><br>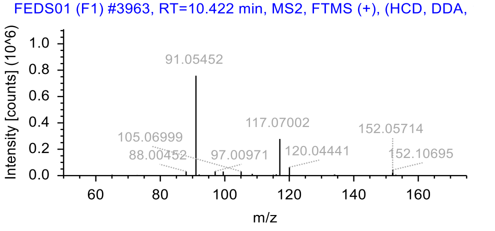

44	Dodecylamine 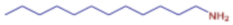	$C_{12}H_{27}N$	186,22137	9,681	20032490,82	97,7	0,167304621	-	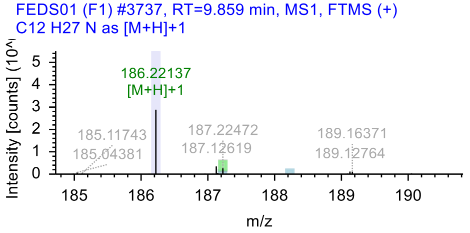 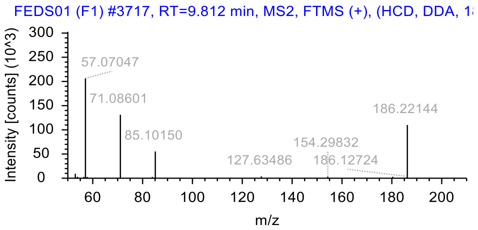
45	(-)-Caryophyllene oxide 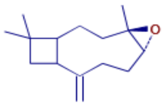	$C_{15}H_{24}O$	203,17908	9,883	203826483,2	97,5	4,874359482	-	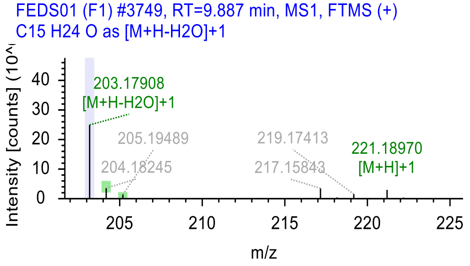 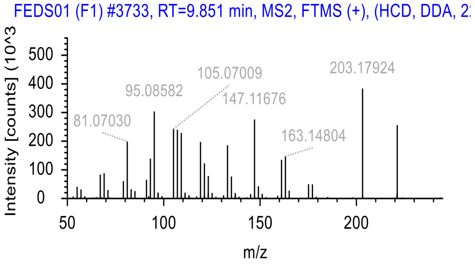

46	Stearamide 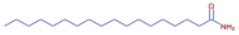	$C_{18}H_{37}NO$	284,29443	15,825	10934462,19	97,5	0,365629035	-	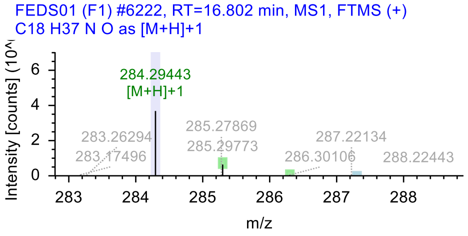 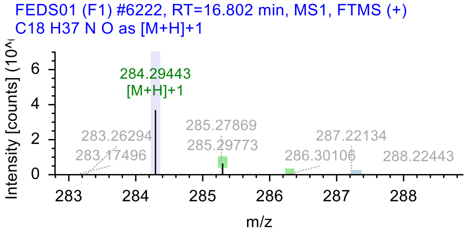
47	Caffeine 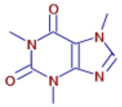	$C_8H_{10}N_4O_2$	195,08759	4,171	10050318,15	97,3	0,083936874	-	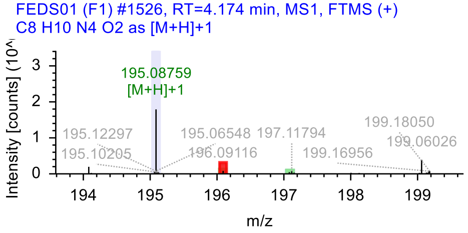

									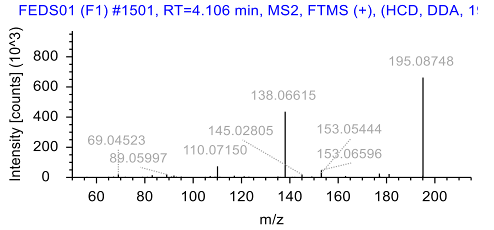
48	Coumarin 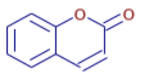	$C_9H_6O_2$	147,04382	6,893	29485323,55	97,2	0,246251498	-	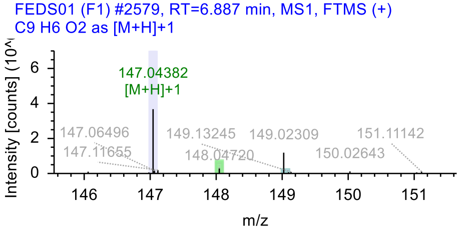 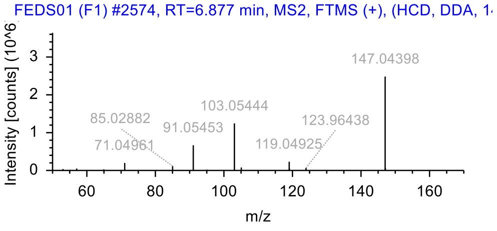
49	1-Stearoylglycerol 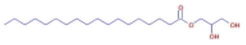	$C_{21}H_{42}O_4$	359,31516	12,822	9733423,348	97	0,167655811	-	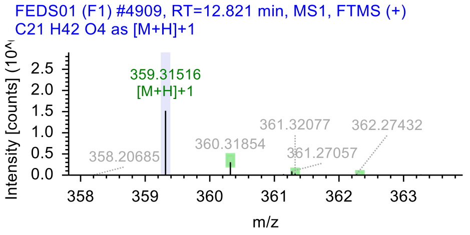

									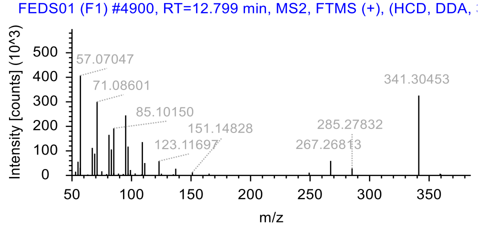
50	5-Hydroxymethyl-2-furaldehyde 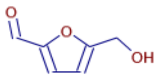	$C_6H_6O_3$	127,03899	5,143	24908257,99	96,9	1,914200208	-	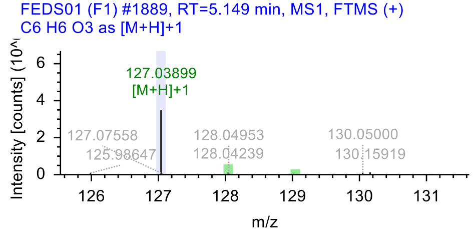 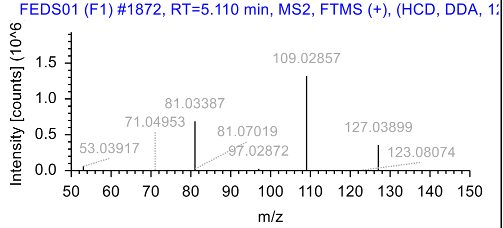
51	Fraxetin 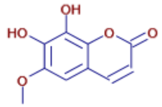	$C_{10}H_8O_5$	209,04448	4,933	6486422,678	96,6	0,054172419	-	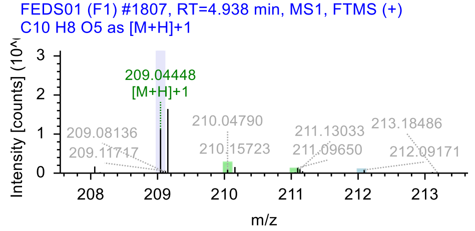

									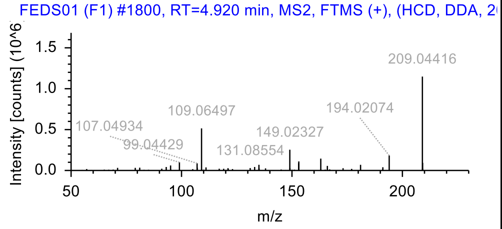
52	Tiliroside 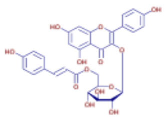	C30 H26 O	595,14368	7,774	15053616,15	96,4	0,125722735	-	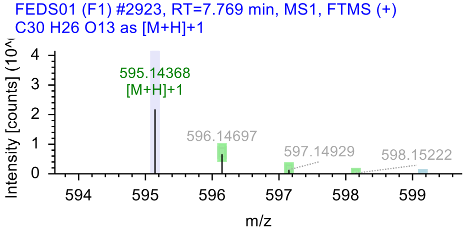 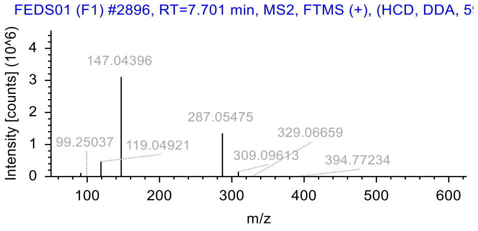
53	NP-015948 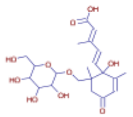	C_{21} H_{30} O_{10}	443,19055	5,722	6012822,01	95,7	0,050217066	-	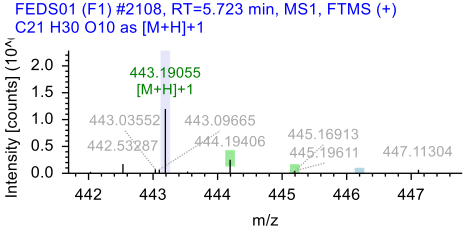 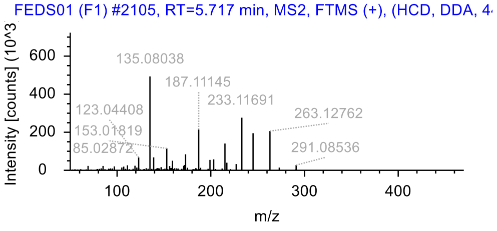

54	Hexadecanamide 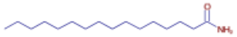	$C_{16}H_{33}NO$	256,26331	14,443	37648764,32	95	0,314429808	-	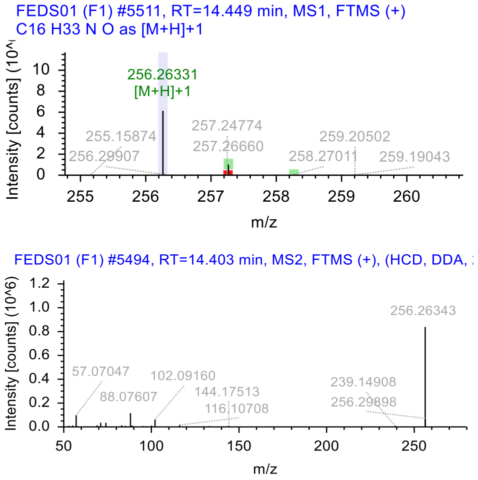
55	5,6,7-Trimethoxy-2H-chromen-2-one 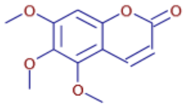	$C_{12}H_{12}O_5$	237,07571	7,811	24584578,2	94,1	0,205322122	-	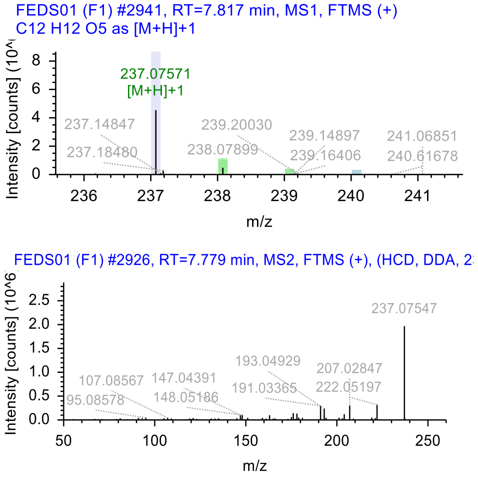

56	Betaine 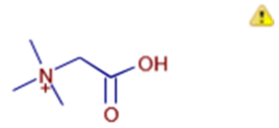	$C_5 H_{11} NO_2$	118,08624	0,816	679599955,7	94	5,67579009	-	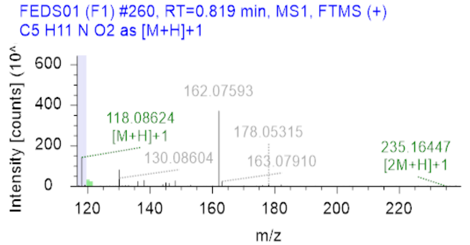 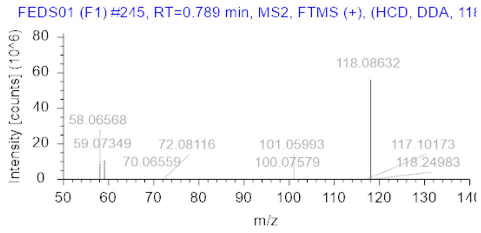
57	6-Methylnicotinamide 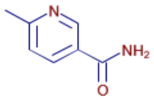	$C_7H_8N_2O$	137,07088	1,105	13064505,79	93,7	0,109110355	-	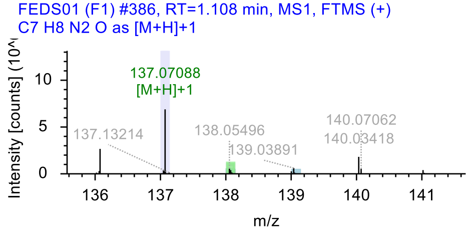 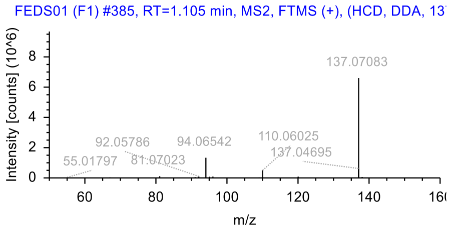

58	13(S)-HOTrE 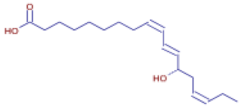	$C_{18}H_{30}O_3$	295,22665	8,988	9408468,278	93,6	0,07857636	-	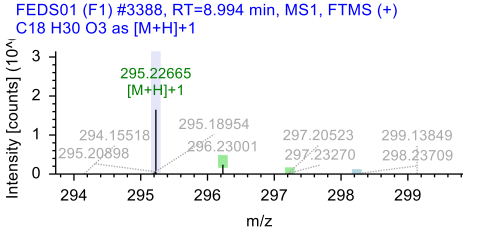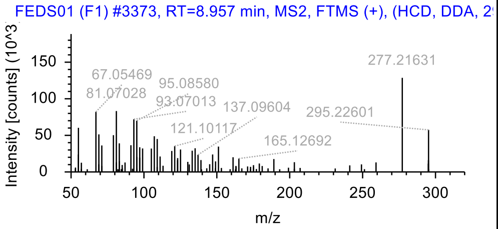
59	NP-019401 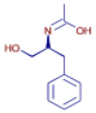	$C_{11}H_{15}NO_2$	194,11739	11,757	9896822,339	93	0,082654929	-	

									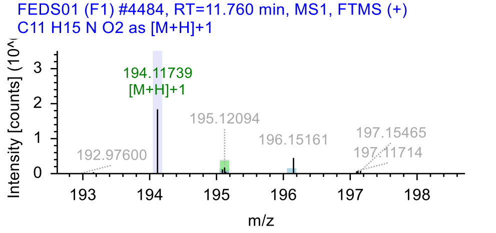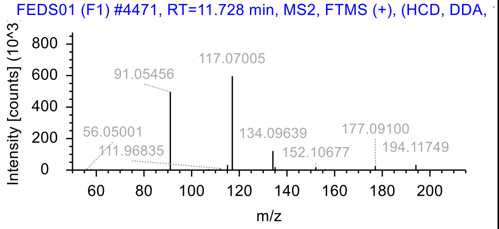
60	Choline 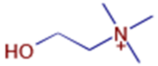	C 5H13 NO	104,10712	0,773	741381738,2	92,7	6,191770742	-	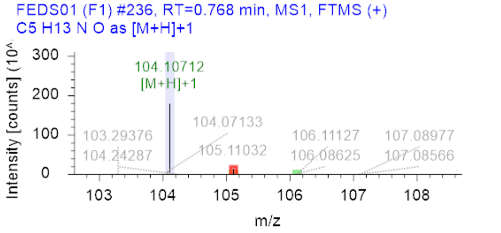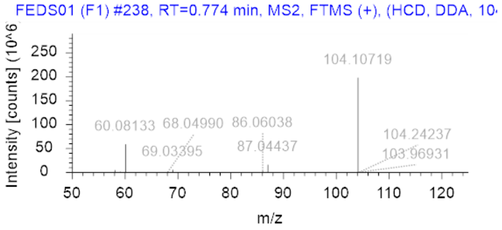
61	12-oxo Phytodienoic Acid 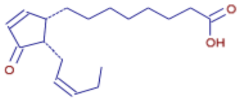	$C_{18}H_{28}O_3$	293,21100	12,592	17800946,91	92,7	0,360954072	-	

									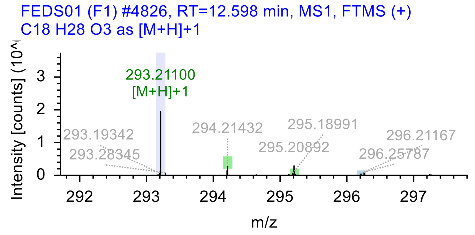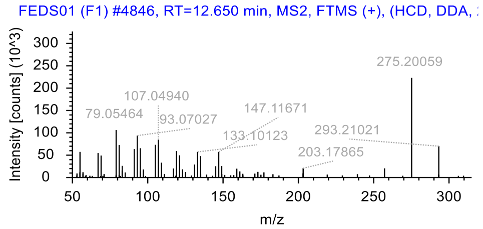
62	N,N-Dimethyldecylamine N-oxide<br>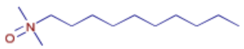	$C_{12}H_{27}NO$	202,21646	8,461	4608780,243	92,7	0,038490981	-	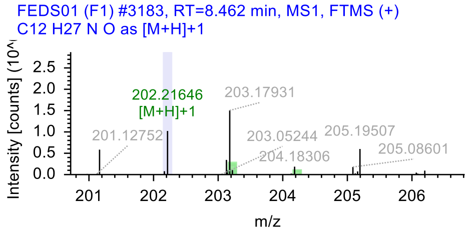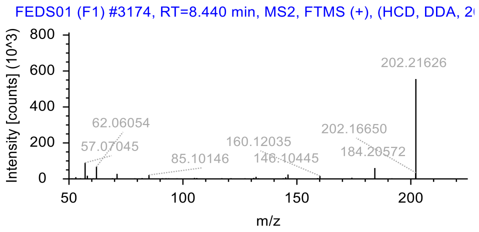

63	4-Coumaric acid 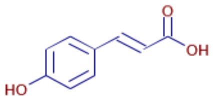	$C_9 H_8 O_3$	165,05469	6,072	20020133,13	91,2	0,485690603	-	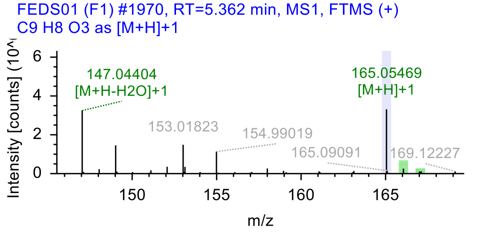 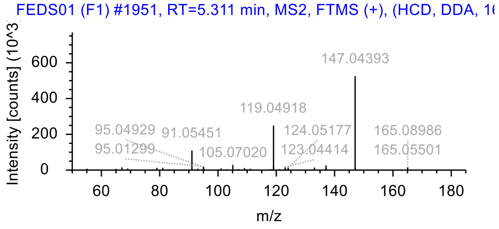
64	5-hydroxy-2-(4-hydroxyphenyl)-8,8-dimethyl-4H,8H-pyrano[3,2-g]chromen-4-one 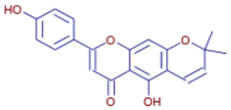	$C_{20}H_{16} O_5$	337,10651	11,542	14904611,08	91,1	0,124478295	-	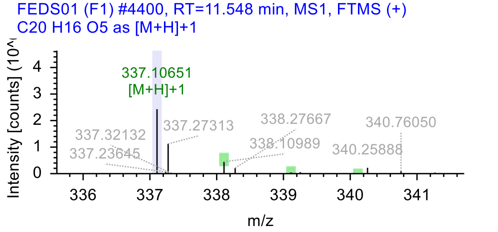 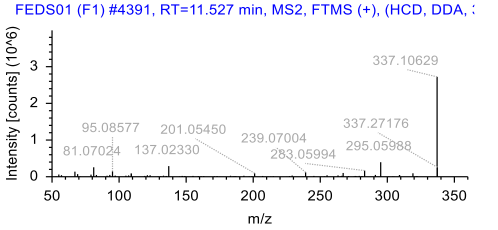

65	1-Tetradecylamine 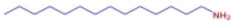	$C_{14}H_{31}N$	214,25253	10,343	735142989,4	91	6,139666812	-	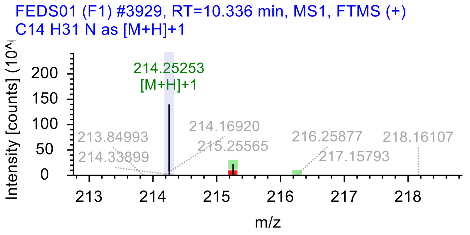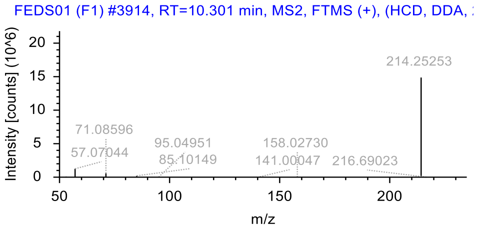
66	1-Aminocyclohexanecarboxylic acid 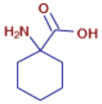	$C_7H_{13}NO_2$	144,10191	1,1	12622302,24	91	0,316814175	-	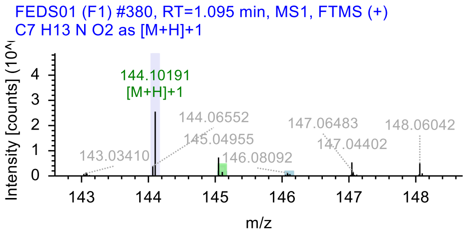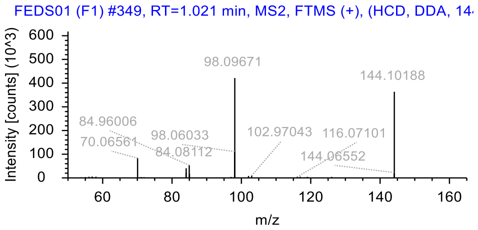

67	NP-015980 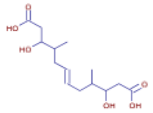	$C_{14}H_{24}O_6$	311,14621	6,424	14453133,01	90,7	0,120707702	-	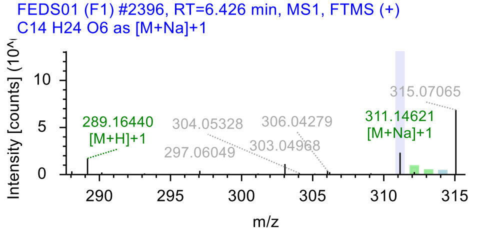 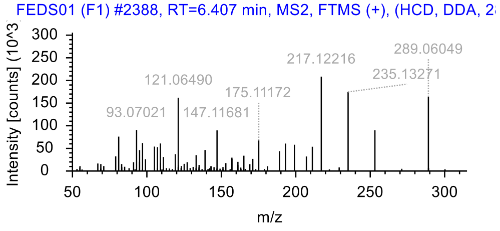
68	Esculetin 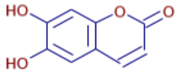	$C_9H_6O_4$	179,03391	4,112	12508095,06	89,8	0,1044634	-	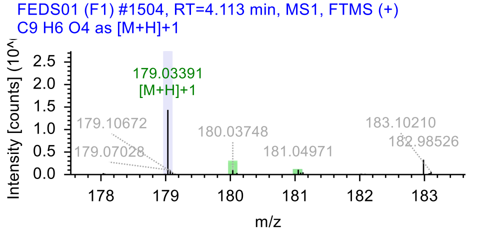 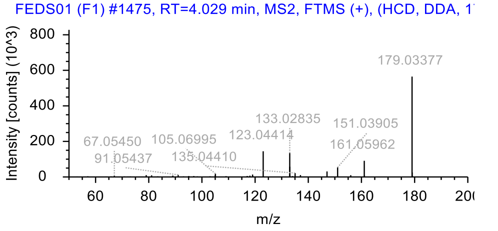

69	Diisobutyl adipate 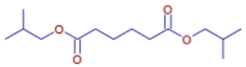	$C_{14}H_{26}O_4$	259,18994	17,305	6962837,103	89,2	0,058151272	-	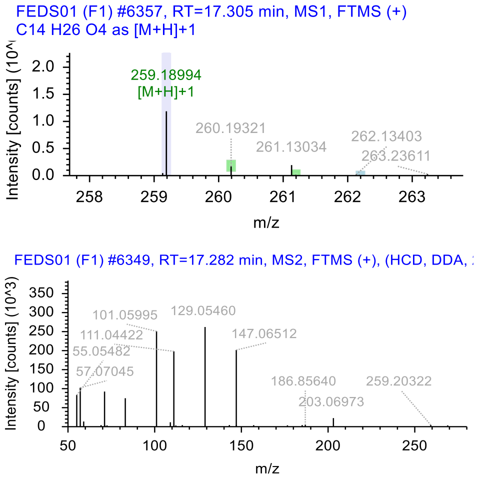
70	Sorbic acid 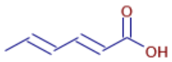	$C_6H_8O_2$	113,05990	2,779	180966381,2	88,7	1,511370306	-	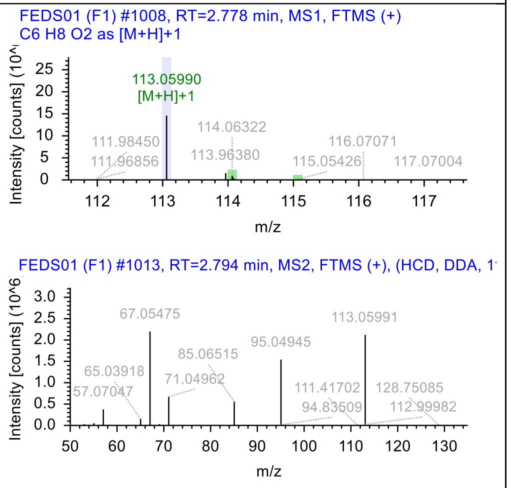
71	MPBP 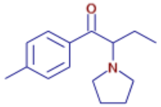	$C_{15}H_{21}NO$	232,16939	5,856	34266986,79	87,5	0,28618634	-	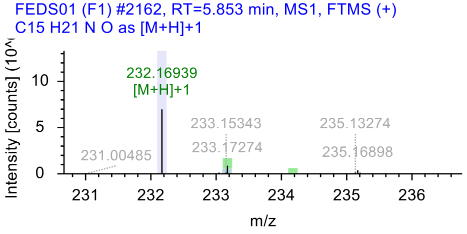

<table>
<tr><td></td><td></td><td></td><td></td><td></td><td></td><td></td><td></td><td></td><td>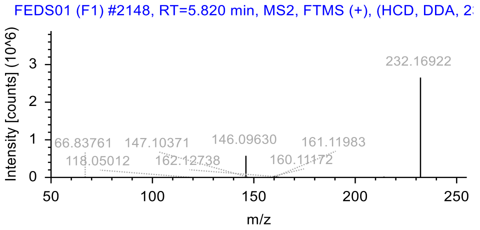
</td></tr>
<tr><td>72</td><td>Nootkatone
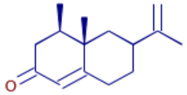</td><td>$C_{15}H_{22}O$</td><td>219,17422</td><td>12,589</td><td>12971155,77</td><td>86,4</td><td>0,108330727</td><td>-</td><td>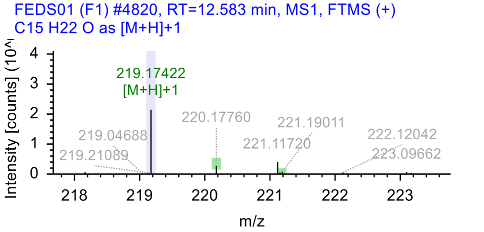

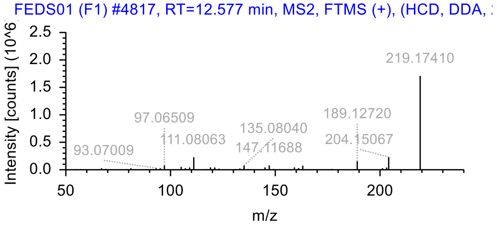
</td></tr>
<tr><td>73</td><td>Prolylleucine
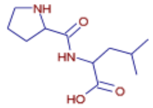</td><td>$C_{11}H_{20}N_2O$</td><td>229,15462</td><td>1,212</td><td>11399211,96</td><td>84,9</td><td>0,095202382</td><td>-</td><td>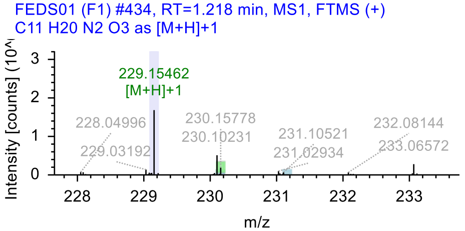
</td></tr>
</table>

									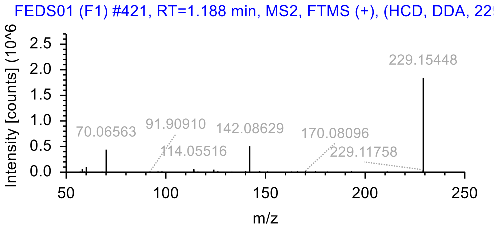
74	(1R,3S,4S,5R,7R)-4-(3-hydroxybutyl)-5-methyl-10-methylidene-8-oxatricyclo[5.3.0.0Âl,âµ]decan -9-one 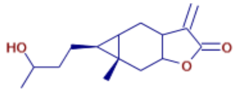	$C_{15} H_{22} O_3$	251,16371	9,839	8643522,244	84,4	0,072187789	-	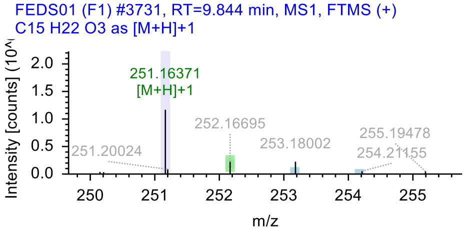 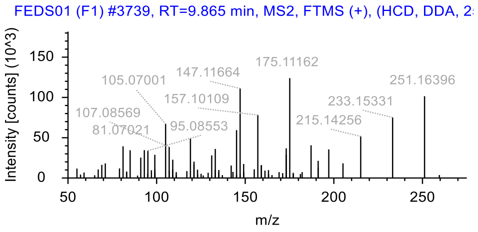
75	Methyl palmitate 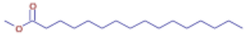	$C_{17} H_{34}$ O2	271,26300	17,11	9268269,228	84,1	0,077405465	-	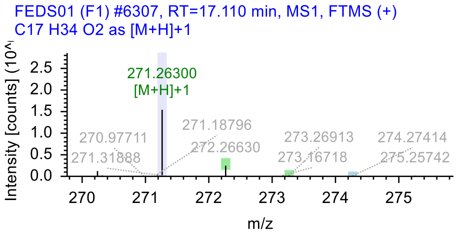

									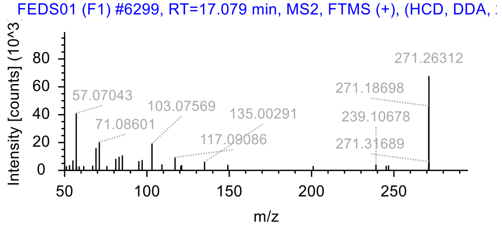
76	Desmanthin-1 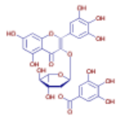	$C_{28}H_{24}O_{16}$	617,11237	5,896	155156437,3	82,8	1,295814341	-	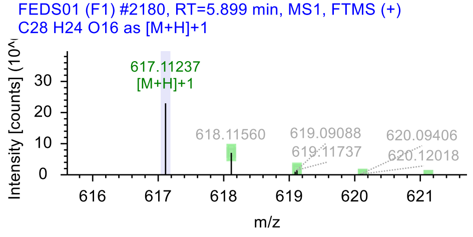 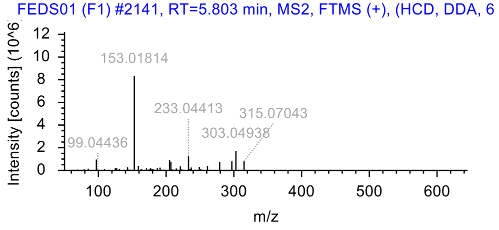
77	D-(+)-Pyroglutamic Acid 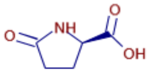	$C_5H_7NO_3$	130,04985	1,107	411853601,8	82,5	3,43966266	-	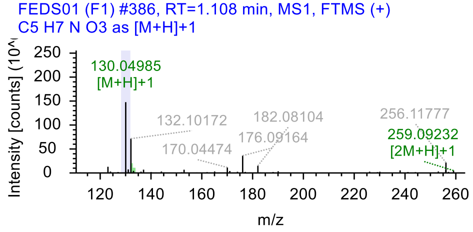 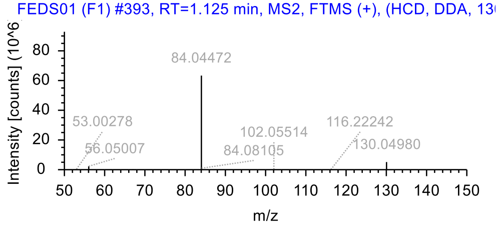

78	Triethylene glycol monobutyl ether 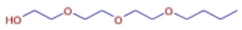	$C_{10}H_{22}O_4$	207,15872	6,504	48685224,74	82,4	0,40660261	-	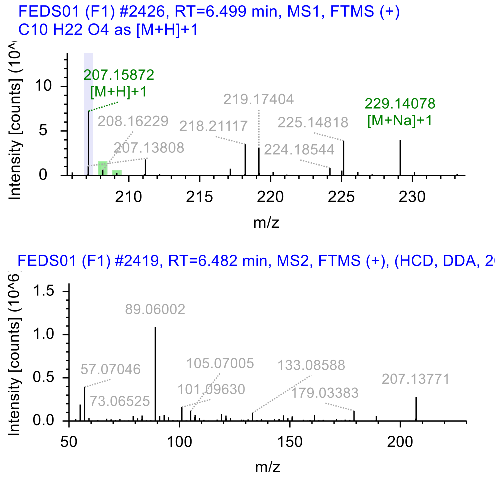
79	5-(6-hydroxy-6-methyloctyl)-2,5-dihydrofuran-2-one 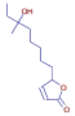	$C_{13}H_{22}O_3$	227,16400	6,039	30192505,62	82	0,252157645	-	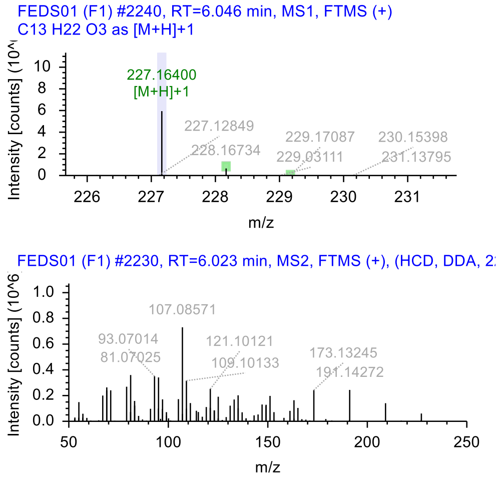

80	D-(+)-Pyroglutamic Acid 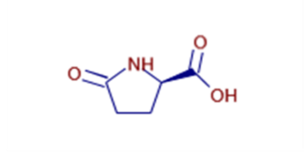	$C_5 H_7 NO_3$	130,04985	0,844	747688522	81,2	6,244442878	-	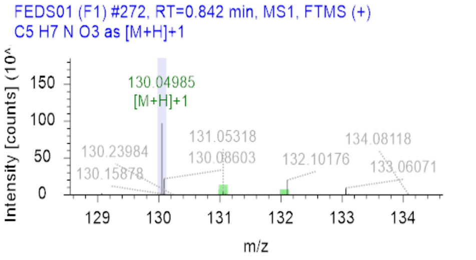 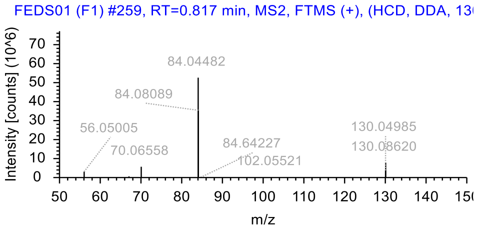
81	α-Linolenic acid 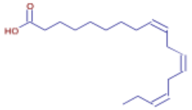	$C_{18} H_{30} O_2$	279,23172	14,535	91972102,8	81,1	0,768120046	-	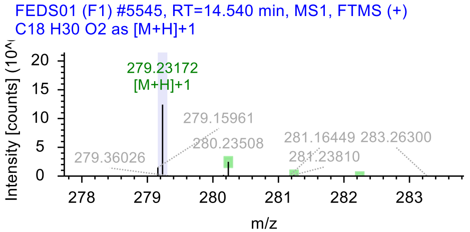 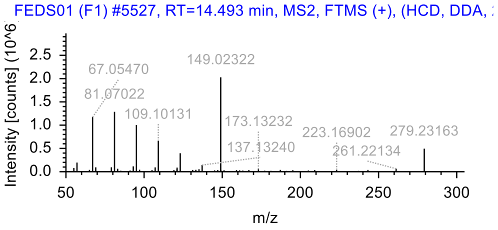

82	4-oxo-4,5,6,7-tetrahydrobenzo[b]furan-3-carboxylic acid<br>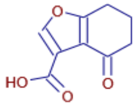	C9 H8 O4	181,04955	5,047	5520554,072	80,7	0,046105809	-	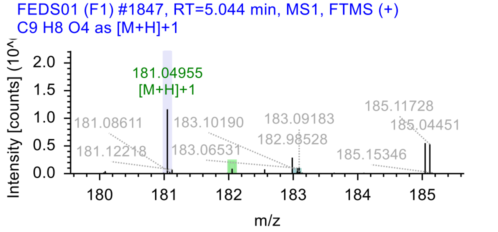<br><br>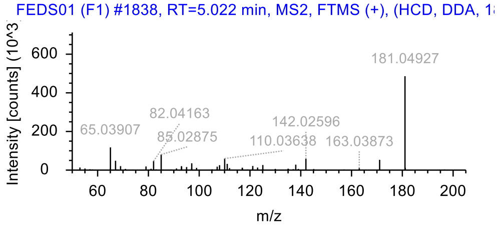
