# Supplementary material for: Formulation Development of Natural Polymeric Nanoparticles, In Vitro Antiaging Evaluation, and Metabolite Profiling of Toona sinensis Leaf Extracts
Source: Pharmaceuticals (Basel). 2025 Feb 20;18(3):288. doi: 10.3390/ph18030288 (PMC11945715; doi:10.3390/ph18030288)

Supplementary Table S2. Predicted of Ethanol Extract Compounds in Surian Leaves

Peak	Name	R.Time	I.Time	F.Time	Area	Area%	Height	Height%	A/H
1	2,2-Dimethylvaleric acid	3.772	3.733	3.808	1651351	0.53	1001524	0.92	1.65
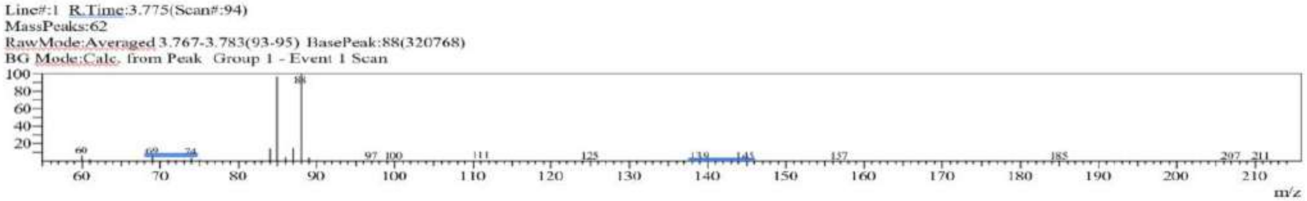									
2	2,4-Dihydroxy-2,5-dimethyl-3(2H)-furan-3-o	4.385	4.350	4.458	2472434	0.80	1313398	1.21	1.88
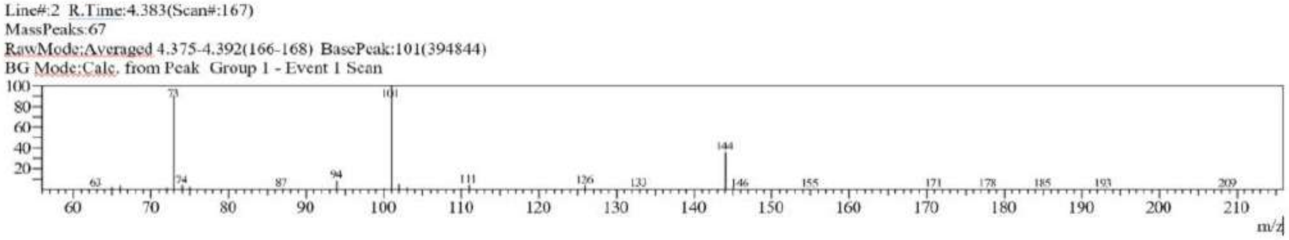									
3	Benzyloxybenzene, 2,5-difluoro-	4.924	4.858	4.992	3666052	1.18	1518899	1.40	2.41

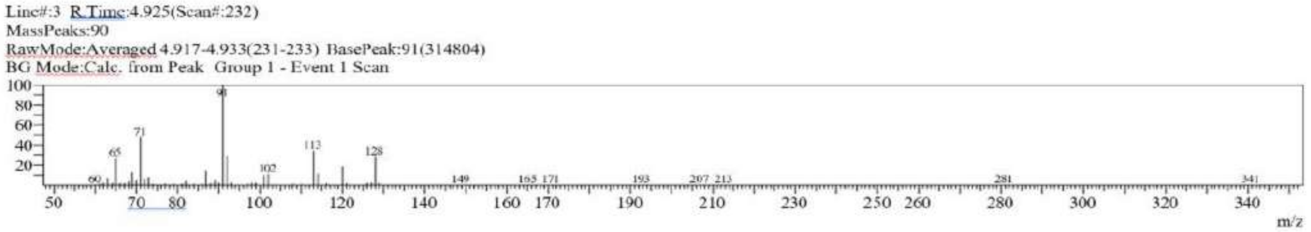									
4	Thymine	5.200	5.142	5.233	3253862	1.05	1704990	1.57	1.91
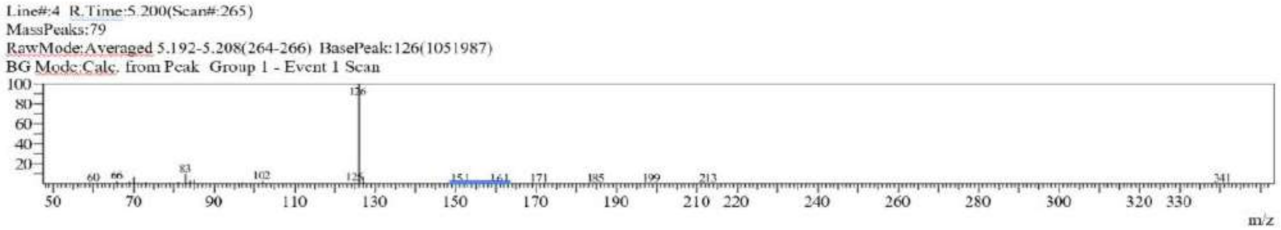									
5	Ethyl 3-hydroxy-3,6-dihydro-2H-pyridine-1-c	5.649	5.608	5.708	1244652	0.40	594882	0.55	2.09
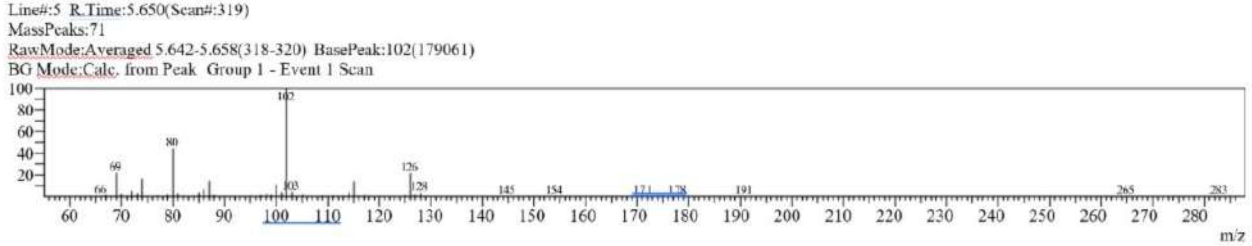									
6	4H-Pyran-4-one, 2,3-dihydro-3,5-di-hydroxy-6	5.752	5.717	5.792	4938337	1.60	2454911	2.26	2.01

<table>
<tr><td colspan="10">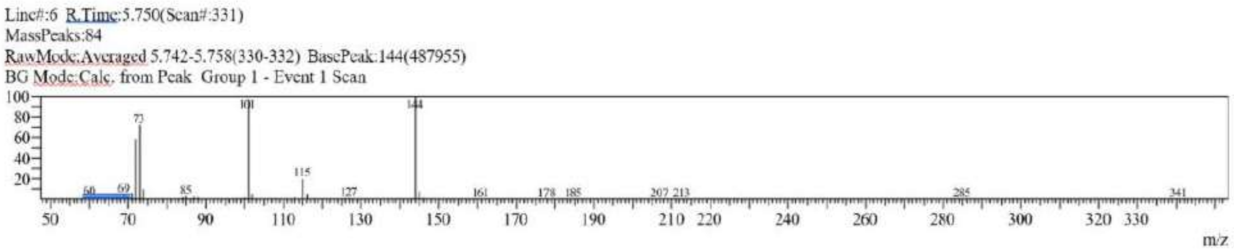
</td></tr>
<tr><td>7</td><td>Benzoic acid</td><td>5.857</td><td>5.792</td><td>5.908</td><td>2943018</td><td>0.95</td><td>976925</td><td>0.90</td><td>3.01</td></tr>
<tr><td colspan="10">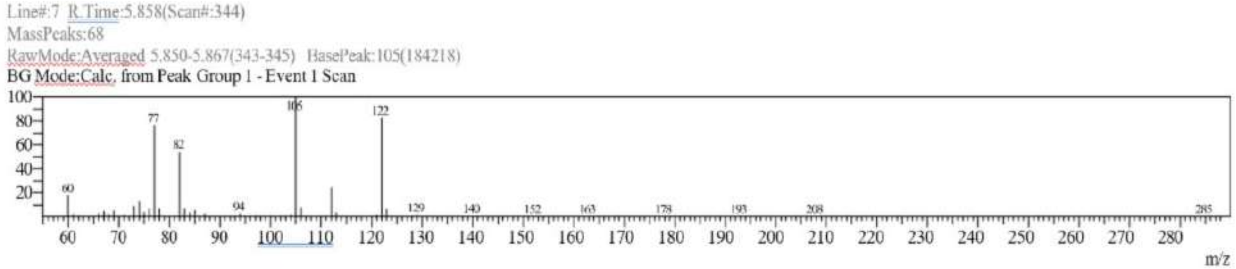
</td></tr>
<tr><td>8</td><td>4-Vinylphenol</td><td>6.340</td><td>6.292</td><td>6.383</td><td>5240603</td><td>1.69</td><td>2492270</td><td>2.29</td><td>2.10</td></tr>
<tr><td colspan="10">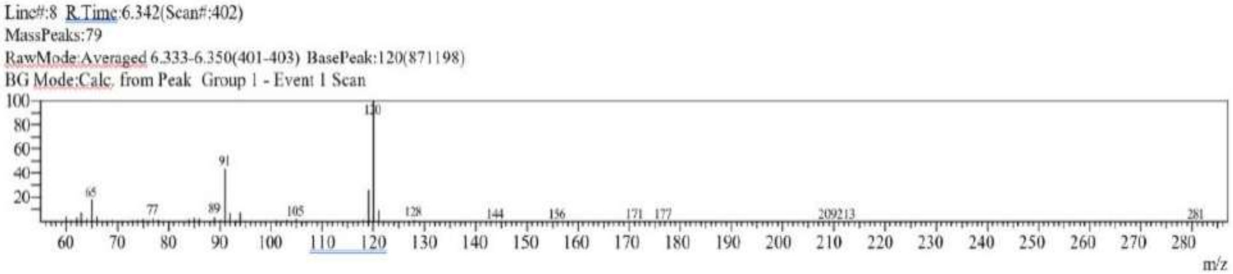
</td></tr>
<tr><td>9</td><td>2-Methoxy-4-vinylphenol</td><td>7.170</td><td>7.125</td><td>7.217</td><td>1671475</td><td>0.54</td><td>896542</td><td>0.82</td><td>1.86</td></tr>
<tr><td colspan="10"></td></tr>
</table>

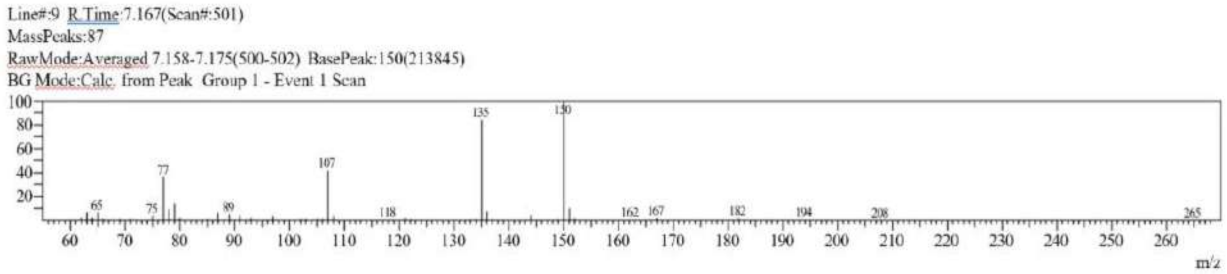

10	1,2,3-Benzenetriol	7.920	7.733	8.283	49661489	16.04	2980904	2.74	16.66

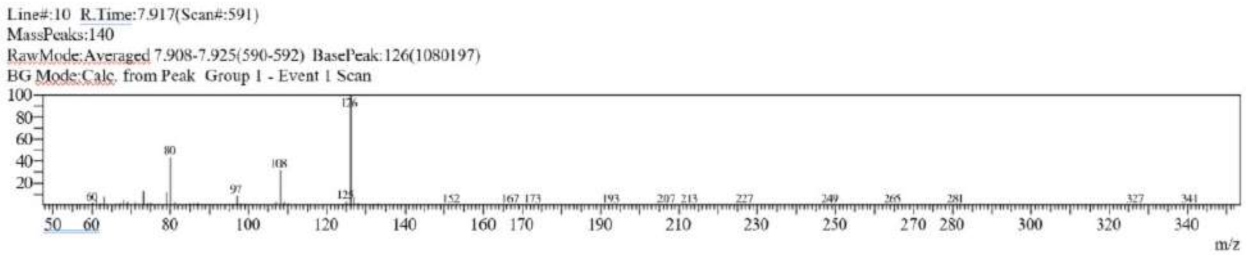

11	.gamma.-Glutamyl-(S)-Allyl-L-Cys-teine, N-is	10.108	10.058	10.175	3527739	1.14	1740191	1.60	2.03

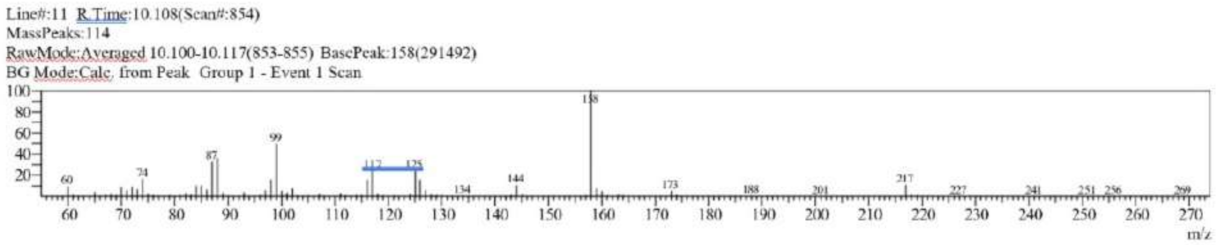

12	Isospathulenol	10.400	10.342	10.450	6720051	2.17	2786298	2.56	2.41

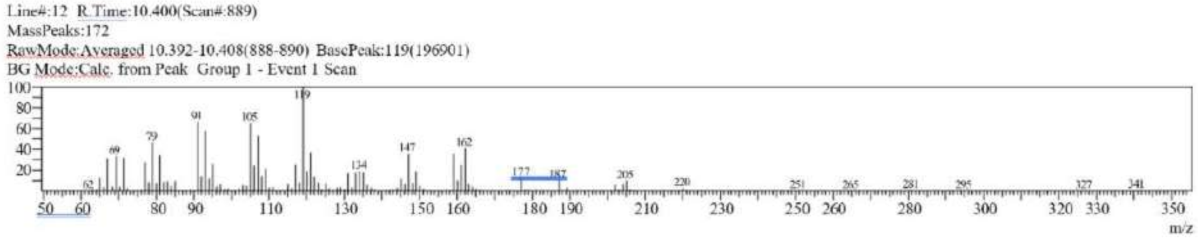

13	.alpha.-Cadinol	10.687	10.642	10.725	2714070	0.88	1056986	0.97	2.57

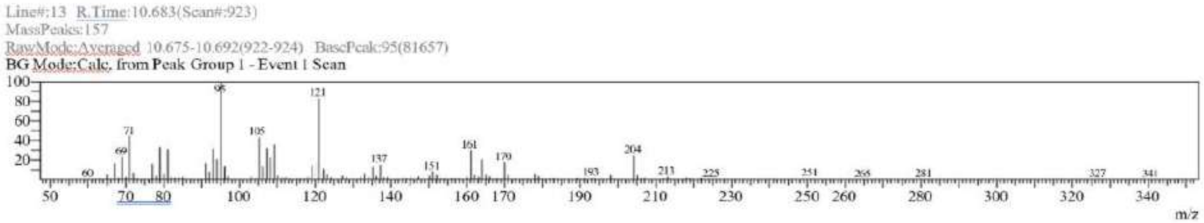

14	1,1,4,7-Tetramethyldecahydro-1H-cyclopropa	10.750	10.725	10.808	2581544	0.83	1167996	1.07	2.21

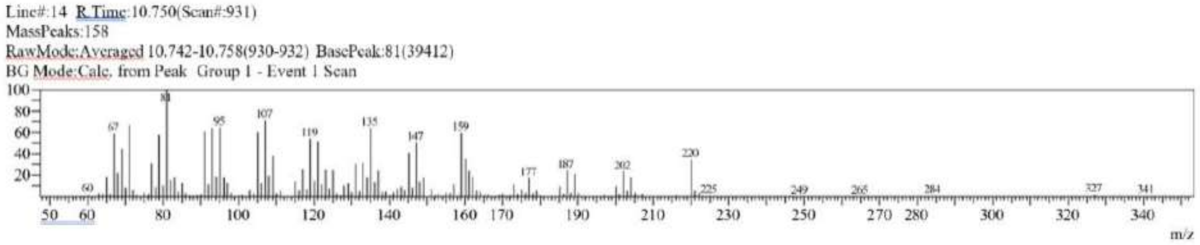

15	L-Phenylalanine, N-acetyl-, methyl ester	11.025	10.975	11.058	3044173	0.98	1628511	1.50	1.87

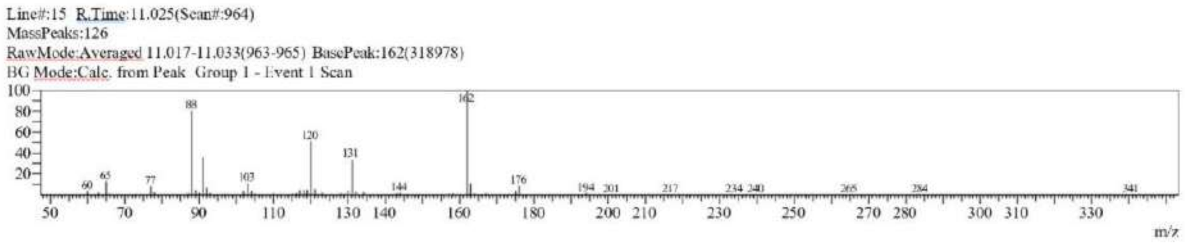

16	2-Propenoic acid, 3-(4-methoxy-phenyl)-	11.093	11.058	11.158	2926957	0.95	1210554	1.11	2.42

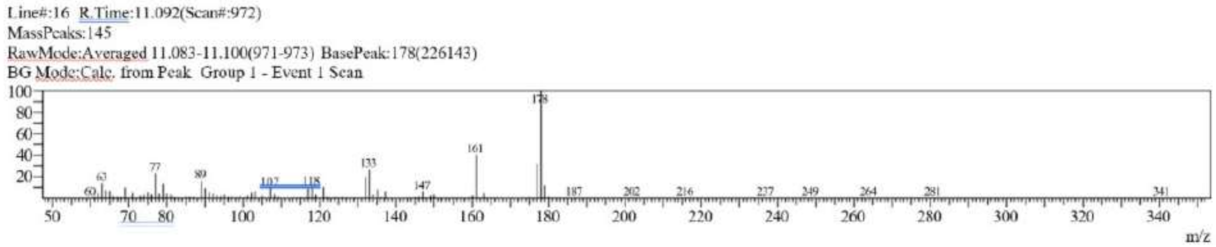

17	Tetradecanoic acid	11.596	11.475	11.667	2699751	0.87	868496	0.80	3.11

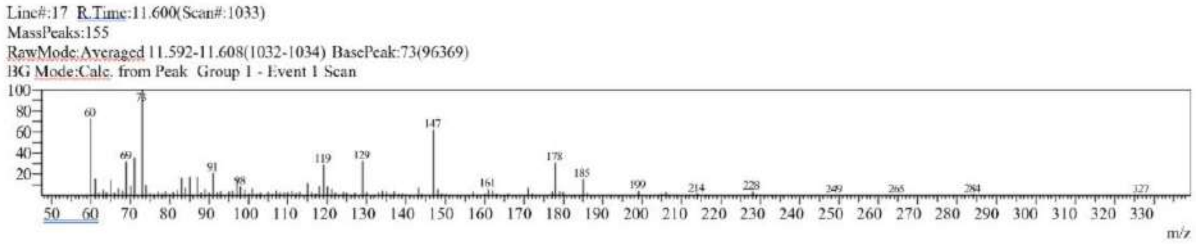

18	1,4-Azulenediol, 1,2,3,3a,4,5,6,8a-octahydro-	11.919	11.858	12.058	7746798	2.50	1925676	1.77	4.02

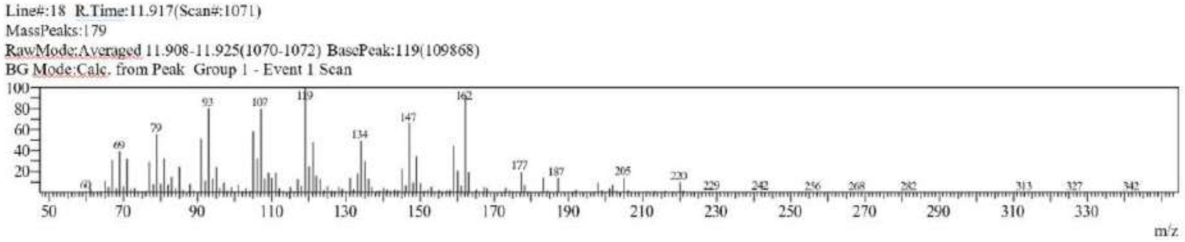									
19	Hexadecanoic acid, methyl ester	13.250	13.158	13.308	14167880	4.58	7296800	6.71	1.94
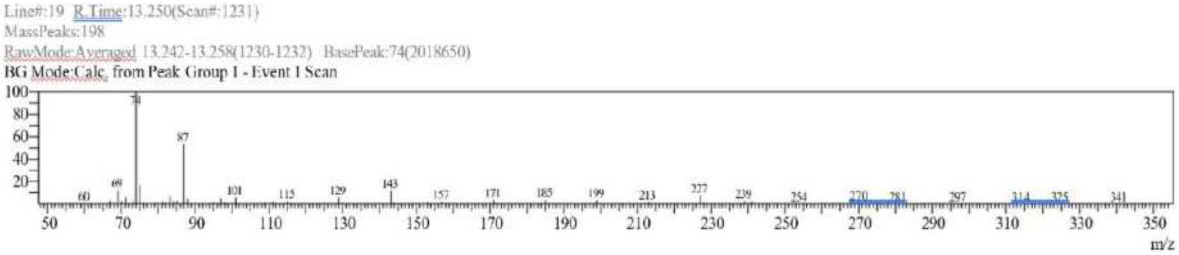									
20	n-Hexadecanoic acid	13.623	13.533	13.683	37636296	12.16	16578505	15.25	2.27
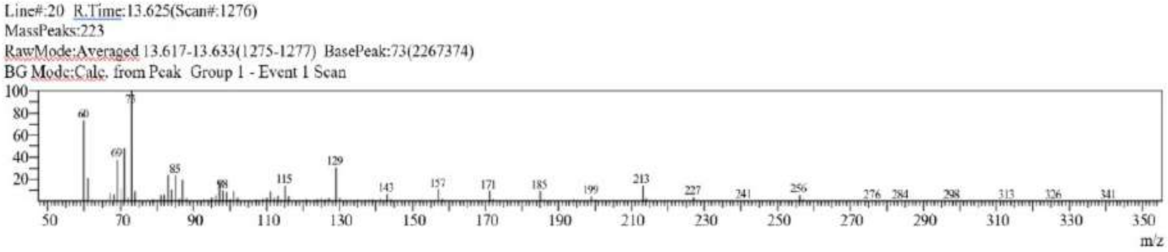									
21	Hexadecanoic acid, ethyl ester	13.905	13.875	13.950	1121355	0.36	648383	0.60	1.73

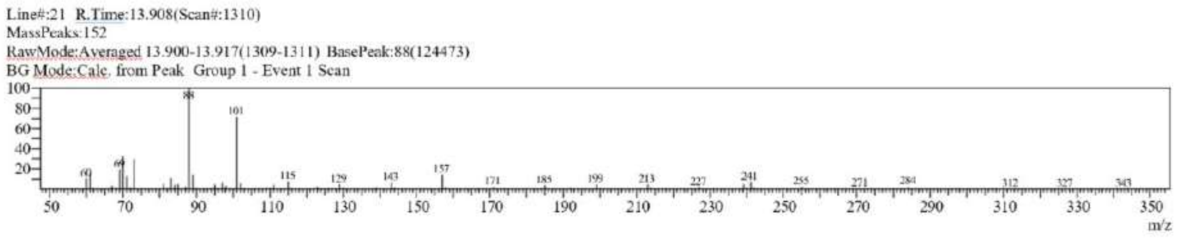									
22	12,15-Octadecadienoic acid, methyl ester	14.857	14.800	14.883	13581838	4.39	7138049	6.57	1.90
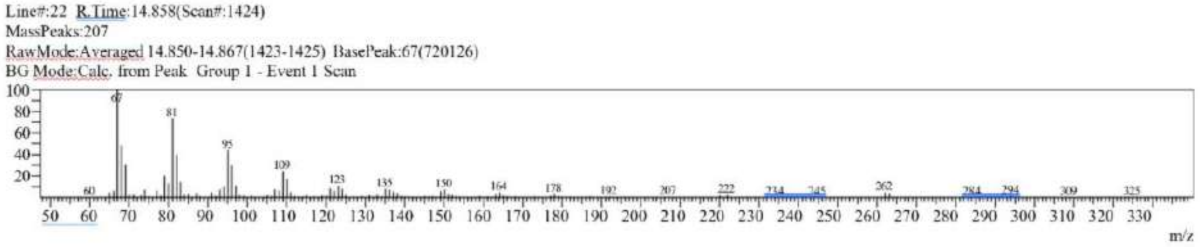									
23	9,12,15-Octadecatrienoic acid, me-thyl ester, (	14.915	14.883	14.967	33480976	10.81	17993130	16.55	1.86
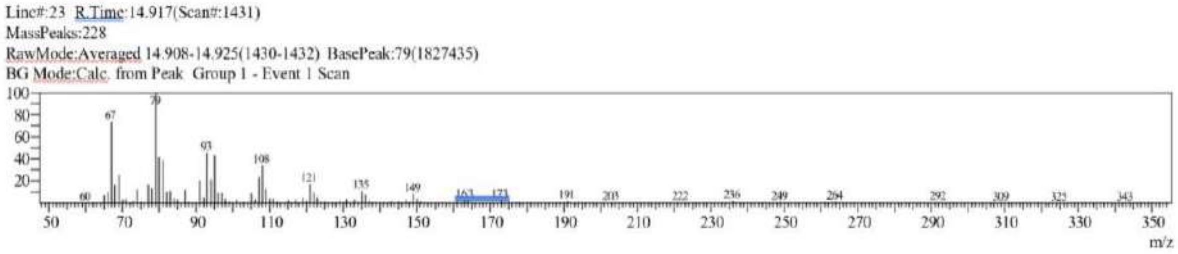									
24	Phytol	15.152	14.967	15.200	30283797	9.78	6573053	6.05	4.61

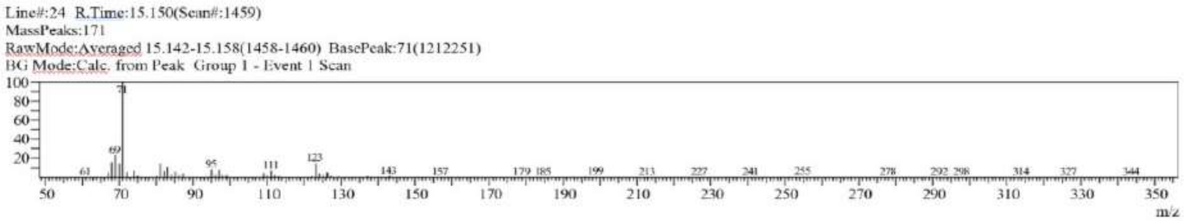

25	10E,12Z-Octadecadienoic acid	15.234	15.200	15.258	15444369	4.99	5190259	4.77	2.98

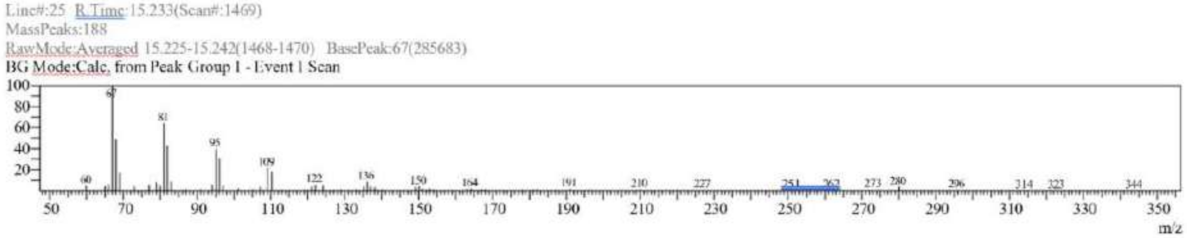

26	9,12,15-Octadecatrienoic acid, (Z,Z,Z)-	15.296	15.258	15.425	33542264	10.83	12318614	11.33	2.72

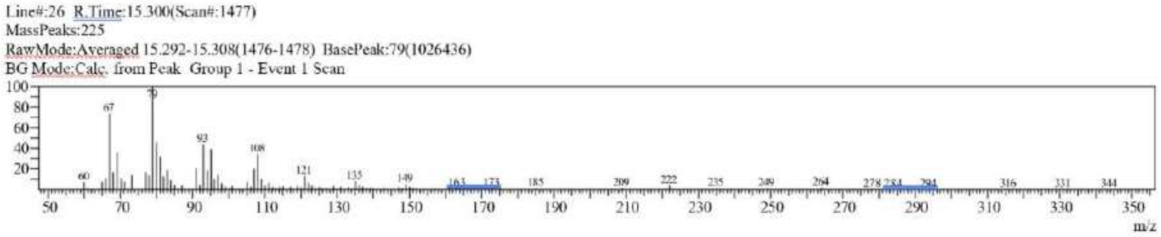

27	Octadecanoic acid	15.490	15.425	15.567	5974806	1.93	1496316	1.38	3.99

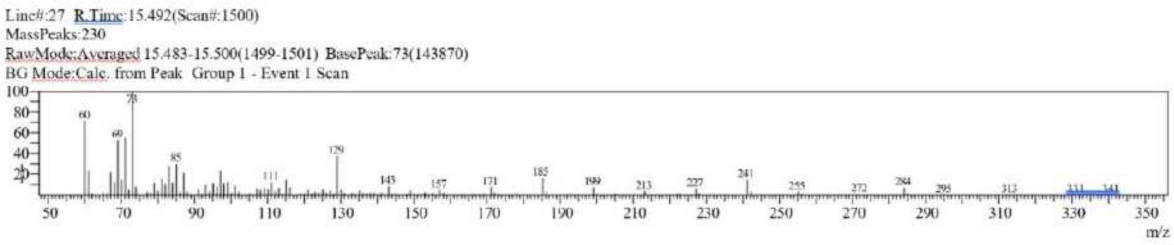									
28	9-Octadecenamide, (Z)-	17.228	17.183	17.275	1711098	0.55	750292	0.69	2.28
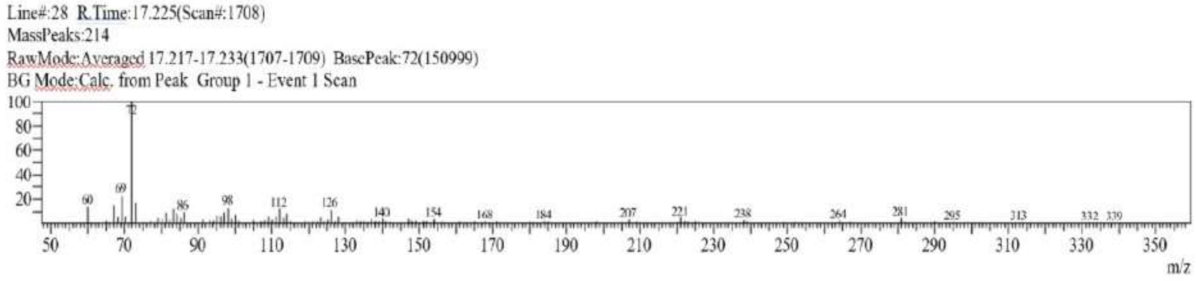									
29	Bis(2-ethylhexyl) phthalate	18.588	18.542	18.642	3686617	1.19	1836349	1.69	2.01
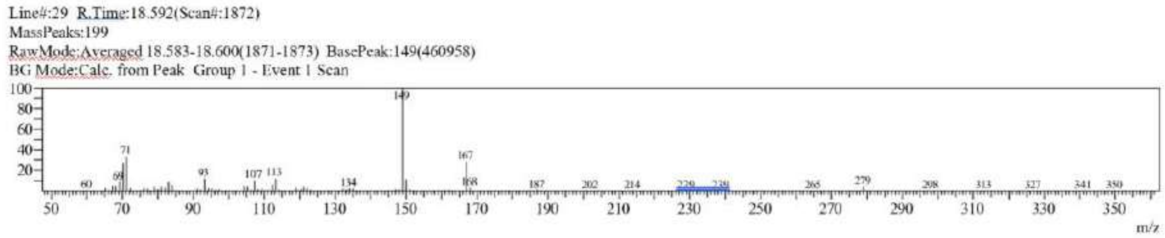									
30	2,2-Dimethyl-3-(3,7,16,20-tetrame-thyl-heneic	19.542	19.492	19.608	3289746	1.06	1302566	1.20	2.53

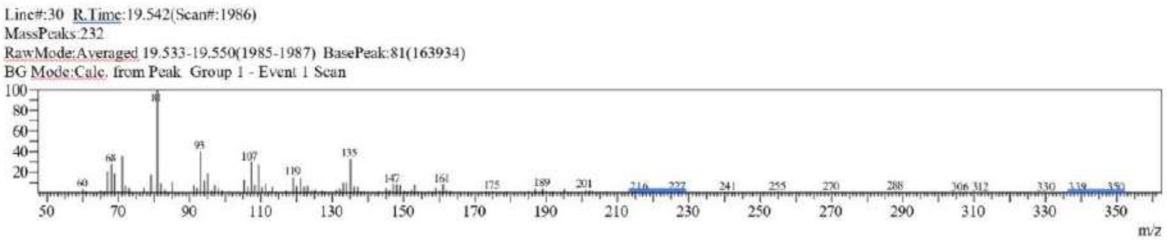

31	.alpha.-Methyltyrosine, N,O,O'-tris(tert-butyld	21.180	21.100	21.308	6983824	2.26	1272977	1.17	5.49

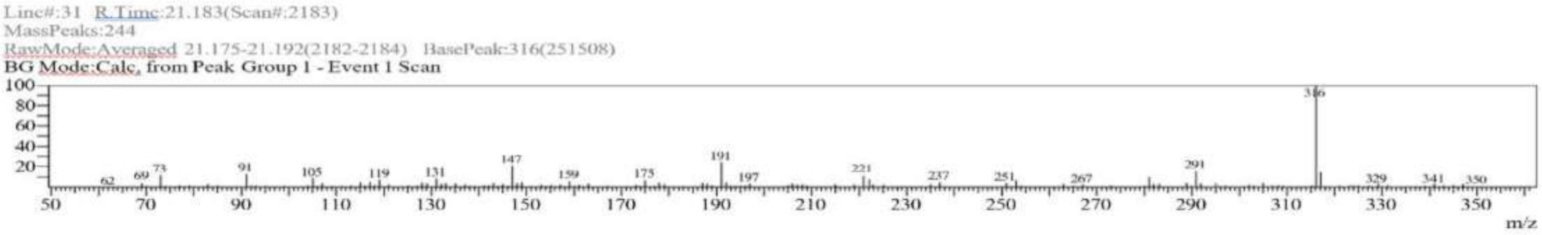

Supplement: Supplementary file 1 [file pharmaceuticals-18-00288-s001.zip › pharmaceuticals-3417799-Table S2.pdf]
